# Proteome-wide prediction of bacterial carbohydrate-binding proteins as a tool for understanding commensal and pathogen colonisation of the vaginal microbiome

**DOI:** 10.1038/s41522-021-00220-9

**Published:** 2021-06-15

**Authors:** François Bonnardel, Stuart M. Haslam, Anne Dell, Ten Feizi, Yan Liu, Virginia Tajadura-Ortega, Yukie Akune, Lynne Sykes, Phillip R. Bennett, David A. MacIntyre, Frédérique Lisacek, Anne Imberty

**Affiliations:** 1 University Grenoble Alpes, CNRS, CERMAV, Grenoble, France; 2 Swiss Institute of Bioinformatics, Geneva, Switzerland; 3 Computer Science Department, UniGe, Geneva, Switzerland; 4 Department of Life Sciences, Imperial College London, London, UK; 5 March of Dimes European Prematurity Research Centre, Imperial College London, London, UK; 6 Glycosciences Laboratory, Department of Metabolism Digestion and Reproduction, Imperial College London, London, UK; 7 Imperial College Parturition Research Group, Division of the Institute of Reproductive and Developmental Biology, Department of Metabolism Digestion and Reproduction, Imperial College London, London, UK; 8 Queen Charlotte’s Hospital, Imperial College Healthcare NHS Trust, London, UK; 9 Tommy’s National Centre for Miscarriage Research, Imperial College London, London, UK; 10 Section of Biology, UniGe, Geneva, Switzerland

**Keywords:** Bacteria, Pathogens

## Abstract

Bacteria use carbohydrate-binding proteins (CBPs), such as lectins and carbohydrate-binding modules (CBMs), to anchor to specific sugars on host surfaces. CBPs in the gut microbiome are well studied, but their roles in the vagina microbiome and involvement in sexually transmitted infections, cervical cancer and preterm birth are largely unknown. We established a classification system for lectins and designed Hidden Markov Model (HMM) profiles for data mining of bacterial genomes, resulting in identification of >100,000 predicted bacterial lectins available at unilectin.eu/bacteria. Genome screening of 90 isolates from 21 vaginal bacterial species shows that those associated with infection and inflammation produce a larger CBPs repertoire, thus enabling them to potentially bind a wider array of glycans in the vagina. Both the number of predicted bacterial CBPs and their specificities correlated with pathogenicity. This study provides new insights into potential mechanisms of colonisation by commensals and potential pathogens of the reproductive tract that underpin health and disease states.

## Introduction

Microbiota–host interactions within different ecological niches of the human body are critical determinants of health and disease states^1^. At mucosal surface interfaces, microbial and host cells, as well as non-cellular components of the mucosa, present an exceptionally complex array of attachment and recognition sites for microbiota, many of which are carbohydrate sequences displayed on extensively glycosylated mucin-type glycoproteins rich in O-glycans. The diverse populations of glycans provide recognition sites for adhesive proteins of microbiota that have the ability to distinguish the various motifs displayed. Bacteria also produce glycosylhydrolases and other enzymes that facilitate the use of secreted mucins as the primary carbon sources for energy metabolism^2,3^. The abilities of microbes to specifically recognise, attach and adhere to cellular and non-cellular sites are thus key aspects of commensal and pathogenic colonisation, and are mediated by receptors such as lectins and carbohydrate-binding modules (CBMs)^3–6^.

Lectins are ubiquitous proteins of non-immune origin that bind to a variety of carbohydrates without modifying them^7^. Through their interactions with glycoproteins and glycolipids via the oligosaccharides, lectins play crucial roles in cell–cell communication, signalling pathways and immune responses^8^. Bacterial lectins may be incorporated into multiprotein organelles, such as fimbriae (pili) or flagellae, and participate in the mediation of host recognition and adhesion^9^. In pathogenic species, lectins may also be subunits associated with a toxic catalytic unit that target subcellular components^10^. Soluble lectins are also expressed as virulence factors by opportunistic bacteria^11^ and can alter dynamics of glycolipids to induce the internalisation of whole bacteria into host cells^12^. Bacterial lectins have also been shown to directly impair immune signalling and repair pathways, and are implicated in the formation of biofilms^13^.

The role of lectins and their ligands in shaping microbial niches in the human body is increasingly recognised, particularly at mucosal interfaces including the gut^3,14,15^ and oral cavity^16^. However, much less is known about the role of lectins in shaping microbial niches in the lower female reproductive tract, which play a key role in shaping health and disease throughout a woman’s life span^17^. Colonisation of the vagina by *Lactobacillus* species has long been considered a hallmark of health^18,19^, with the exception of *Lactobacillus iners*, which is often associated with dysbiosis and disease^20,21^. *Lactobacillus*-deplete, high-diversity vaginal microbiomes enriched in potential pathogens are characteristic of bacterial vaginosis and are associated with increased risk of sexually transmitted infections (STIs)^22,23^, progression of cervical cancer^24,25^ and adverse pregnancy outcomes such as miscarriage and preterm birth^26–29^. Key components of the vaginal mucosa are highly glycosylated mucins that are derived from the mucin-secreting glands of the cervix^30^. Alteration of terminal glycan residues of mucins by microbially secreted sialidases and sulphatases may modulate the physical and immunological properties of the vaginal mucosa^31^. Vaginal pathogens such as *Gardnerella vaginalis*, *Trichomonas vaginalis*, *Prevotella* and *Ureaplasma* species are capable of degrading secretory IgA^32–35^. Moreover, specific strains of *Streptococcus agalactiae* (group B streptococci) secrete hyaluronidases that degrade cervical hyaluronic acid into disaccharide fragments dampening host immune activation through inhibition of Toll-like receptors and thereby may contribute to preterm birth via ascending infection^36^. *S. agalactiae* can also implement a negative signalling mechanism known as sialoglycan mimicry to evade detection and phagocytosis by neutrophils; this is through terminal α2-3-linked sialic acids on the bacteria recognised by the neutrophil lectin Siglec-9 as ‘self’ glycan^37^.

Despite their important role in infection and pathogenicity, the full extent of the contribution of bacterial lectins and CBMs to health and disease states is yet to be fully elucidated. This is partly due to the limited annotation and characterisation of lectins in protein and proteome databases, which precludes predictions of the diversity, structure and function of the carbohydrate-binding proteins (CBPs). In recent years, this has begun to be addressed through the development of databases for structural and functional glycobiology^38,39^. Among these, UniLectin3D provides three-dimensional (3D) structures of more than 2500 lectins and their complexes with carbohydrates^40^ within UniLectin, a platform dedicated to the curation and collection of lectin knowledge accessible in several complementing modules. Manual selection of lectin domains in 3D structures permitted the identification of lectin classes characterised by fold similarity and minimum thresholds of sequence identity, and the defined amino acid sequence motifs and profiles characterising each lectin class can be used to screen proteomes and translated genomes to identify unannotated lectins in the LextomeXplore module^41^. We now apply this approach to bacterial lectins. Comparisons of these lectins across different vaginal microbiota strains provide new insights into the potential mechanisms by which colonisation by commensals and potential pathogens is associated with physiological and pathological conditions in the lower reproductive tract.

## Results

### Structural classification of lectins and status of bacterial lectins in UniLectin3D

**Fig. 1:**
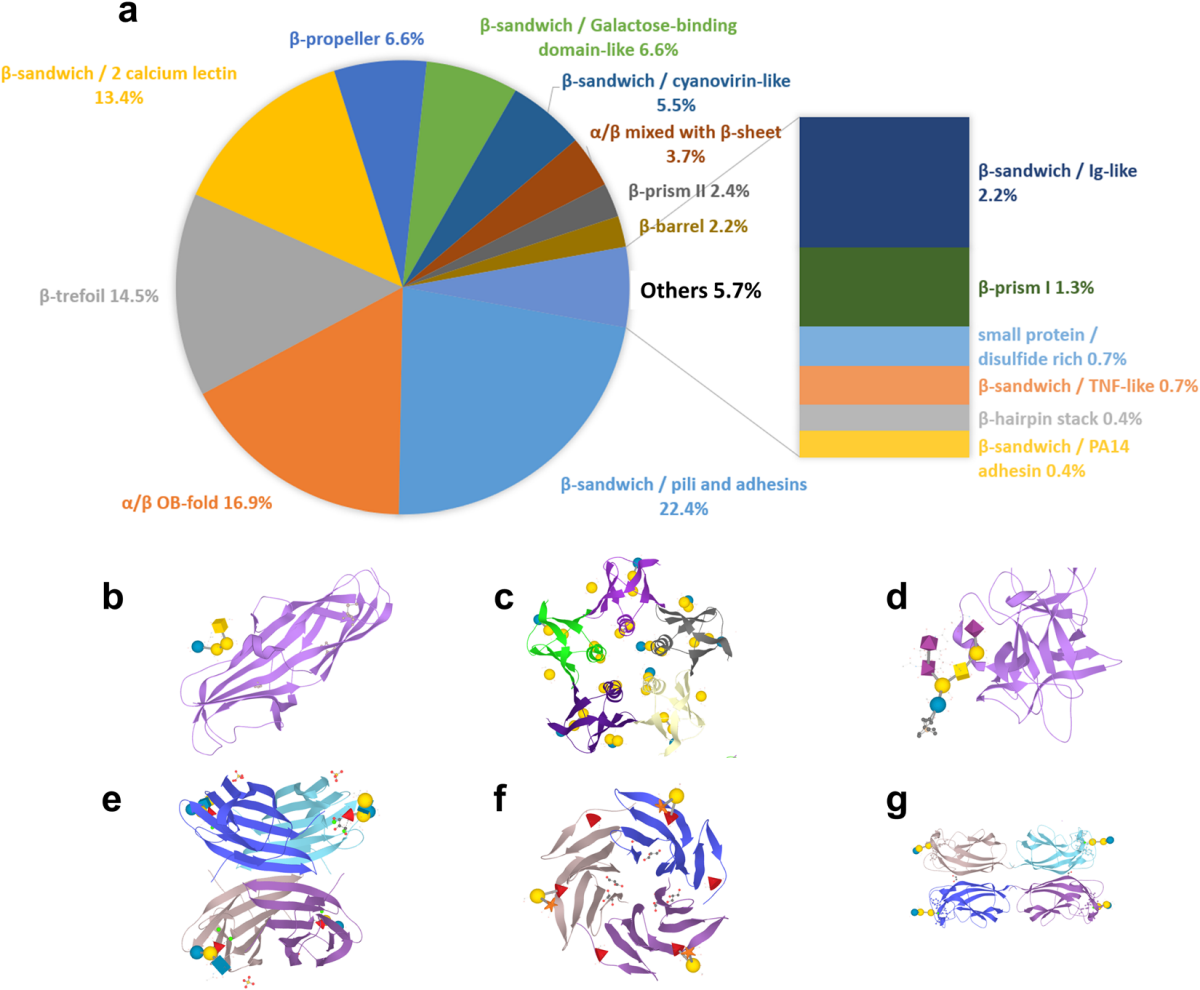
Bacterial lectin folds. **a** Distribution of bacterial lectin folds derived from the UniLectin3D database. From the analysis of fold distribution of bacterial lectin crystal structures, the six most frequent folds are detailed. **b** β-Sandwich/pili and adhesins fold representative: *Escherichia coli* PapG in complex with GalNAc(β1-3)Gal(α1-4)Gal(β1-4)Glc (PDB code 1J8R); **c** α/β OB fold: *E. coli* SLT-1 with Gal(α1-4)Gal(β1-4)Glc (1BOS); **d** β-Trefoil fold: *Clostridium tetani* TeNT with GT1b ganglioside NeuAc(α2-3)Gal(β1-3)GalNAc(β1-4)[NeuAc(α2-8)NeuAc(α2-3)]Gal(β1-4)Glc (1FV2); **e** β-Sandwich/2 calcium lectin fold: *Pseudomonas aeruginosa* LecB with Gal(β1-4)GlcNAc(β1-3)Gal(β1-4)[Fuc(α1-3)]Glc (1W8F); **f** β-Propeller fold: *Ralstonia solanacearum* RSL with Fuc(α1-2)Gal(β1-2)Xyl (2BS6); and **g** β-Sandwich with galactose-binding domain-like fold: *P. aeruginosa* LecA/Gal(α1-3)Gal(β1-4)Glc (2VXJ). 3D structures were generated using LiteMol^85^ with terminal monosaccharides at binding sites represented using Symbol Nomenclature for Glycans (SNFG)^86^.

The need for a structural classification of lectins was introduced and discussed previously^42^, and this is now implemented in the curated UniLectin3D database (www.unilectin.eu/unilectin3D/). Protein fold, i.e., the structure of the protein backbone, was primarily selected as the main criterion for grouping lectins. A total of 35 distinct folds were identified in the 2278 UniLectin3D entries. Then, a hierarchical classification was built upon amino acid sequence comparison. Within each fold category, 109 lectin classes (Supplementary Table 1) were defined at a 20% sequence similarity threshold. UniLectin3D is mostly composed of lectins originating from plants, fungi and animals. Bacterial lectins from 46 different species account for ~21% of database entries (499/2278), distributed among 16 different folds (Fig. 1) and 37 classes (Supplementary Table 1).

The analysis of fold distribution in bacterial lectin crystal structures showed an over-representation of β-sheet-containing folds, common to adhesins and toxins including previously described pili adhesins, such as FimH in uro-pathogenic *Escherichia coli*, the oligomer-binding (OB) fold of the cholera toxin-binding domain, the β-sandwich of LecA and LecB in *Pseudomonas aeruginosa*, and the β-trefoil of the recognition domain in clostridial neurotoxins^43^. Whereas the majority of folds found in bacterial lectins are shared with lectins of other origins, the β-sandwich/pili and adhesins fold, and derived classes of pili adhesins, as well as the αβ/OB fold and derived classes of AB_5_ toxins, are restricted to bacteria.

### Prediction of lectin sequences in bacterial proteomes

A variable proportion of coding genes in each newly sequenced bacterial genome is assigned through features automatically based on protein family profiling tools. However, missing definitions of proper lectin profiles hinders automatic lectin annotation. The alignment of amino acid sequences in each of the 109 lectin classes was performed to define 109 Hidden Markov Model (HMM) profiles, reflecting 109 characteristic motifs of conserved residues. These profiles were then used to screen 130 million bacterial protein sequences from the UniProt database (June 2020) and over 168 million bacterial protein sequences from the NCBI RefSeq database (Sept 2020) derived from more than 100,000 bacterial species. Two classes were excluded due to an exceedingly large number of predicted chi-lectin TIM-like (named after triosephosphate isomerase) and VLR-like (named after the variable lymphocyte receptor). Chi-lectin TIM-like domains occur on glycosylhydrolases and the VLR domains have a broad variety of ligands. We considered it unlikely that they are all lectins. Therefore, 107 classes were considered from this point on. This resulted in the selection of 100,671 sequences as putative lectins in 10,126 distinct bacterial species (reduced to 46,322 sequences in 6425 distinct bacterial species when we demarcated a score of 0.25). A web interface dedicated to the exploration of these bacterial lectin candidates is available at www.unilectin.eu/bacteria/.

**Fig. 2:**
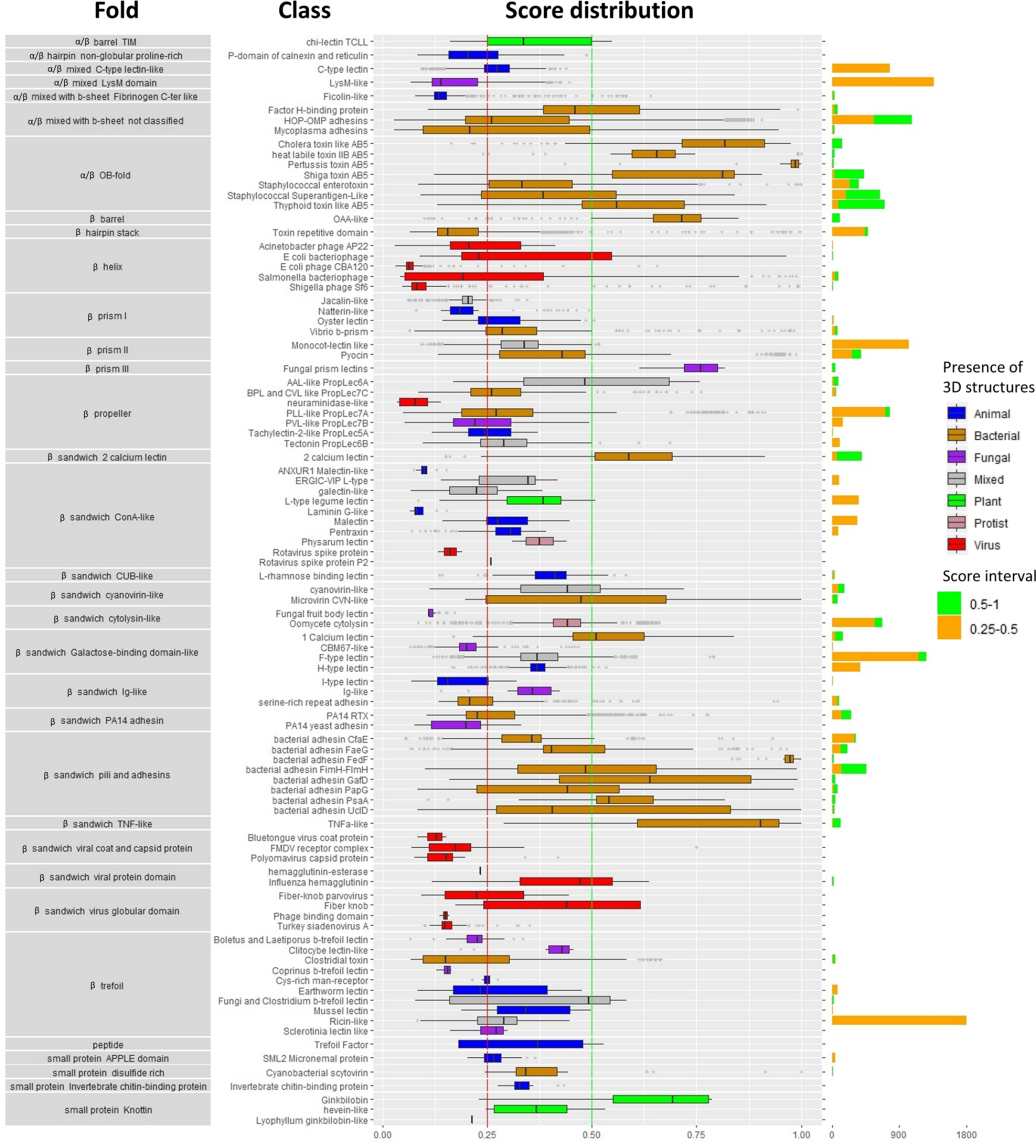
Distribution of structural folds in predicted bacterial lectins based on UniLectin3D lectin classes. The distribution of the predicted lectin classes is presented as horizontal boxes and whisker plots coloured on the basis of lectin class origin. The whisker plot represents the minimum, maximum, median, first quartile and third quartile in each class. Values approaching 1 are indicative of high sequence similarity to the reference motif. The predicted lectins in [0.25–0.5] and [0.5–1] score intervals are presented as bar graphs. The total number of predicted lectins in each class is listed in Supplementary Table 1.

Although the 499 3D structures of bacterial lectins in Unilectin3D are categorised into 37 classes (Fig. 1), the screening results indicate that the putative bacterial lectins occur in 97 out of the 107 identified classes (with a cut-off of 25% of similarity to the original profile) (Supplementary Table 1). Putative lectin sequences identified in each class, together with the distribution of the prediction scores relative to the original HMM motif, are presented in Fig. 2. This predicted distribution of folds and classes differs from that obtained when using 3D structures generated from the UniLectin3D database. Several classes are comparatively over-represented as shown by the tall orange bars on the right side of Fig. 2. These include the Ricin-like (β-trefoil fold), LysM-like (α/β mixed LysM fold) and F-type lectin (β-sandwich/galactose-binding domain-like fold) classes. Each lectin domain is predicted and scored by fitting the best HMM profile (see ‘Methods’). Lectins with the highest prediction scores were, as expected, of bacterial origin and included adhesins, AB_5_ toxins and calcium-dependent soluble lectins (brown boxes in Fig. 2). Nonetheless, screened bacterial sequences were found to match the β-prism III fungal lectin profile with a high score (rightmost purple box in Fig. 2) indicative of genetic exchange between bacteria and fungi, as observed elsewhere^44^. The majority of low scoring predictions (<0.25) reflective of low sequence similarity were those associated with viruses, with the exception of the influenza hemagglutinin, which contains a high abundance of sequences for the characteristic domain, although not all are carbohydrate-binding. Lectins with mid-range (0.25–0.5) prediction scores were evenly distributed across multiple genome sources.

### Identification and characterisation of vaginal microbiota lectins

**Fig. 3:**
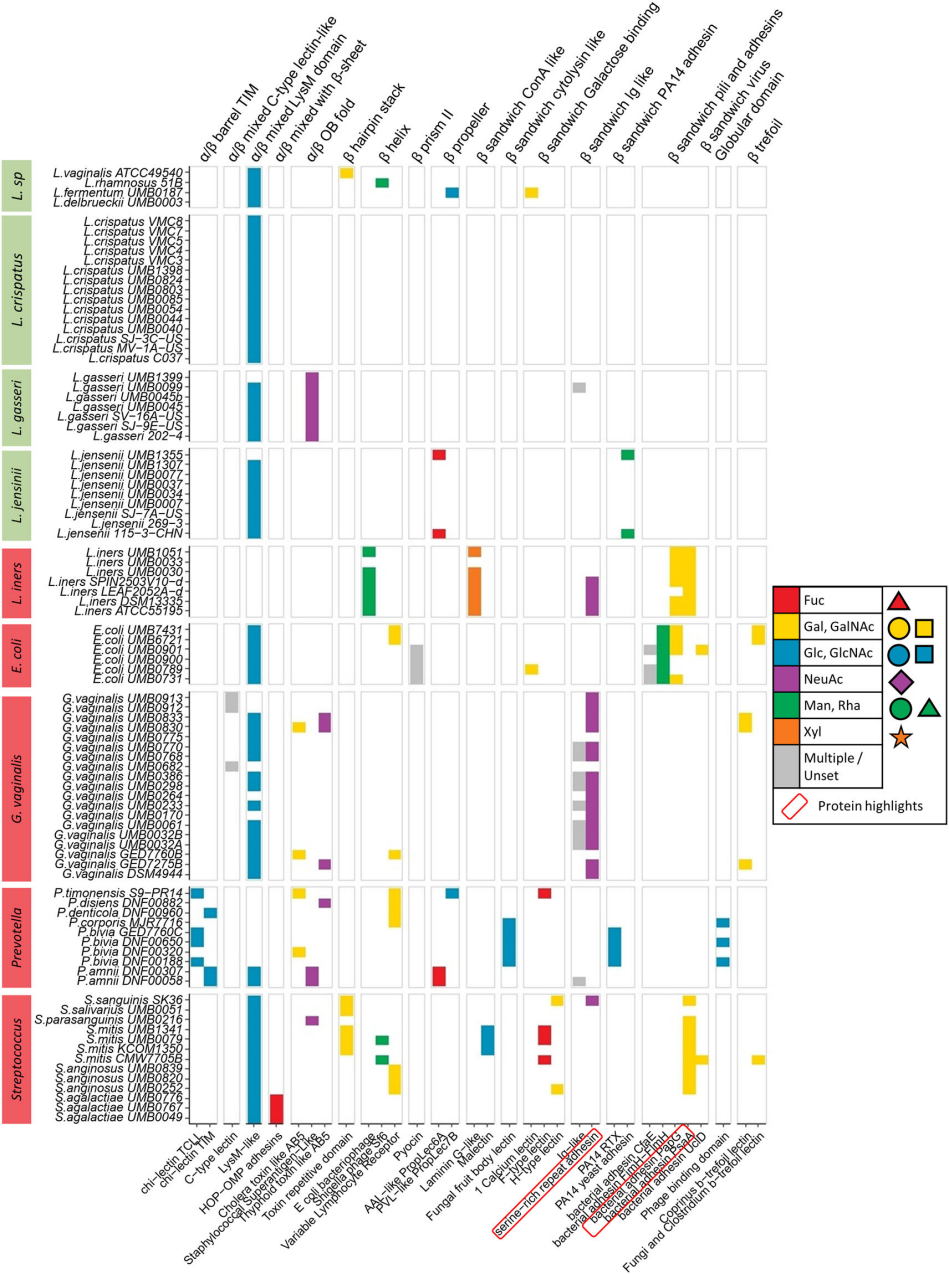
Distribution of predicted lectomes classified by fold and class in different vaginal commensal, and confirmed and potentially pathogenic bacterial species. In the margins, commensal species are indicated by green, and confirmed and potentially pathogenic species by red. Colours within each class of lectin reflect their predicted glycan-binding specificity, indicated as the monosaccharide with most contacts at the binding site of crystal structures available, and are represented using the Symbol Nomenclature of Glycans (SNFG) (https://www.ncbi.nlm.nih.gov/glycans/snfg.html). The lectin classes circled in red are further discussed in the ‘Results’ section to highlight their particular presence in *L. iners*. Accession numbers are listed in Supplementary Table 4.

The screening process, previously ran on bulk sequences from general-purpose databases UniProt and NCBI RefSeq, was applied to publicly available genome data of 90 vaginal bacterial strains classified on the basis of potential pathogenicity within the vaginal niche and having a known association with states of health or disease, resulting in the identification of 387 putative lectin sequences (Supplementary Table 2). Confirmed and potential pathogens, sometimes referred as pathobionts, were grouped and considered at the species level and include bacterial vaginosis-associated species given that they are associated with subclinical vaginal inflammation in some women^45,46^. Lactobacilli species as a whole are referred to here as ‘commensals’. This was in consideration of their high prevalence and relative abundance in the vaginal niche, their well-described role in providing protection from host infection through the production of antimicrobial and anti-inflammatory compounds, and their association with states of optimal vaginal health^18,47^. A comparison of the lectomes (i.e., the predicted set of lectins) highlighted major differences between the proportions of lectins observed in commensals, and confirmed and potential pathogens (Fig. 3). Of the total number of lectin classes (107), a significantly higher proportion was represented within the translated genomes of confirmed and potential pathogens compared to commensals (*P* = 4.602e^−^^05^, Fisher’s exact test). Similarly, the mean number of lectins per strain was significantly higher among those classified as confirmed and potential pathogens compared to commensals (pathobionts 5.34 ± 3.87, commensal 2.6 ± 2.6, *p* < 0.05 Student’s *T*-test). The most widespread lectin LysM, a common domain involved in cell wall attachment in many different bacteria, was predicted in the majority of vaginal microbial genomes examined, including all *Lactobacillus crispatus* isolates, but interestingly was absent from *L. iners* and most *Prevotella* strains.

Predicted lectins of *L. iners* could be mapped to five different classes: *E. coli* bacteriophage (β-helix fold), laminin G-like (β-sandwich/ConA-like fold), adhesin domain of two type 1 pili, PapG and PsaA (β-sandwich/pili and adhesins fold), and the adhesin domain of serine-rich repeat protein (SRRP) (β-sandwich/Ig-like fold). SRRP was also a prominent feature of *G. vaginalis* species and was identified in a *Streptococcus sanguinis* strain, whereas PapG is also found in *E. coli* and PsaA in several *Streptococcus* species. These five lectin domains did not feature in the commensal bacteria investigated and illustrate the unusual repertoire of *L. iners*. Up to ten different lectin classes were predicted in other *Streptococcus* species. Overall, the distinctive lectomes of the 90 vaginal bacterial strains, grouped according to their genus and potential pathogenicity, provide a further criterion strengthening this consistent grouping.

### Predictions of CBMs in vaginal microbiota

**Fig. 4:**
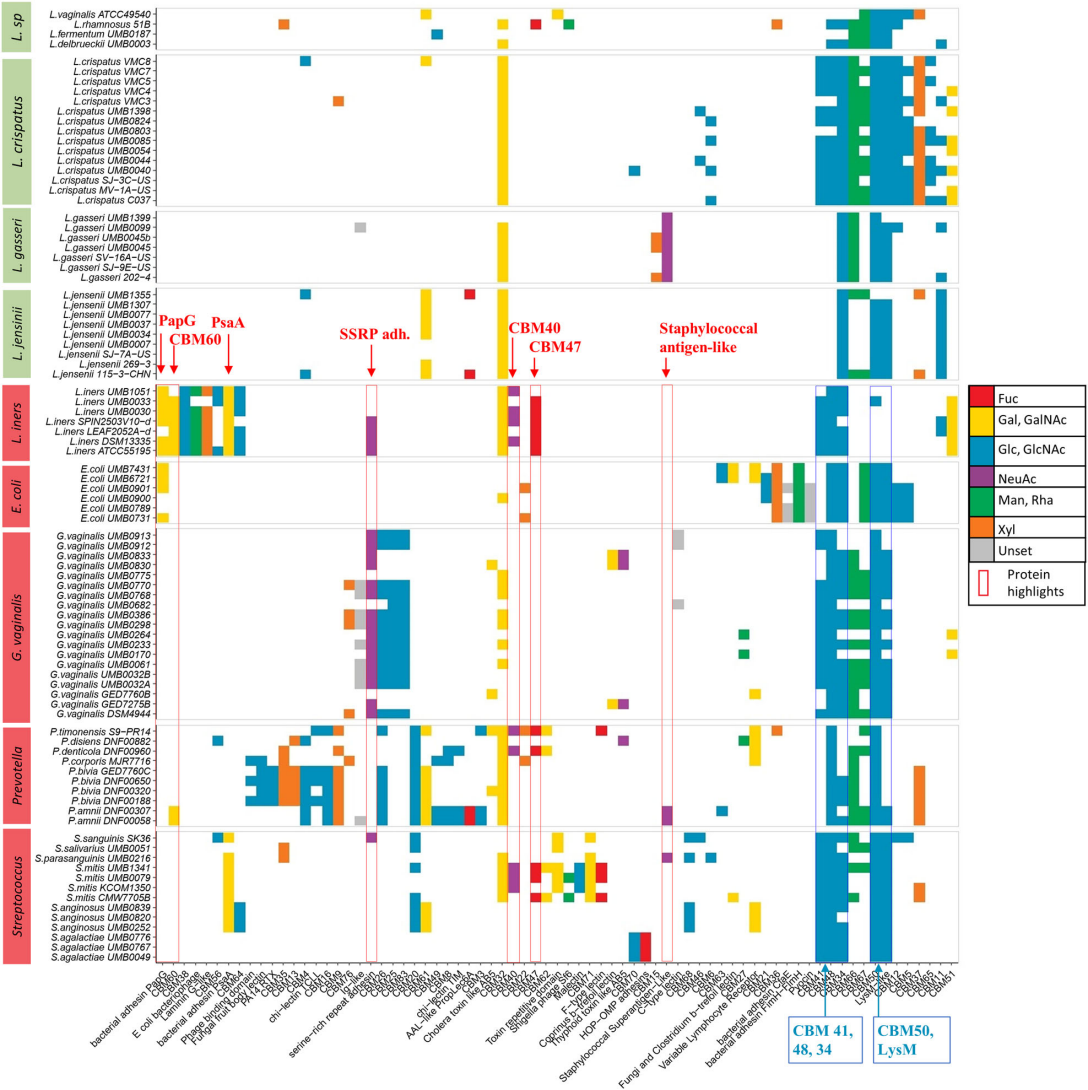
Distribution of predicted lectins and CBMs in different vaginal commensal, and confirmed and potentially pathogenic bacterial species arranged by domain composition similarity. Colours (following the SNFG nomenclature) within each class of lectins reflect its main sugar-binding specificities referred to in Fig. 3. The domains highlighted are further discussed in the results due to their presence in *L. iners*. The additional positive correlation of the number of CBMs distinguishes between commensal, and confirmed and potentially pathogenic bacteria. Accession numbers are available in Supplementary Table 4.

CBMs are different from lectins, as they occur as small domains generally associated with carbohydrate-modifying enzymes and often involved in microbial digestion of mucin glycans^48^. To fully assess the role of carbohydrate recognition in vaginal bacteria, screening was extended to the prediction of CBMs previously characterised with HMM profiles^49^. Using this approach that is compatible with the one we developed for lectins, 88 CBM motifs were searched in the 90 translated genomes of vaginal bacterial strains, revealing 1165 putative CBM sequences. The results suggest that predicted CBMs follow patterns similar to these for lectins, with a greater variety in confirmed and potential pathogens than in commensal strains (Fig. 4) as confirmed by Fisher’s test performed on classes of both lectins and CBMs (*P* = 1.027e^−08^). In addition, analysis of lectins and CBMs using principal component analysis highlighted increased variance and diversity associated with confirmed and potential pathogens strains (Supplementary Fig. 1).

CBM34, CBM41 and CBM48, which are specific for glucose-containing polysaccharides (e.g., amylose and glycogen) and generally act as binding modules for amylases and related enzymes, were consistently predicted across almost all vaginal species (Fig. 4). Although the majority of CBMs have been characterised as enzyme-associated domains in plant polysaccharides, two CBMs with specificity towards human glycan were observed in the dataset (Supplementary Table 3). The first, CBM40, is considered as sialic acid-specific, as it has been identified in association with a bacterial sialidase^50^. In the dataset analysed here, it is predicted to occur only in *L. iners* pathobiont species, *Streptococcus mitis* and some *Prevotella* species. Considering the earlier observation regarding the predicted SRR adhesin domain, the sialic acid-binding ability appears to correlate mainly with lectins and CBMs present in confirmed and potentially pathogenic bacteria. The second domain of interest is CBM47, shown to be fucose-specific in the lectin regulatory domain of a cholesterol-dependent cytolysin present in some *S. mitis* strains^51^. This domain has structure and sequence similarity with fish F-lectins^52^. In our study, this fucose-binding module is identified in *S. mitis* and in some pathobionts, i.e., *Provotella* and *L. iners*. Furthermore, two predicted galactose-specific adhesins, PapG and PsaA^53,54^, are part of the lectome of *L. iners* and their specificity for galactose is also shared by CBM60, which has a broad carbohydrate ligand specificity^55^. The complementary predictions of lectins and CBMs in vaginal bacterial have the same trends that distinguish confirmed and potentially pathogenic from commensal strains. This emphasises the importance and relevance of considering carbohydrate-binding as a strong functional feature in infection.

**Fig. 5:**
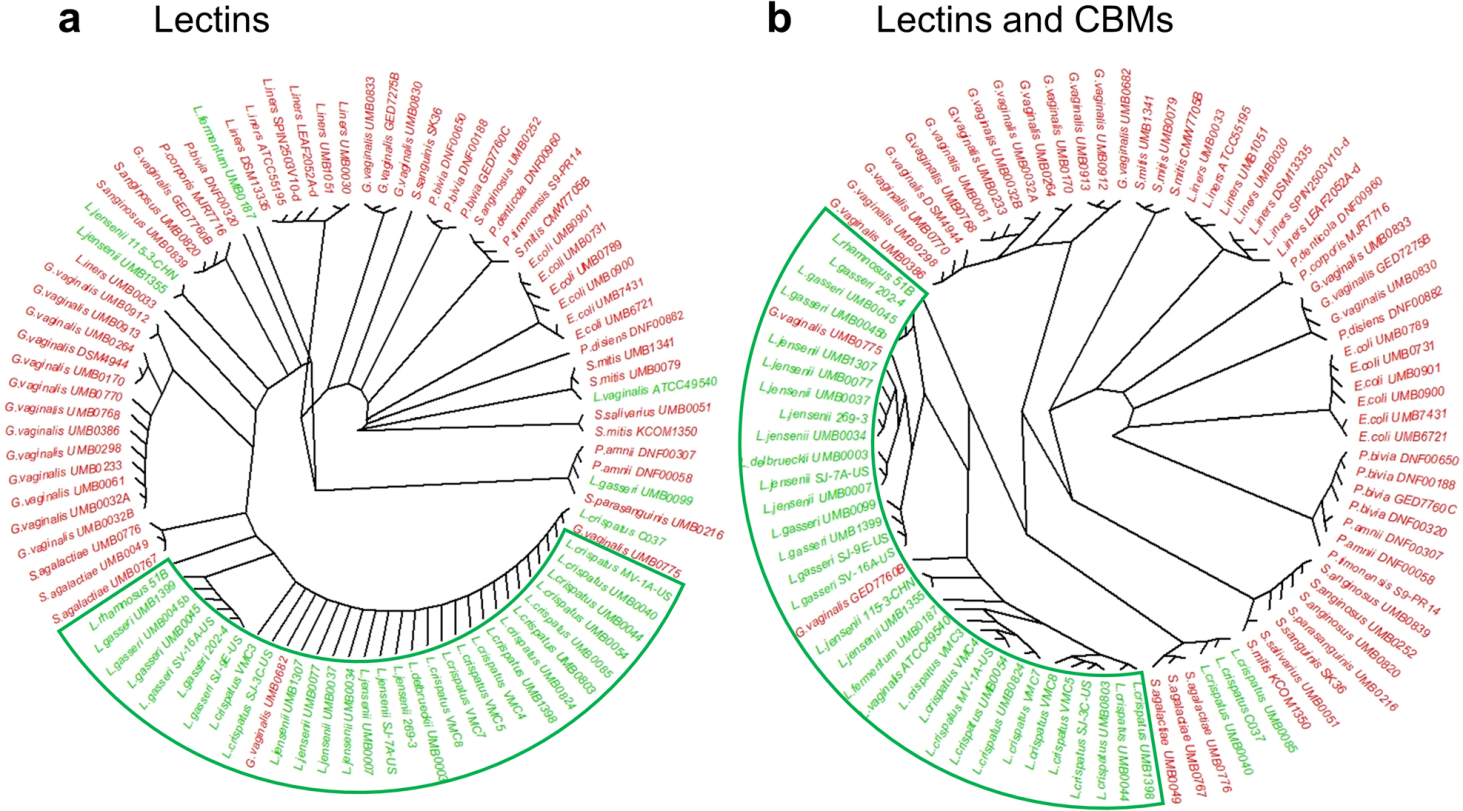
Hierarchical radial tree of predicted classes of carbohydrate-binding proteins in vaginal bacteria. (**a**) Predicted lectin classes only or (**b**) lectin classes and predicted carbohydrate-binding modules in vaginal commensal (green), and confirmed and potentially pathogenic (red) bacteria. The ubiquitous LysM and CBM50 are excluded from the dataset to generate the hierarchical radial tree. Although the majority of *Lactobacillus* species clustered closely to each other, indicating similar putative lectomes, the lectome of *L. iners* isolates more closely resembled that of confirmed and potential pathogens.

A further comparison of the predicted lectin and CBM profiles of vaginal commensals, and confirmed and potential pathogens was made by performing unsupervised hierarchical clustering on a Euclidean distance matrix of the number of proteins per species for each lectin and CBM domain (Fig. 5). The resulting hierarchical radial plot using predicted lectins showed a clear clustering of the majority of Lactobacilli, with further sub-clustering at species level observable. *L. iners* strains were an exception, as they clustered more closely with other pathobiont species including *Prevotella* and *Streptococcus* species (Fig. 5a). *G. vaginalis* also did not cluster in a single group. The inclusion of predicted CBMs in the clustering led to a finer discrimination between commensal, and confirmed and potentially pathogenic species; this serves to emphasise the species-specific dimension of clustering of the vast majority of isolates (Fig. 5b).

## Discussion

The contribution of bacterial CBPs to health and disease remains poorly understood. This is in part because their structural and functional complexity, and their limited annotations in protein and proteome databases have prevented the development of predictive models of structure, diversity and function. Here we begin to address this through manual selection of lectin domains in 3D structures obtained from the recently curated Unilectin3D database, followed by the prediction of lectin classes based upon fold similarity and minimum thresholds of sequence identity. This strategy has led to the identification of more than 35 different structural folds and 109 predicted lectin classes, of which 16 folds and 37 classes were of bacterial origin. These were particularly rich in β-sheet-containing folds, which have previously been recognised as key structural characteristics of lectins from non-bacterial origin^56^. Moreover, predicted classes of pili adhesins and AB_5_ toxins were found to be exclusive to bacteria. Although other lectin classes also appeared to be exclusively predicted in bacteria, these results are likely to be influenced by the fact that to date, many structurally characterised and curated lectins represent those of highest abundance in readily culturable bacteria.

The predictions of lectins in the bacterial proteomes pave the way to direct approaches, to identifying the proteins and determining their glycan-binding specificities as a lead to future designs of therapeutic molecules to target specific pathogenic bacteria. The predicted specificity provided in the present work is only indicative given the tolerance of carbohydrate-binding sites and their possible alteration through mutations. Nevertheless, such ‘predictions’ can guide further studies. Detailed knowledge of the glycan-binding specificities of the proteins is eagerly awaited by means of glycan array analyses of the whole bacterial cells and of determination of 3D structures of recombinantly expressed proteins in complex with their ligands.

Given the increased awareness of the importance of vaginal microbiome in shaping reproductive tract health outcomes, we next compared the occurrence of lectin-like proteins among vaginal commensal and potentially pathogenic bacteria isolated from the vagina. Our analysis suggests that the common commensal species, *L. crispatus* and *Lactobacillus gasseri*, produce LysM, which is a ubiquitous domain detected in almost all bacteria, but no other lectins among the 109 classes investigated here. Previous studies of this domain have shown that it can bind peptidoglycan with a specificity for *N*-acetylglucosamine^57^. CBM50, annotated in CAZy as binding *N*-acetylglucosamine residues, is another name of LysM and is therefore also widespread. The number of CBMs identified in the bacterial species investigated in the present study is small; these correspond mainly to domains associated with nutrient-degrading glycosylhydrolases. These results suggest that *Lactobacillus* species associated with optimal vaginal microbiome compositions appear to be comparatively ill-equipped for binding mucins. It is important to note that this observation may be biased, because the analysis only involved structurally characterised lectins. A limited number of other ‘mucin adhesion factors’ have been described in Lactobacilli^58,59^; however, except for the fimbriae domain in *Lactobacillus rhamnosus*^60^, these are in general described as moonlighting proteins, i.e., with adhesion properties being only a side activity in addition to their main function.

A shift from *Lactobacillus* species dominance of the vaginal niche towards increased bacterial diversity and enrichment of confirmed and potential pathogens are a signature of vaginal dysbiosis, which has been associated with a range of pathology states including increased risk of STIs^23^ and various poor pregnancy outcomes including miscarriage^26^. Yet, many of these confirmed and potential pathogens exist at low relative abundance levels within the vaginal microbiome of healthy, asymptomatic women^61,62^. The specific changes within the vaginal mucosal micro-environment that supports their expansion and colonisation remains poorly defined. We propose that the strategy for binding glycans has evolved more in vaginal confirmed and potential pathogens than in commensals, with the former producing a much larger variety of lectins and CBMs that enhances their capacity to adhere and bind to targets following disruption of the vaginal microbiome caused by menses^63^, contraceptives^64^, sexual activity^65^ or antibiotic use^66^. Our findings are consistent with previous reports on the occurrence of a large number of lectin domains in different species of streptococci; these participate in the architecture of toxins, adhesins and pilins^67^. However, it is important to note that glycan-mediated interactions associated with confirmed and potential pathogens and commensals within the vaginal niche are likely to be strain-specific. For example, evidence suggests that some strains of *G. vaginalis* may be commensal, whereas others may have higher pathogenic potential^68^. Thus, it is important to caveat our findings and acknowledge that some of the strains analysed in our study designated as confirmed and potential pathogens may actually be commensal in other circumstances.

Whereas *Lactobacillus* species are considered hallmarks of optimal vaginal health, *L. iners* is considered a marker of a ‘transitional microbiome’ at the crossroads of vaginal health and disease^20,21^. The predicted lectomes of the various *L. iners* strains screened were found to contain a different and more diverse array of lectin-like domains than those in other Lactobacilli, and the same applies to our analyses of CBM-like domains. This is somewhat surprising considering that *L. iners* genome is much smaller than those of other Lactobacilli^20^. Several of these identified domains resemble proteins that are able to bind to glycans present on human mucins; examples are sialic-binding domain from SRPPs, galactose-specific pilin domain, as well as fucose-binding CBMs usually associated with streptococci. *L. iners* shares some traits of pathogenicity with other pathogens, such as the presence of inerolysin, a pore-forming toxin from *L. iners* also found as vaginolysin in Gardnerella^69^. Moreover, sequences with similarity to fimbrial proteins PapG from *E. coli* and Psa/Myf from *Yersinia pestis* were identified in almost all strains of *L. iners*. Interestingly, these two adhesins have specificity towards galactosylated epitopes on glycolipids^53,54^.

The lectome expansion that appears to correlate with the transition towards species involved in vaginal dysbiosis raises the question of concomitant changes in vaginal glycans, perhaps in glyco-epitopes present on mucins. Mucin glycans have been well investigated in the gut and lung, and it has been demonstrated that glycosylation is altered in case of inflammation. For example, in cystic fibrosis patients, inflammation results in an increase in fucosylation and sialylation, favouring the attachment of opportunistic pathogens such as *P. aeruginosa*, which in turn stimulates the inflammatory process^70^. Such glycan-based processes may occur in the vagina and a deeper characterisation of mucin glycosylation in this context is needed.

Although the mechanisms underpinning dynamic shifts in vaginal microbial structure and composition remain to be fully elucidated, our study provides important new insights into CBP profiles of colonisation by commensals, and confirmed and potential pathogens of the reproductive tract that are associated with health and disease states.

The bioinformatics screening tools described and used in the present study can be run on any protein sequence data, to reveal information currently lacking on the content and the role of the lectome. Results show clearly the emergence of characteristic patterns indicative of pathological states. This may guide the development of new strategies for novel therapeutics designed to manipulate adhesion and attachment of microbes, to promote optimal colonisation of the lower reproductive tract.

## Methods

### Definition of signature profiles for lectins

A new lectin classification has been recently defined based on structural data and is available in the UniLectin3D database (https://unilectin.eu/unilectin3D/). The classification is built on three levels as follows: (1) the fold level directly derived from the protein 3D structure that describes the fold adopted by the whole lectin domain (β-helix, β-propeller and others). The nomenclature on fold are adopted from the reference structural-based databases, CATH^71^ and SCOPe^72^, and previous reports on structural classification of lectins^73^; (2) the class level defined by sequence similarity with a 20% cut-off between different classes, i.e., lectin sequences in one class are at least 20% similar to one another; (3) the family level defined at a minimum of 70% of sequence identity. The values of cut-offs were set in agreement with definitions in the CATH database for the class level and empirically for the family level, to maximise the consistency of each family. The classification is therefore organised in 35 folds, 109 classes and 350 families.

For each of the 109 lectin classes, UniLectin3D sequences were aligned with the Muscle software^74^ to construct a characteristic motif of conserved residues. Sequence redundancy was automatically removed. Manual inspection of characteristic lectin domains led to the creation of a list of disqualifying domains such as peptide tags to manage future systematic removal. Conserved regions from the multiple alignments were then fed to an HMM tool to generate profiles characterising each lectin class. The HMMER-hmmbuild tool^75^ was used to align each lectin class multiple sequence alignment against protein sequence datasets, with the sym_frac parameter at 0.8 to avoid isolated regions in the conserved motifs.

### Prediction of bacterial lectins in protein databases

Bacterial sequences recorded in UniProtKB^76^ and in non-redundant NCBI were processed with HMMER-hmmsearch, with default parameters and a *p*-value < 10^−2^, to run profiles obtained with HMMER-hmmbuild. Parameters include the BLOSUM62 score matrix for amino acid substitutions^77^. HMMER *p*-value threshold remains the most reliable parameter for trusting a candidate lectin. Further filtering was applied to multiple strains of the same species with almost identical proteins and only a few different amino acids due to natural mutation, sequencing errors or protein prediction errors. Post-processing involved keeping only one representative protein for all redundant proteins (from a same species with >98% of identity). Predicted domains with <15 amino acids are considered as small fragments.

Each sequence match output by the HMMER toolset is evaluated with a quality score used on the vaginal strain predicted lectins to assess their qualities. The HMM score has no upper boundary. Furthermore, as each family profile is generated independently of one another, quality scores are not comparable across motifs used for the prediction. This makes it impossible to use a single cut-off for all lectin classes. In addition, in the case of tandem repeat domains, the quality score is proportional to the number of repeats and artificially promotes sequences with repeated domains. To address these scoring issues, a prediction score for each database hit was defined to give the similarity between the predicted domain and the reference lectin motif. The amino acid sequence alignment generated by HMMER during the search is further evaluated: at each position of the alignment, a cumulative counter is incremented by 1 if amino acids are identical, else by a normalised BLOSUM62 substitution score. The final value of the counter divided by the domain length (i.e., the total number of positions) results in a value between 0 to 1 that defines the prediction/similarity score. A predicted lectin may belong to several classes, independently of the prediction score. The prediction/similarity score is mainly destined to order the information to be displayed on the UniLectin platform for each predicted lectin.

For each predicted protein, associated annotations are extracted and loaded from UniProt and from the NCBI. This includes the taxonomy details of the protein and the corresponding ID of the NCBI taxonomy database. Proteins considered as obsolete in the latest releases of UniProt or in the NCBI, with no associated metadata, are removed.

### Prediction of lectins and CBMs in the vaginal microbiome

The subset of bacteria corresponding to the vaginal microbiome (Supplementary Table 2) was identified from genome database annotations, such as those found in the Bioproject www.ncbi.nlm.nih.gov/bioproject/PRJNA316969 and from a published list of bacteria^78^. Bacteria belonging to different species of Lactobacilli, *Gardnerella*, *Prevotella*, *E. coli* and Group B *Streptococcus* were selected and classified into ‘commensals’ or ‘confirmed and potential pathogens’ on the basis of their potential pathogenicity within the vaginal niche as described recently^46^, as well as their known association with states of reproductive health and disease including bacterial vaginosis, preterm birth and risk of acquisition of STIs^18,19,23,28,46,79–81^.

The proteome of each strain was downloaded from the NCBI assembly database^82^. The corresponding sequences were processed to detect lectins and CBMs with the same method of prediction involving the 109 lectin profiles generated as described above. Considering the low number of identified lectins, the TIM lectin and the VLR classes were kept, despite a low probability of lectin activity. HMMER-hmmsearch was run to identify the lectome of each strain’s proteome with default parameters and a *p*-value < 10^−2^ with no further filtering. Proteins producing good quality alignments (HMM score > 50) with HMMER during the analysis of amino acid sequences were directly tagged as lectin domains. For lesser quality alignments, the ‘Align Sequences Protein BLAST’ component of the BlastP tool^83^ was used with default parameters to align a predicted domain against the closest reference lectin with a defined 3D structure. Manual quality checks, especially focused on the glycan-binding pocket, were carried out to verify the amino acid conservation and ensure the quality of the predicted lectin.

HMM profiles of CBMs were extracted from dbCAN2, a web server for the identification of carbohydrate-active enzymes^49^. The HMM profiles provided by dbCAN2 are based on CAZy CBM sequence data^84^. These profiles were used to identify 1777 proteins from the predicted proteomes of the vaginal commensals, and confirmed and potential pathogens. Following removal of high-frequency influenza-like predicted lectins and CBD domains occurring in less than three strains, the resulting data were grouped by domain clustering to reflect compositional similarities. The remaining CBMs were associated with their matching glycans and additional information (Supplementary Table 3).

To reinforce the results, influenza-like predicted lectins are removed (the high frequency of this domain is misleading, as mentioned earlier), and the lectin and CBM domains occurring in less than three strains were filtered out (removing 20 lectin classes and 15 CBM domains).

The following libraries were used:

1. Graphics were generated with R libraries of the Comprehensive R Archive Network including the *d3heatmap* package for heatmaps.
2. Hierarchical clustering: the Ward’s minimum variance method part of the *hclust* R package was used to process a Euclidean distance matrix of the number of predicted proteins per species for each domain.
3. GGplot2 and the APE (Analyses of Phylogenetics and Evolution) package for the hierarchical tree. In this case, prior clustering was applied to the data with the complete linkage method of the *hclust* R package. A Euclidean distance matrix of the number of predicted proteins per species for each domain was input.

The lectin and CBM specificities for glycans were manually recovered using UniLectin3D database and CAZy database annotations. Only predicted bacterial lectins with a score > 0.25 are kept.

### Reporting summary

Further information on research design is available in the Nature Research Reporting Summary linked to this article.

## Supplementary information

Supplementary Information

Reporting Summary

## Data Availability

Data are publicly available in the database unilectin.eu and all other data supporting the findings of this study are available within the paper and its Supplementary Information files.
