# Supplementary material for: Proteome-wide prediction of bacterial carbohydrate-binding proteins as a tool for understanding commensal and pathogen colonisation of the vaginal microbiome
Source: NPJ Biofilms Microbiomes. 2021 Jun 15;7:49. doi: 10.1038/s41522-021-00220-9 (PMC8206207; doi:10.1038/s41522-021-00220-9)
Supplement: Supplementary file 1 — Supplementary Information [file 41522_2021_220_MOESM1_ESM.pdf]

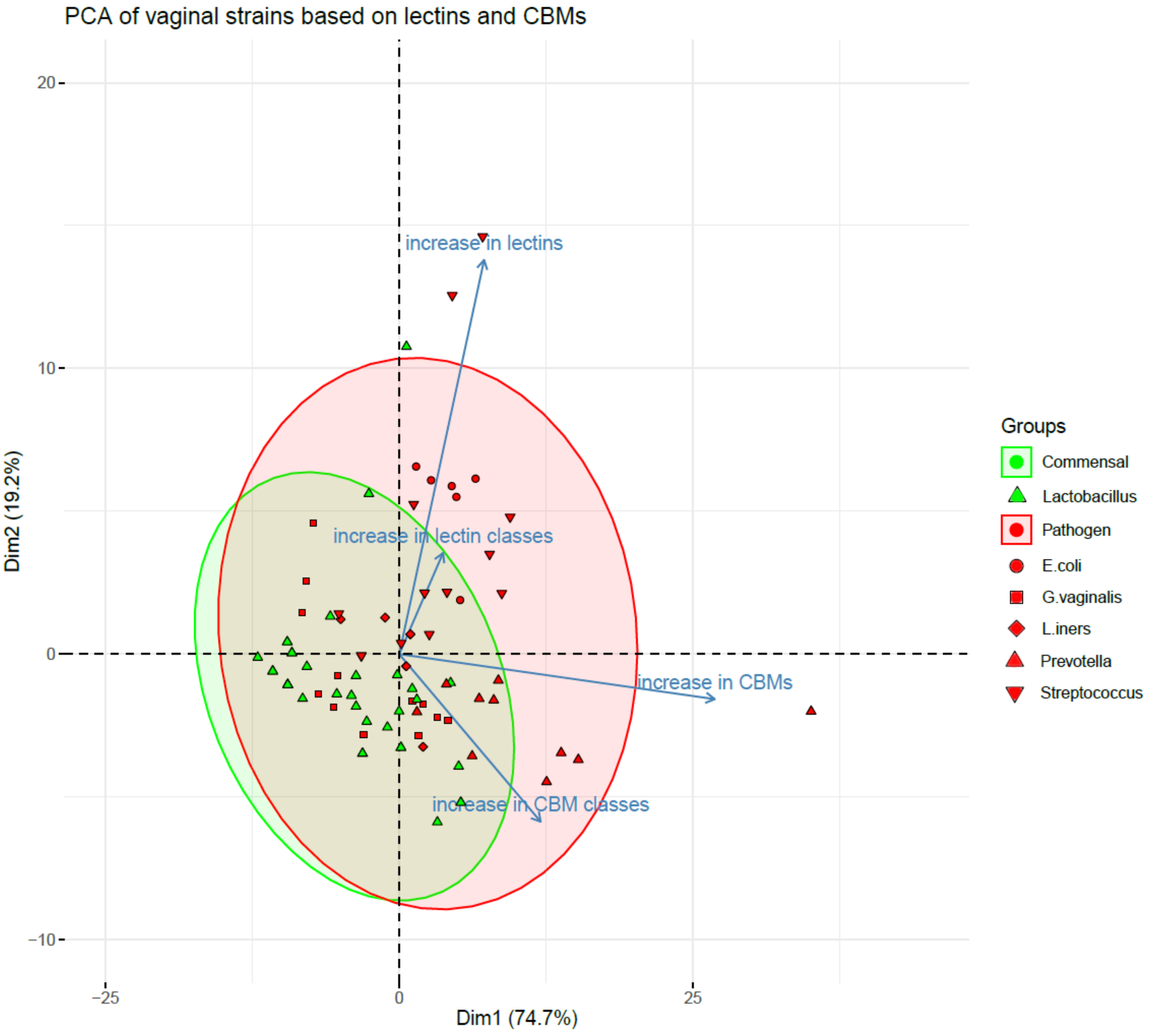

Supplementary Figure 1: PCA of the selected vaginal strains based on predicted lectins and CBMs, by number of proteins and number of distinct classes. The ellipse represent the 95% interval of confidence showing that multiple pathogen species are outside of the commensal confidence ellipse, highlighting the positive correlation with the identified lectins and lectin classes.

Supplementary Table 1: List of lectin classes identified from Unilectin3D and used in the classification. The lectin specificities for glycans were manually recovered using UniLectin3D database (www.unilectin.eu).

Lectin class	*Fold*	*known 3D structure*	*Bacterial structures in UniLectin3D*	*Terminal sugar recognised*	*Bacterial predictions in specified score interval*			
					0-0.25	*0.25-0.5*	*0.5-1*	*Total*
chi-lectin TCLL	α/β barrel / TIM	Plant	None	β-GlcNAc	6	7	5	18
P-domain of calnexin and reticulin	α/β hairpin / non-globular proline-rich	Animal	None	α-Glc	15	7	0	22
C-type lectin	α/β mixed / C-type lectin-like	Animal	None	α-Fuc, α-Man	1088	2632	0	3720
LysM-like	α/β mixed / LysM domain	Fungal	None	β-GlcNAc	25201	4575	0	29776
Ficolin-like	α/β mixed with β-sheet / Fibrinogen C-ter like	Animal	None	β-GlcNAc	1015	46	25	1086
MAR Micronemal protein	α/β mixed with β-sheet / MAR_domain	Protist	None	α-Neu5Ac	0	0	0	0
Factor H-binding protein	α/β mixed with β-sheet / not classified	Bacterial	2	α-Gal	11	92	49	152
HOP-OMP adhesins	α/β mixed with β-sheet / not classified	Bacterial	15	α-Fuc	1754	1624	698	4076
Mycoplasma adhesins	α/β mixed with β-sheet / not classified	Bacterial		α-Neu5Ac	92	37	26	155
Cholera toxin like AB5	α/β OB-fold	Bacterial	33	β-Gal	3	15	156	174
heat labile toxin IIB AB5	α/β OB-fold	Bacterial	5	α-Neu5Ac	1	3	54	58
Pertussis toxin AB5	α/β OB-fold	Bacterial	3	α-Neu5Ac	0	1	28	29
Shiga toxin AB5	α/β OB-fold	Bacterial	20	α-Gal	18	102	502	622
Staphylococcal enterotoxin	α/β OB-fold	Bacterial	2	β-Gal	276	660	198	1134
Staphylococcal Superantigen-Like	α/β OB-fold	Bacterial	6	α-Neu5Ac	523	528	777	1828
Thyphoid toxin like AB5	α/β OB-fold	Bacterial	9	α-Neu5Ac	124	227	906	1257
Plasmodium Erythrocyte binding antigen	ahelix_triplets / Duffy-like	Protist	None	α-Neu5Ac	0	0	0	0
OAA-like	β-barrel	Bacterial	10	α-Man	14	18	141	173
P-type lectin	β-barrel	Animal	None	α-Man	0	0	0	0
P-type lectin-like	β-barrel	Animal	None	α-Man	0	0	0	0
Toxin repetitive domain	β-hairpin stack	Bacterial		α-Gal	7238	1395	48	8681
Acinetobacter phage AP22	β-helix	Virus	None	NA	52	30	0	82
E coli bacteriophage	β-helix	Virus	None	α-Rha	33	12	19	64
E coli phage CBA120	β-helix	Virus	None	α-Glc	860	4	2	866
Salmonella bacteriophage	β-helix	Virus	None	α-Rha	249	130	62	441
Shigella phage Sf6	β-helix	Virus	None	α-Rha	425	9	18	452
Jacalin-like	β-prism I	Mixed	None	α-Man	858	15	0	873
Natterin-like	β-prism I	Animal	None	α-Man	75	14	0	89
Oyster lectin	β-prism I	Animal	None	α-Man	68	58	1	127
Vibrio β-prism	β-prism I	Bacterial	12	α-Man	74	146	44	264
Monocot-lectin like	β-prism II	Mixed	None	α-Man	702	2955	6	3663
Pyocin	β-prism II	Bacterial	1	NA	162	679	186	1027
Fungal prism lectins	β-prism III	Fungal	None	α-Fuc	0	0	53	53
AAL-like PropLec6A	β-propeller	Mixed	None	α-Fuc	8	77	81	166
BPL and CVL like PropLec7C	β-propeller	Bacterial	2	NA	126	152	17	295
neuraminidase-like	β-propeller	Virus	None	α-Neu5Ac	5	0	0	5
PLL-like PropLec7A	β-propeller	Bacterial	15	α-Fuc	1632	2071	90	3793
PVL-like PropLec7B	β-propeller	Fungal	None	D-GlcNAc	668	417	0	1085
Tachylectin-2-like PropLec5A	β-propeller	Animal	None	D-GlcNAc	59	52	0	111
Tectonin PropLec6B	β-propeller	Mixed	None	α-Man	154	328	3	485
2 calcium lectin	β-sandwich / 2 ca lectin	Bacterial	62	α-Fuc	8	143	520	671
ANXUR1 Malectin-like	β-sandwich / ConA-like	Animal	None	D-GlcNAc	10	0	0	10
Coronavirus spike protein	β-sandwich / ConA-like	Virus	None	α-Neu5Ac	4	0	0	4
ERGIC-VIP L-type	β-sandwich / ConA-like	Mixed	None	α-Man	100	276	0	376
galectin-like	β-sandwich / ConA-like	Mixed	None	β-Gal	33	14	0	47
Laminin G-like	β-sandwich / ConA-like	Plant	None	β-Xyl	12	0	0	12
L-type legume lectin	β-sandwich / ConA-like	Animal	None	aMan, β-Gal	214	904	3	1121
Malectin	β-sandwich / ConA-like	Animal	None	α-Glc	381	1058	0	1439
Pentraxin	β-sandwich / ConA-like	Animal	None	NA	56	259	0	315
Physarum lectin	β-sandwich / ConA-like	Protist	None	NA	0	2	0	2
Rotavirus spike protein	β-sandwich / ConA-like	Virus	None	α-Neu5Ac	2	0	0	2
Rotavirus spike protein P2	β-sandwich / ConA-like	Virus	None	α-Neu5Ac	0	1	0	1
Yeast Emp L-type	β-sandwich / ConA-like	Fungal	None	NA	0	0	0	0
L-rhamnose binding lectin	β-sandwich / CUB-like	Animal	None	α-Rha	7	72	5	84
cyanovirin-like	β-sandwich / cyanovirin-like	Mixed	None	α-Man	62	228	136	426

Microvirin CVN-like	β-sandwich / cyanovirin-like	Bacterial	2	α-Man	49	42	84	175
Fungal fruit body lectin	β-sandwich / cytolysin-like	Fungal	None	D-GlcNAc	36	0	0	36
Oomycete cytolysin	β-sandwich / cytolysin-like	Protist	None	D-GlcN	46	1320	174	1540
1 Calcium lectin	β-sandwich / Galactose-binding domain-like	Bacterial	24	α-Gal	7	112	160	279
CBM67-like	β-sandwich / Galactose-binding domain-like	Fungal	None	α-Gal	1408	46	0	1454
F-type lectin	β-sandwich / Galactose-binding domain-like	Mixed	None	α-Fuc	303	3078	174	3555
H-type lectin	β-sandwich / Galactose-binding domain-like	Animal	None	α-GalNAc	15	1014	3	1032
Sea_anemon_lectin	β-sandwich / Galactose-binding domain-like	Animal	None	α-Gal	0	0	0	0
Ig-like	β-sandwich / Ig-like	Animal	None	NA	2	16	0	18
I-type lectin	β-sandwich / Ig-like	Fungal	None	α-Neu5Ac	144	53	0	197
serine-rich repeat adhesin	β-sandwich / Ig-like	Bacterial	10	α-Neu5Ac	733	243	24	1000
PA14 RTX	β-sandwich / PA14 adhesin	Bacterial	2	α-Glc	1179	358	237	1774
PA14 yeast adhesin	β-sandwich / PA14 adhesin	Fungal	None	α-Man	192	33	0	225
bacterial adhesin CfaE	β-sandwich / pili and adhesins	Bacterial	1	NA	156	851	22	1029
bacterial adhesin FaeG	β-sandwich / pili and adhesins	Bacterial	2	β-Gal	38	277	151	466
bacterial adhesin FedF	β-sandwich / pili and adhesins	Bacterial	3	NA	0	0	28	28
bacterial adhesin FimH-FlmH	β-sandwich / pili and adhesins	Bacterial	68	α-Man	130	338	451	919
bacterial adhesin GafD	β-sandwich / pili and adhesins	Bacterial	13	β-GlcNAc	3	22	41	66
bacterial adhesin PapG	β-sandwich / pili and adhesins	Bacterial	8	α-Gal	51	63	67	181
bacterial adhesin PsaA	β-sandwich / pili and adhesins	Bacterial	5	α-Fuc	2	17	58	77
bacterial adhesin UclD	β-sandwich / pili and adhesins	Bacterial	2	β-Gal	15	26	29	70
TNFα-like	β-sandwich / TNF-like	Bacterial	4	α-Fuc	0	23	125	148
Bluetongue virus coat protein	β-sandwich / viral coat and capsid protein	Virus	None	α-Neu5Ac	3	0	0	3
Equine_Rhinitis_A_Virus_Coat_Protein	β-sandwich / viral coat and capsid protein	Virus	None	α-Neu5Ac	0	0	0	0
FMDV receptor complex	β-sandwich / viral coat and capsid protein	Virus	None	α-Fuc	16	2	1	19
Polyomavirus capsid protein	β-sandwich / viral coat and capsid protein	Virus	None	α-Neu5Ac	14	2	0	16
hemagglutinin-esterase	β-sandwich / viral protein domain	Virus	None	α-Neu5Ac	1	0	0	1
Influenza hemagglutinin	β-sandwich / viral protein domain	Virus	None	α-Neu5Ac	5	23	19	47
Fiber knob	β-sandwich / virus globular domain	Virus	None	α-Neu5Ac	1	1	2	4
Fiber-knob parvovirus	β-sandwich / virus globular domain	Virus	None	α-Neu5Ac	10	4	0	14
Phage binding domain	β-sandwich / virus globular domain	Virus	None	β-GlcNAc	15	0	0	15
Turkey siadenovirus A	β-sandwich / virus globular domain	Virus	None	α-Neu5Ac	119	5	0	124
Amaranthin-like	β-trefoil	Plant	None	β-Gal	0	0	0	0
Boletus and Laetiporus β-trefoil lectin	β-trefoil	Fungal	None	β-Gal	83	16	0	99
Clitocybe lectin-like	β-trefoil	Fungal	None	β-Gal	2	8	0	10
Clostridial toxin	β-trefoil	Bacterial	43	β-Gal	258	45	55	358
Cys-rich man-receptor	β-trefoil	Animal	None	β-Gal	3	4	0	7
Earthworm lectin	β-trefoil	Animal	None	β-Gal	212	192	0	404
Fungi and Clostridium β-trefoil lectin	β-trefoil	Mixed	None	β-Gal	29	11	39	79
Mussel lectin	β-trefoil	Animal	None	α-Gal	9	32	0	41
Ricin-like	β-trefoil	Mixed	None	β-Gal	5140	9475	0	14615
Sclerotinia lectin like	β-trefoil	Fungal	None	β-Gal	1	3	0	4
Trefoil Factor	peptide	Animal	None	α-GlcNAc	4	4	3	11
SML2 Micronemal protein	small protein / APPLE domain	Animal	None	β-Gal	77	166	0	243
Cyanobacterial scytovirin	small protein / disulfide rich	Bacterial	3	NA	2	25	6	33
Invertebrate chitin-binding protein	small protein / Invertebrate chitin-binding protein	Animal	None	β-GlcNAc	0	21	0	21
Ginkbilobin	small protein / Knottin	Plant	None	α-Man	1	1	12	14
Ginkbilobin-like	small protein / Knottin	Fungal	None	NA	0	0	0	0
hevein	small protein / Knottin	Plant	None	β-GlcNAc	1	19	1	21
Lyophyllum ginkbilobin-like	small protein / Knottin	Fungal	None	α-Gal	2	0	0	2
Spider_toxin	small protein / Knottin	Animal	None	NA	0	0	0	0

Supplementary Table 2: List of the species and strains used in the study

Species and strain	Commensal/Pathobiont*	Source
Escherichia_coli_UMB0731	Pathobiont	PMID 29674608
Escherichia_coli_UMB0789	Pathobiont	PMID 29674608
Escherichia_coli_UMB0900	Pathobiont	PMID 29674608
Escherichia_coli_UMB0901	Pathobiont	PMID 29674608
Escherichia_coli_UMB6721	Pathobiont	Bioproject PRJNA316969
Escherichia_coli_UMB7431	Pathobiont	Bioproject PRJNA316969
Gardnerella_vaginalis_DSM4944	Pathobiont	DSMZ type collection ID 4944
Gardnerella_vaginalis_GED7275B	Pathobiont	PMID 30633889
Gardnerella_vaginalis_GED7760B	Pathobiont	PMID 30633889
Gardnerella_vaginalis_UMB0032A	Pathobiont	PMID 29674608
Gardnerella_vaginalis_UMB0032B	Pathobiont	PMID 29674608
Gardnerella_vaginalis_UMB0061	Pathobiont	PMID 29674608
Gardnerella_vaginalis_UMB0170	Pathobiont	Bioproject PRJNA316969
Gardnerella_vaginalis_UMB0233	Pathobiont	PMID 29674608
Gardnerella_vaginalis_UMB0264	Pathobiont	Bioproject PRJNA316969
Gardnerella_vaginalis_UMB0298	Pathobiont	PMID 29674608
Gardnerella_vaginalis_UMB0386	Pathobiont	PMID 29674608
Gardnerella_vaginalis_UMB0682	Pathobiont	PMID 29674608
Gardnerella_vaginalis_UMB0768	Pathobiont	Bioproject PRJNA316969
Gardnerella_vaginalis_UMB0770	Pathobiont	PMID 29674608
Gardnerella_vaginalis_UMB0775	Pathobiont	PMID 29674608
Gardnerella_vaginalis_UMB0830	Pathobiont	PMID 29674608
Gardnerella_vaginalis_UMB0833	Pathobiont	PMID 29674608
Gardnerella_vaginalis_UMB0912	Pathobiont	PMID 29674608
Gardnerella_vaginalis_UMB0913	Pathobiont	PMID 29674608
Lactobacillus_crispatus_C037	Commensal	Bioproject PRJNA316969
Lactobacillus_crispatus_MV-1A-US	Commensal	PMID 30633889
Lactobacillus_crispatus_SJ-3C-US	Commensal	PMID 30633889
Lactobacillus_crispatus_UMB0040	Commensal	Bioproject PRJNA316969
Lactobacillus_crispatus_UMB0044	Commensal	Bioproject PRJNA316969
Lactobacillus_crispatus_UMB0054	Commensal	PMID 29674608
Lactobacillus_crispatus_UMB0085	Commensal	PMID 29674608
Lactobacillus_crispatus_UMB0803	Commensal	PMID 29674608
Lactobacillus_crispatus_UMB0824	Commensal	PMID 29674608
Lactobacillus_crispatus_UMB1398	Commensal	PMID 29674608
Lactobacillus_crispatus_VMC3	Commensal	PMID 30633889
Lactobacillus_crispatus_VMC4	Commensal	PMID 30633889
Lactobacillus_crispatus_VMC5	Commensal	PMID 30633889
Lactobacillus_crispatus_VMC7	Commensal	PMID 30633889
Lactobacillus_crispatus_VMC8	Commensal	PMID 30633889
Lactobacillus_delbrueckii_UMB0003	Commensal	PMID 29674608
Lactobacillus_fermentum_UMB0187	Commensal	PMID 29674608
Lactobacillus_gasseri_202-4	Commensal	PMID 30633889
Lactobacillus_gasseri_SJ-9E-US	Commensal	PMID 30633889
Lactobacillus_gasseri_SV-16A-US	Commensal	PMID 30633889
Lactobacillus_gasseri_UMB0045	Commensal	PMID 29674608
Lactobacillus_gasseri_UMB0045b	Commensal	PMID 29674608
Lactobacillus_gasseri_UMB0099	Commensal	PMID 29674608
Lactobacillus_gasseri_UMB1399	Commensal	Bioproject PRJNA316969
Lactobacillus_iners_ATCC55195	Pathobiont	PMID 30633889
Lactobacillus_iners_DSM13335	Pathobiont	DSMZ type collection ID 13335
Lactobacillus_iners_LEAF2052A-d	Pathobiont	PMID 30633889
Lactobacillus_iners_SPIN2503V10-d	Pathobiont	PMID 30633889
Lactobacillus_iners_UMB0030	Pathobiont	Bioproject PRJNA316969
Lactobacillus_iners_UMB0033	Pathobiont	Bioproject PRJNA316969
Lactobacillus_iners_UMB1051	Pathobiont	Bioproject PRJNA316969
Lactobacillus_jensenii_115-3-CHN	Commensal	PMID 30633889
Lactobacillus_jensenii_269-3	Commensal	PMID 30633889
Lactobacillus_jensenii_SJ-7A-US	Commensal	PMID 30633889
Lactobacillus_jensenii_UMB0007	Commensal	PMID 29674608
Lactobacillus_jensenii_UMB0034	Commensal	Bioproject PRJNA316969
Lactobacillus_jensenii_UMB0037	Commensal	Bioproject PRJNA316969
Lactobacillus_jensenii_UMB0077	Commensal	PMID 29674608
Lactobacillus_jensenii_UMB1307	Commensal	Bioproject PRJNA316969
Lactobacillus_jensenii_UMB1355	Commensal	Bioproject PRJNA316969

Lactobacillus_rhamnosus_51B	Commensal	PMID 30633889
Lactobacillus_vaginalis_ATCC49540	Commensal	PMID 30633889
Prevotella_amnii_DNF00058	Pathobiont	PMID 30633889
Prevotella_amnii_DNF00307	Pathobiont	PMID 30633889
Prevotella_bivia_DNF00188	Pathobiont	PMID 30633889
Prevotella_bivia_DNF00320	Pathobiont	PMID 30633889
Prevotella_bivia_DNF00650	Pathobiont	PMID 30633889
Prevotella_bivia_GED7760C	Pathobiont	PMID 30633889
Prevotella_corporis_MJR7716	Pathobiont	PMID 30633889
Prevotella_denticola_DNF00960	Pathobiont	PMID 30633889
Prevotella_disiens_DNF00882	Pathobiont	PMID 30633889
Prevotella_timonensis_S9-PR14	Pathobiont	PMID 30633889
Streptococcus_agalactiae_UMB0049	Pathobiont	Bioproject PRJNA316969
Streptococcus_agalactiae_UMB0767	Pathobiont	Bioproject PRJNA316969
Streptococcus_agalactiae_UMB0776	Pathobiont	Bioproject PRJNA316969
Streptococcus_anginosus_UMB0252	Pathobiont	PMID 29674608
Streptococcus_anginosus_UMB0820	Pathobiont	PMID 29674608
Streptococcus_anginosus_UMB0839	Pathobiont	PMID 29674608
Streptococcus_sanguinis_SK36	Pathobiont	PMID: 31142849
Streptococcus_mitis_KCOM1350	Pathobiont	PMID: 31142849
Streptococcus_mitis_CMW7705B	Pathobiont	PMID: 30633889
Streptococcus_mitis_UMB0079	Pathobiont	PMID 29674608
Streptococcus_mitis_UMB1341	Pathobiont	PMID 29674608
Streptococcus_parasanguinis_UMB0216	Pathobiont	PMID 29674608
Streptococcus_salivarius_UMB0051	Pathobiont	PMID 29674608

*See Methods for definition of Pathobiont/Commensal

Supplementary Table 3: CBMs of interest for the present study with associated glycan specificity. The lectin specificities for glycans were manually recovered using CAZY database (www.cazy.org/Carbohydrate-Binding-Modules.html).

	found in	binding specificity	monosacc	fold
CBM3	bacterial enzymes	mainly cellulose, rarely chitin	β-Glc	β-sandwich
CBM4	bacterial enzymes	xylan, β-1,3-glucan, β-1,3-1,4-glucan, β-1,6-glucan, amorphous but not crystalline cellulose	β-Glc	β-sandwich
CBM5	bacterial enzymes	chitin	β-GlcNAc	
CBM6	bacteria, fungi, horseshoe crab	mostly amorphous cellulose and β-1,4-xylan, rarely β-1,3-glucan, β-1,3-1,4-glucan, and β-1,4-glucan.	β-Glc	β-sandwich
CBM8	bacteria +dictyostelium	cellulose	β-Glc	
CBM9	bacterial xylanases	xylan, rarely cellulose	β-Xyl	β-sandwich
CBM11	bacteria	β-1,4-glucan and β-1,3-1,4-mixed linked glucans	β-Glc	
CBM12	bacteria, fungi	chitin	β-GlcNAc	
CBM13	all	xylan, Man, Gal, GalNAc	β-Xyl	β-trefoil
CBM15	bacteria	xylan, xylo-oligosaccharides	β-Xyl	β-sandwich
CBM16	bacteria	cellulose, glucomannan	β-Glc	β-sandwich
CBM17	bacteria	amorphous and derivatized cellulose, cello-oligosaccharides	β-Glc	
CBM20	all	starch	α-Glc	
CBM22	all	mainly xylan, rarely mixed β-1,3/β-1,4-glucans	β-Xyl	β-sandwich
CBM25	bacteria	starch	α-Glc	β-sandwich
CBM26	bacteria, fungi	starch	α-Glc	β-sandwich
CBM27	bacteria	mannan	β-Man	
CBM32	all	Gal, lactose, LacNAc, polygalacturonic acid	β-Gal	β-sandwich
CBM34	bacteria	granular starch		
CBM35	all	xylan, mannan, β-galactan, manno-oligosaccharides	β-Xyl	
CBM36		calcium-dependent binding of xylan, xylo-oligosaccharides	β-Xyl	β-sandwich
CBM37	bacteria	xylan, chitin, microcrystalline and phosphoric-acid swollen cellulose	β-Xyl	
CBM38	bacteria, fungi	inulin	β-Glc	
CBM40	bacteria, worm	sialic acid	α-NeuAc	
CBM41	bacteria, plants	α-glucans amylose, amylopectin, pullulan, and oligosaccharide fragments derived from these polysaccharides	α-Glc	
CBM42	bacteria, fungi	arabinofuranose	α-Ara	β-trefoil
CBM45	plants	starch	α-Glc	
CBM46	bacteria	cellulose	β-Glc	
CBM47	all	fucose	α-Fuc	β-sandwich
CBM48	all	glycogen	α-Glc	
CBM49	plants	crystalline cellulose	β-Glc	
CBM51	bacteria	Gal, blood group A/B-antigens	α-Gal	β-sandwich
CBM56	bacteria, virus	β-1,3-glucan	β-Glc	
CBM60	bacteria	xylan	β-Xyl	
CBM61	bacteria	β-1,4-galactan	β-Gal	
CBM63	bacteria, fungi	cellulose	β-Glc	
CBM64	bacteria	cellulose	β-Glc	
CBM65	bacteria	range of β-glucans/significant preference for xyloglucan	β-Glc	
CBM66	bacteria, fungi	fructans	α-Fru	
CBM67	bacteria, fungi	rhamnose	α-Rha	
CBM68	bacteria	maltotriose	α-Glc	
CBM70	bacteria	hyaluronan	β-GlcA	β-sandwich
CBM71	bacteria	lactose, LacNAc	β-Gal	
CBM72	bacteria	insoluble cellulose, β-1,3/1,4-mixed linked glucans, xylan, and β-mannan	β-Glc	
CBM73	bacteria	chitin	β-GlcNAc	
CBM76	bacteria	xyloglucan, glucomannan, barley β-glucan	β-Xyl	
CBM83	bacteria	starch	α-Glc	

Supplementary Table 4 : Accession number of all predicted lectins and CBMs in studied lectomes from vaginal bacteria

Accession Number	Lectin fold	Lectin class	HMM-score	Species and strain	Type
WP_000644691.1	α/β mixed LysM domain	LysM-like	27.8	Escherichia_coli_UMB0731	LEC
WP_001272558.1	α/β mixed LysM domain	LysM-like	25.4	Escherichia_coli_UMB0731	LEC
WP_000522415.1	α/β mixed LysM domain	LysM-like	21.7	Escherichia_coli_UMB0731	LEC
WP_001272592.1	α/β mixed LysM domain	LysM-like	18.4	Escherichia_coli_UMB0731	LEC
WP_000502509.1	β-prism II	Pyocin	59.7	Escherichia_coli_UMB0731	LEC
WP_001245994.1	β-sandwich pili and adhesins	bacterial adhesin CfaE	96.9	Escherichia_coli_UMB0731	LEC
WP_000832236.1	β-sandwich pili and adhesins	bacterial adhesin FimH-FlmH	333.0	Escherichia_coli_UMB0731	LEC
WP_000520590.1	β-sandwich pili and adhesins	bacterial adhesin FimH-FlmH	271.7	Escherichia_coli_UMB0731	LEC
WP_001234180.1	β-sandwich pili and adhesins	bacterial adhesin FimH-FlmH	65.9	Escherichia_coli_UMB0731	LEC
WP_101984911.1	β-sandwich pili and adhesins	bacterial adhesin PapG	93.9	Escherichia_coli_UMB0731	LEC
WP_000644685.1	α/β mixed LysM domain	LysM-like	27.8	Escherichia_coli_UMB0789	LEC
WP_001272558.1	α/β mixed LysM domain	LysM-like	25.4	Escherichia_coli_UMB0789	LEC
WP_000522415.1	α/β mixed LysM domain	LysM-like	21.7	Escherichia_coli_UMB0789	LEC
WP_001272582.1	α/β mixed LysM domain	LysM-like	18.8	Escherichia_coli_UMB0789	LEC
WP_000502506.1	β-prism II	Pyocin	57.8	Escherichia_coli_UMB0789	LEC
WP_001221834.1	β-sandwich Galactose-binding domain-like	1 Calcium lectin	19.9	Escherichia_coli_UMB0789	LEC
WP_001327331.1	β-sandwich pili and adhesins	bacterial adhesin CfaE	15.3	Escherichia_coli_UMB0789	LEC
WP_021533897.1	β-sandwich pili and adhesins	bacterial adhesin FimH-FlmH	330.4	Escherichia_coli_UMB0789	LEC
WP_101986803.1	β-sandwich pili and adhesins	bacterial adhesin FimH-FlmH	266.9	Escherichia_coli_UMB0789	LEC
WP_001234179.1	β-sandwich pili and adhesins	bacterial adhesin FimH-FlmH	59.8	Escherichia_coli_UMB0789	LEC
WP_000644685.1	α/β mixed LysM domain	LysM-like	27.8	Escherichia_coli_UMB0900	LEC
WP_001272558.1	α/β mixed LysM domain	LysM-like	25.4	Escherichia_coli_UMB0900	LEC
WP_001539402.1	α/β mixed LysM domain	LysM-like	21.7	Escherichia_coli_UMB0900	LEC
WP_001272592.1	α/β mixed LysM domain	LysM-like	18.4	Escherichia_coli_UMB0900	LEC
WP_001540706.1	β-prism II	Pyocin	55.8	Escherichia_coli_UMB0900	LEC
WP_001774145.1	β-sandwich pili and adhesins	bacterial adhesin FimH-FlmH	330.5	Escherichia_coli_UMB0900	LEC
WP_000520591.1	β-sandwich pili and adhesins	bacterial adhesin FimH-FlmH	272.4	Escherichia_coli_UMB0900	LEC
WP_000644691.1	α/β mixed LysM domain	LysM-like	27.8	Escherichia_coli_UMB0901	LEC
WP_001272558.1	α/β mixed LysM domain	LysM-like	25.4	Escherichia_coli_UMB0901	LEC
WP_000522415.1	α/β mixed LysM domain	LysM-like	21.7	Escherichia_coli_UMB0901	LEC
WP_001272592.1	α/β mixed LysM domain	LysM-like	18.4	Escherichia_coli_UMB0901	LEC
WP_000502509.1	β-prism II	Pyocin	59.7	Escherichia_coli_UMB0901	LEC
WP_001245994.1	β-sandwich pili and adhesins	bacterial adhesin CfaE	96.9	Escherichia_coli_UMB0901	LEC
WP_000832236.1	β-sandwich pili and adhesins	bacterial adhesin FimH-FlmH	333.0	Escherichia_coli_UMB0901	LEC
WP_000520590.1	β-sandwich pili and adhesins	bacterial adhesin FimH-FlmH	271.7	Escherichia_coli_UMB0901	LEC
WP_001234180.1	β-sandwich pili and adhesins	bacterial adhesin FimH-FlmH	65.9	Escherichia_coli_UMB0901	LEC
WP_000759062.1	β-sandwich pili and adhesins	bacterial adhesin PapG	114.8	Escherichia_coli_UMB0901	LEC
WP_000821052.1	β-sandwich pili and adhesins	bacterial adhesin UclD	453.9	Escherichia_coli_UMB0901	LEC
WP_000644685.1	α/β mixed LysM domain	LysM-like	27.8	Escherichia_coli_UMB6721	LEC
WP_001309712.1	α/β mixed LysM domain	LysM-like	26.9	Escherichia_coli_UMB6721	LEC
WP_000522413.1	α/β mixed LysM domain	LysM-like	19.6	Escherichia_coli_UMB6721	LEC
WP_001272592.1	α/β mixed LysM domain	LysM-like	18.4	Escherichia_coli_UMB6721	LEC
WP_000671707.1	β-helix	Variable Lymphocyte Receptor	30.9	Escherichia_coli_UMB6721	LEC
WP_001309452.1	β-helix	Variable Lymphocyte Receptor	15.3	Escherichia_coli_UMB6721	LEC
WP_000832247.1	β-sandwich pili and adhesins	bacterial adhesin FimH-FlmH	335.2	Escherichia_coli_UMB6721	LEC
WP_000520696.1	β-sandwich pili and adhesins	bacterial adhesin FimH-FlmH	272.4	Escherichia_coli_UMB6721	LEC
WP_000758680.1	β-sandwich pili and adhesins	bacterial adhesin PapG	467.6	Escherichia_coli_UMB6721	LEC
WP_000004561.1	β-trefoil	Fungi and Clostridium β-trefoil lectin	15.6	Escherichia_coli_UMB6721	LEC
WP_000644685.1	α/β mixed LysM domain	LysM-like	27.8	Escherichia_coli_UMB7431	LEC
WP_001309712.1	α/β mixed LysM domain	LysM-like	26.9	Escherichia_coli_UMB7431	LEC
WP_000522413.1	α/β mixed LysM domain	LysM-like	19.6	Escherichia_coli_UMB7431	LEC
WP_001272592.1	α/β mixed LysM domain	LysM-like	18.4	Escherichia_coli_UMB7431	LEC
WP_000671707.1	β-helix	Variable Lymphocyte Receptor	30.9	Escherichia_coli_UMB7431	LEC
WP_001309452.1	β-helix	Variable Lymphocyte Receptor	15.3	Escherichia_coli_UMB7431	LEC
WP_000832247.1	β-sandwich pili and adhesins	bacterial adhesin FimH-FlmH	335.2	Escherichia_coli_UMB7431	LEC
WP_000520696.1	β-sandwich pili and adhesins	bacterial adhesin FimH-FlmH	272.4	Escherichia_coli_UMB7431	LEC
WP_000758680.1	β-sandwich pili and adhesins	bacterial adhesin PapG	467.6	Escherichia_coli_UMB7431	LEC
WP_000004561.1	β-trefoil	Fungi and Clostridium β-trefoil lectin	15.6	Escherichia_coli_UMB7431	LEC
WP_004112269.1	α/β mixed LysM domain	LysM-like	13.9	Gardnerella_vaginalis_DSM4944	LEC
WP_054448917.1	β-sandwich Ig-like	serine-rich repeat adhesin	26.7	Gardnerella_vaginalis_DSM4944	LEC
WP_004130081.1	α/β mixed LysM domain	LysM-like	15.1	Gardnerella_vaginalis_GED7275B	LEC
WP_060785480.1	α/β OB-fold	Thyphoid toxin like AB5	13.4	Gardnerella_vaginalis_GED7275B	LEC
WP_060785654.1	β-sandwich Ig-like	serine-rich repeat adhesin	14.5	Gardnerella_vaginalis_GED7275B	LEC
WP_060785770.1	β-trefoil	Coprinus β-trefoil lectin	15.5	Gardnerella_vaginalis_GED7275B	LEC
WP_060787275.1	α/β mixed LysM domain	LysM-like	13.9	Gardnerella_vaginalis_GED7760B	LEC
WP_060786878.1	α/β OB-fold	Cholera toxin like AB5	14.2	Gardnerella_vaginalis_GED7760B	LEC
WP_060786772.1	β-helix	Variable Lymphocyte Receptor	18.0	Gardnerella_vaginalis_GED7760B	LEC
WP_019261457.1	α/β mixed LysM domain	LysM-like	13.5	Gardnerella_vaginalis_UMB0032A	LEC
WP_101887880.1	β-sandwich Ig-like	Ig-like	16.5	Gardnerella_vaginalis_UMB0032A	LEC
WP_101887703.1	β-sandwich Ig-like	serine-rich repeat adhesin	15.7	Gardnerella_vaginalis_UMB0032A	LEC
WP_019261457.1	α/β mixed LysM domain	LysM-like	13.5	Gardnerella_vaginalis_UMB0032B	LEC
WP_101887880.1	β-sandwich Ig-like	Ig-like	16.5	Gardnerella_vaginalis_UMB0032B	LEC
WP_101887703.1	β-sandwich Ig-like	serine-rich repeat adhesin	15.7	Gardnerella_vaginalis_UMB0032B	LEC
WP_004112269.1	α/β mixed LysM domain	LysM-like	13.9	Gardnerella_vaginalis_UMB0061	LEC
WP_101888601.1	β-sandwich Ig-like	Ig-like	17.1	Gardnerella_vaginalis_UMB0061	LEC
WP_065189636.1	β-sandwich Ig-like	serine-rich repeat adhesin	19.6	Gardnerella_vaginalis_UMB0061	LEC
WP_064621970.1	β-sandwich Ig-like	serine-rich repeat adhesin	27.3	Gardnerella_vaginalis_UMB0170	LEC
WP_004112269.1	α/β mixed LysM domain	LysM-like	13.9	Gardnerella_vaginalis_UMB0233	LEC
WP_101892070.1	β-sandwich Ig-like	Ig-like	16.4	Gardnerella_vaginalis_UMB0233	LEC
WP_064623505.1	β-sandwich Ig-like	serine-rich repeat adhesin	19.2	Gardnerella_vaginalis_UMB0233	LEC
WP_102706240.1	β-sandwich Ig-like	serine-rich repeat adhesin	27.2	Gardnerella_vaginalis_UMB0264	LEC
WP_004112269.1	α/β mixed LysM domain	LysM-like	13.9	Gardnerella_vaginalis_UMB0298	LEC
WP_101886357.1	β-sandwich Ig-like	Ig-like	16.3	Gardnerella_vaginalis_UMB0298	LEC
WP_101886590.1	β-sandwich Ig-like	serine-rich repeat adhesin	16.6	Gardnerella_vaginalis_UMB0298	LEC

WP_004112269.1	α/β mixed LysM domain	LysM-like	13.9	Gardnerella_vaginalis_UMB0386	LEC
WP_101886357.1	β-sandwich Ig-like	Ig-like	16.3	Gardnerella_vaginalis_UMB0386	LEC
WP_101886590.1	β-sandwich Ig-like	serine-rich repeat adhesin	16.6	Gardnerella_vaginalis_UMB0386	LEC
WP_004119297.1	α/β mixed C-type lectin-like	C-type lectin	42.2	Gardnerella_vaginalis_UMB0682	LEC
WP_004112269.1	α/β mixed LysM domain	LysM-like	13.9	Gardnerella_vaginalis_UMB0768	LEC
WP_102720613.1	β-sandwich Ig-like	Ig-like	16.3	Gardnerella_vaginalis_UMB0768	LEC
WP_004138763.1	β-sandwich Ig-like	serine-rich repeat adhesin	19.6	Gardnerella_vaginalis_UMB0768	LEC
WP_004112269.1	α/β mixed LysM domain	LysM-like	13.9	Gardnerella_vaginalis_UMB0770	LEC
WP_101891855.1	β-sandwich Ig-like	Ig-like	16.4	Gardnerella_vaginalis_UMB0770	LEC
WP_101891752.1	β-sandwich Ig-like	serine-rich repeat adhesin	18.7	Gardnerella_vaginalis_UMB0770	LEC
WP_004112269.1	α/β mixed LysM domain	LysM-like	13.9	Gardnerella_vaginalis_UMB0775	LEC
WP_101896639.1	α/β mixed LysM domain	LysM-like	14.0	Gardnerella_vaginalis_UMB0830	LEC
WP_101896687.1	α/β OB-fold	Cholera toxin like AB5	13.9	Gardnerella_vaginalis_UMB0830	LEC
WP_101896522.1	α/β OB-fold	Thyphoid toxin like AB5	13.8	Gardnerella_vaginalis_UMB0830	LEC
WP_101896518.1	α/β OB-fold	Thyphoid toxin like AB5	13.2	Gardnerella_vaginalis_UMB0830	LEC
WP_101896497.1	β-sandwich Ig-like	serine-rich repeat adhesin	14.3	Gardnerella_vaginalis_UMB0830	LEC
WP_004132377.1	β-trefoil	Coprinus β-trefoil lectin	15.5	Gardnerella_vaginalis_UMB0830	LEC
WP_101889680.1	α/β mixed LysM domain	LysM-like	14.1	Gardnerella_vaginalis_UMB0833	LEC
WP_018645359.1	α/β OB-fold	Thyphoid toxin like AB5	13.3	Gardnerella_vaginalis_UMB0833	LEC
WP_101889768.1	β-sandwich Ig-like	serine-rich repeat adhesin	21.4	Gardnerella_vaginalis_UMB0833	LEC
WP_004131195.1	β-trefoil	Coprinus β-trefoil lectin	16.0	Gardnerella_vaginalis_UMB0833	LEC
WP_004119297.1	α/β mixed C-type lectin-like	C-type lectin	42.2	Gardnerella_vaginalis_UMB0912	LEC
WP_101886139.1	β-sandwich Ig-like	serine-rich repeat adhesin	24.8	Gardnerella_vaginalis_UMB0912	LEC
WP_004119297.1	α/β mixed C-type lectin-like	C-type lectin	42.2	Gardnerella_vaginalis_UMB0913	LEC
WP_101886139.1	β-sandwich Ig-like	serine-rich repeat adhesin	24.8	Gardnerella_vaginalis_UMB0913	LEC
WP_005725874.1	α/β mixed LysM domain	LysM-like	24.6	Lactobacillus_crispatus_C037	LEC
WP_068812980.1	α/β mixed LysM domain	LysM-like	20.0	Lactobacillus_crispatus_C037	LEC
WP_020993138.1	α/β mixed LysM domain	LysM-like	24.8	Lactobacillus_crispatus_MV-1A-US	LEC
WP_005722111.1	α/β mixed LysM domain	LysM-like	13.5	Lactobacillus_crispatus_MV-1A-US	LEC
WP_005725874.1	α/β mixed LysM domain	LysM-like	24.6	Lactobacillus_crispatus_SJ-3C-US	LEC
WP_035163944.1	β-sandwich ConA-like	galectin-like	12.6	Lactobacillus_crispatus_SJ-3C-US	LEC
WP_005725874.1	α/β mixed LysM domain	LysM-like	24.6	Lactobacillus_crispatus_UMB0040	LEC
WP_005725874.1	α/β mixed LysM domain	LysM-like	24.6	Lactobacillus_crispatus_UMB0044	LEC
WP_005719041.1	α/β mixed LysM domain	LysM-like	20.5	Lactobacillus_crispatus_UMB0044	LEC
WP_005725091.1	α/β mixed LysM domain	LysM-like	17.5	Lactobacillus_crispatus_UMB0044	LEC
WP_005719014.1	α/β mixed LysM domain	LysM-like	12.7	Lactobacillus_crispatus_UMB0044	LEC
WP_005725874.1	α/β mixed LysM domain	LysM-like	24.6	Lactobacillus_crispatus_UMB0054	LEC
WP_005719041.1	α/β mixed LysM domain	LysM-like	20.5	Lactobacillus_crispatus_UMB0054	LEC
WP_005719014.1	α/β mixed LysM domain	LysM-like	12.7	Lactobacillus_crispatus_UMB0054	LEC
WP_005725874.1	α/β mixed LysM domain	LysM-like	24.6	Lactobacillus_crispatus_UMB0085	LEC
WP_068812980.1	α/β mixed LysM domain	LysM-like	20.0	Lactobacillus_crispatus_UMB0085	LEC
WP_005725874.1	α/β mixed LysM domain	LysM-like	24.6	Lactobacillus_crispatus_UMB0803	LEC
WP_005725091.1	α/β mixed LysM domain	LysM-like	17.5	Lactobacillus_crispatus_UMB0803	LEC
WP_005725874.1	α/β mixed LysM domain	LysM-like	24.6	Lactobacillus_crispatus_UMB0824	LEC
WP_020993138.1	α/β mixed LysM domain	LysM-like	24.8	Lactobacillus_crispatus_UMB1398	LEC
WP_005719041.1	α/β mixed LysM domain	LysM-like	20.5	Lactobacillus_crispatus_UMB1398	LEC
WP_005725874.1	α/β mixed LysM domain	LysM-like	24.6	Lactobacillus_crispatus_VMC3	LEC
WP_128734178.1	α/β OB-fold	heat labile toxin IIB AB5	13.9	Lactobacillus_crispatus_VMC3	LEC
WP_020993138.1	α/β mixed LysM domain	LysM-like	24.8	Lactobacillus_crispatus_VMC4	LEC
WP_060463456.1	α/β mixed LysM domain	LysM-like	20.0	Lactobacillus_crispatus_VMC4	LEC
WP_060463493.1	α/β mixed LysM domain	LysM-like	16.9	Lactobacillus_crispatus_VMC4	LEC
WP_060463506.1	α/β mixed LysM domain	LysM-like	14.8	Lactobacillus_crispatus_VMC4	LEC
WP_005725874.1	α/β mixed LysM domain	LysM-like	24.6	Lactobacillus_crispatus_VMC5	LEC
WP_005728964.1	α/β mixed LysM domain	LysM-like	20.0	Lactobacillus_crispatus_VMC5	LEC
WP_005725091.1	α/β mixed LysM domain	LysM-like	17.5	Lactobacillus_crispatus_VMC5	LEC
WP_005724903.1	α/β mixed LysM domain	LysM-like	13.2	Lactobacillus_crispatus_VMC5	LEC
WP_005725874.1	α/β mixed LysM domain	LysM-like	24.6	Lactobacillus_crispatus_VMC7	LEC
WP_060462944.1	α/β mixed LysM domain	LysM-like	24.6	Lactobacillus_crispatus_VMC8	LEC
WP_005728964.1	α/β mixed LysM domain	LysM-like	20.0	Lactobacillus_crispatus_VMC8	LEC
WP_005725091.1	α/β mixed LysM domain	LysM-like	17.5	Lactobacillus_crispatus_VMC8	LEC
WP_060462857.1	α/β mixed LysM domain	LysM-like	16.1	Lactobacillus_crispatus_VMC8	LEC
WP_005724903.1	α/β mixed LysM domain	LysM-like	13.2	Lactobacillus_crispatus_VMC8	LEC
WP_013438958.1	α/β mixed LysM domain	LysM-like	23.6	Lactobacillus_delbrueckii_UMB0003	LEC
WP_013439398.1	α/β mixed LysM domain	LysM-like	12.5	Lactobacillus_delbrueckii_UMB0003	LEC
WP_101889000.1	α/β mixed LysM domain	LysM-like	139.0	Lactobacillus_fermentum_UMB0187	LEC
WP_101888759.1	α/β mixed LysM domain	LysM-like	65.0	Lactobacillus_fermentum_UMB0187	LEC
WP_014562247.1	α/β mixed LysM domain	LysM-like	24.7	Lactobacillus_fermentum_UMB0187	LEC
WP_100184171.1	α/β mixed LysM domain	LysM-like	23.4	Lactobacillus_fermentum_UMB0187	LEC
WP_101889135.1	α/β mixed LysM domain	LysM-like	21.5	Lactobacillus_fermentum_UMB0187	LEC
WP_101889171.1	α/β OB-fold	Shiga toxin AB5	12.8	Lactobacillus_fermentum_UMB0187	LEC
WP_100184107.1	β-propeller	PVL-like PropLec7B	12.7	Lactobacillus_fermentum_UMB0187	LEC
WP_076811407.1	β-sandwich Galactose-binding domain-like	1 Calcium lectin	15.7	Lactobacillus_fermentum_UMB0187	LEC
WP_003647408.1	α/β mixed LysM domain	LysM-like	15.1	Lactobacillus_gasseri_202-4	LEC
WP_003647734.1	α/β OB-fold	Staphylococcal Superantigen-Like	19.4	Lactobacillus_gasseri_202-4	LEC
WP_003647408.1	α/β mixed LysM domain	LysM-like	15.1	Lactobacillus_gasseri_SJ-9E-US	LEC
WP_003647734.1	α/β OB-fold	Staphylococcal Superantigen-Like	19.4	Lactobacillus_gasseri_SJ-9E-US	LEC
WP_003647408.1	α/β mixed LysM domain	LysM-like	15.1	Lactobacillus_gasseri_SV-16A-US	LEC
WP_003647734.1	α/β OB-fold	Staphylococcal Superantigen-Like	19.4	Lactobacillus_gasseri_SV-16A-US	LEC
WP_003647408.1	α/β mixed LysM domain	LysM-like	15.1	Lactobacillus_gasseri_UMB0045b	LEC
WP_003647734.1	α/β OB-fold	Staphylococcal Superantigen-Like	19.4	Lactobacillus_gasseri_UMB0045b	LEC
WP_003647408.1	α/β mixed LysM domain	LysM-like	15.1	Lactobacillus_gasseri_UMB0045	LEC
WP_003647734.1	α/β OB-fold	Staphylococcal Superantigen-Like	19.4	Lactobacillus_gasseri_UMB0045	LEC
WP_101890772.1	α/β mixed LysM domain	LysM-like	15.2	Lactobacillus_gasseri_UMB0099	LEC
WP_101890981.1	α/β OB-fold	Staphylococcal Superantigen-Like	19.4	Lactobacillus_gasseri_UMB0099	LEC
WP_035430074.1	β-propeller	Tachylectin-2-like PropLec5A	12.6	Lactobacillus_gasseri_UMB0099	LEC
WP_003649096.1	β-sandwich Ig-like	Ig-like	13.2	Lactobacillus_gasseri_UMB0099	LEC
TVV20494.1	α/β OB-fold	Staphylococcal Superantigen-Like	19.4	Lactobacillus_gasseri_UMB1399	LEC

WP_006730434.1	β-helix	E coli bacteriophage	10.5	Lactobacillus_iners_ATCC55195	LEC
WP_035511984.1	β-sandwich ConA-like	Laminin G-like	11.2	Lactobacillus_iners_ATCC55195	LEC
WP_035511933.1	β-sandwich Ig-like	serine-rich repeat adhesin	23.7	Lactobacillus_iners_ATCC55195	LEC
WP_006736913.1	β-sandwich pili and adhesins	bacterial adhesin PapG	13.8	Lactobacillus_iners_ATCC55195	LEC
WP_006729368.1	β-sandwich pili and adhesins	bacterial adhesin PsaA	12.1	Lactobacillus_iners_ATCC55195	LEC
WP_006728956.1	β-helix	E coli bacteriophage	10.5	Lactobacillus_iners_DSM13335	LEC
WP_006734094.1	β-sandwich ConA-like	Laminin G-like	11.7	Lactobacillus_iners_DSM13335	LEC
WP_035514790.1	β-sandwich Ig-like	serine-rich repeat adhesin	18.2	Lactobacillus_iners_DSM13335	LEC
WP_006729923.1	β-sandwich pili and adhesins	bacterial adhesin PapG	15.1	Lactobacillus_iners_DSM13335	LEC
WP_006729368.1	β-sandwich pili and adhesins	bacterial adhesin PsaA	12.1	Lactobacillus_iners_DSM13335	LEC
WP_006730434.1	β-helix	E coli bacteriophage	10.5	Lactobacillus_iners_LEAF2052A-d	LEC
WP_006735636.1	β-sandwich ConA-like	Laminin G-like	11.7	Lactobacillus_iners_LEAF2052A-d	LEC
WP_006735193.1	β-sandwich Ig-like	serine-rich repeat adhesin	24.9	Lactobacillus_iners_LEAF2052A-d	LEC
WP_006729368.1	β-sandwich pili and adhesins	bacterial adhesin PsaA	12.1	Lactobacillus_iners_LEAF2052A-d	LEC
WP_006731774.1	β-helix	E coli bacteriophage	10.5	Lactobacillus_iners_SPIN2503V10-d	LEC
WP_006734094.1	β-sandwich ConA-like	Laminin G-like	11.7	Lactobacillus_iners_SPIN2503V10-d	LEC
WP_006734387.1	β-sandwich Ig-like	serine-rich repeat adhesin	17.9	Lactobacillus_iners_SPIN2503V10-d	LEC
WP_006734509.1	β-sandwich pili and adhesins	bacterial adhesin PapG	14.9	Lactobacillus_iners_SPIN2503V10-d	LEC
WP_006729368.1	β-sandwich pili and adhesins	bacterial adhesin PsaA	12.1	Lactobacillus_iners_SPIN2503V10-d	LEC
WP_006728956.1	β-helix	E coli bacteriophage	10.5	Lactobacillus_iners_UMB0030	LEC
WP_102720440.1	β-sandwich ConA-like	Laminin G-like	11.9	Lactobacillus_iners_UMB0030	LEC
WP_006734509.1	β-sandwich pili and adhesins	bacterial adhesin PapG	14.9	Lactobacillus_iners_UMB0030	LEC
WP_006729368.1	β-sandwich pili and adhesins	bacterial adhesin PsaA	12.1	Lactobacillus_iners_UMB0030	LEC
WP_006734509.1	β-sandwich pili and adhesins	bacterial adhesin PapG	14.9	Lactobacillus_iners_UMB0033	LEC
WP_006729368.1	β-sandwich pili and adhesins	bacterial adhesin PsaA	12.1	Lactobacillus_iners_UMB0033	LEC
WP_102695533.1	β-helix	E coli bacteriophage	10.5	Lactobacillus_iners_UMB1051	LEC
WP_006735636.1	β-sandwich ConA-like	Laminin G-like	11.7	Lactobacillus_iners_UMB1051	LEC
WP_006737397.1	β-sandwich pili and adhesins	bacterial adhesin PapG	14.9	Lactobacillus_iners_UMB1051	LEC
WP_102695430.1	β-sandwich pili and adhesins	bacterial adhesin PsaA	12.1	Lactobacillus_iners_UMB1051	LEC
WP_006587239.1	α/β mixed LysM domain	LysM-like	19.8	Lactobacillus_jensenii_115-3-CHN	LEC
WP_006587522.1	β-propeller	AAL-like PropLec6A	19.4	Lactobacillus_jensenii_115-3-CHN	LEC
WP_006586280.1	β-sandwich PA14 adhesin	PA14 yeast adhesin	13.2	Lactobacillus_jensenii_115-3-CHN	LEC
WP_006584589.1	α/β mixed LysM domain	LysM-like	17.9	Lactobacillus_jensenii_269-3	LEC
WP_006584589.1	α/β mixed LysM domain	LysM-like	17.9	Lactobacillus_jensenii_SJ-7A-US	LEC
WP_006584589.1	α/β mixed LysM domain	LysM-like	17.9	Lactobacillus_jensenii_UMB0007	LEC
WP_006584589.1	α/β mixed LysM domain	LysM-like	17.9	Lactobacillus_jensenii_UMB0034	LEC
WP_006584589.1	α/β mixed LysM domain	LysM-like	17.9	Lactobacillus_jensenii_UMB0037	LEC
WP_006584589.1	α/β mixed LysM domain	LysM-like	17.9	Lactobacillus_jensenii_UMB0077	LEC
WP_006584589.1	α/β mixed LysM domain	LysM-like	17.9	Lactobacillus_jensenii_UMB1307	LEC
WP_006587522.1	β-propeller	AAL-like PropLec6A	19.4	Lactobacillus_jensenii_UMB1355	LEC
WP_006586280.1	β-sandwich PA14 adhesin	PA14 yeast adhesin	13.2	Lactobacillus_jensenii_UMB1355	LEC
WP_005689277.1	α/β mixed LysM domain	LysM-like	18.2	Lactobacillus_rhamnosus_51B	LEC
WP_005689215.1	β-helix	Shigella phage Sf6	11.6	Lactobacillus_rhamnosus_51B	LEC
WP_005684398.1	β-sandwich viral protein domain	Influenza hemagglutinin	13.0	Lactobacillus_rhamnosus_51B	LEC
WP_003716398.1	α/β mixed LysM domain	LysM-like	117.3	Lactobacillus_vaginalis_ATCC49540	LEC
WP_003717892.1	α/β mixed LysM domain	LysM-like	76.3	Lactobacillus_vaginalis_ATCC49540	LEC
WP_003716856.1	α/β mixed LysM domain	LysM-like	29.1	Lactobacillus_vaginalis_ATCC49540	LEC
WP_003717627.1	α/β mixed LysM domain	LysM-like	25.5	Lactobacillus_vaginalis_ATCC49540	LEC
WP_003717208.1	α/β mixed LysM domain	LysM-like	25.2	Lactobacillus_vaginalis_ATCC49540	LEC
WP_003717211.1	α/β mixed LysM domain	LysM-like	25.0	Lactobacillus_vaginalis_ATCC49540	LEC
WP_003716785.1	β-hairpin stack	Toxin repetitive domain	162.5	Lactobacillus_vaginalis_ATCC49540	LEC
WP_003716766.1	β-hairpin stack	Toxin repetitive domain	110.2	Lactobacillus_vaginalis_ATCC49540	LEC
WP_003716791.1	β-hairpin stack	Toxin repetitive domain	96.5	Lactobacillus_vaginalis_ATCC49540	LEC
WP_003716786.1	β-hairpin stack	Toxin repetitive domain	89.2	Lactobacillus_vaginalis_ATCC49540	LEC
WP_003716767.1	β-hairpin stack	Toxin repetitive domain	81.6	Lactobacillus_vaginalis_ATCC49540	LEC
WP_003716762.1	β-hairpin stack	Toxin repetitive domain	77.5	Lactobacillus_vaginalis_ATCC49540	LEC
WP_003716787.1	β-hairpin stack	Toxin repetitive domain	53.7	Lactobacillus_vaginalis_ATCC49540	LEC
WP_003717427.1	β-hairpin stack	Toxin repetitive domain	44.6	Lactobacillus_vaginalis_ATCC49540	LEC
WP_003718009.1	β-sandwich viral coat and capsid protein	Equine Rhinitis A Virus Coat Protein	13.6	Lactobacillus_vaginalis_ATCC49540	LEC
WP_003716705.1	β-sandwich viral protein domain	Influenza hemagglutinin	14.1	Lactobacillus_vaginalis_ATCC49540	LEC
WP_036855773.1	α/β barrel TIM	chi-lectin TIM	35.3	Prevotella_amnii_DNF00058	LEC
WP_036854181.1	α/β mixed LysM domain	LysM-like	15.6	Prevotella_amnii_DNF00058	LEC
WP_036856622.1	α/β OB-fold	Staphylococcal Superantigen-Like	13.9	Prevotella_amnii_DNF00058	LEC
WP_036856824.1	β-propeller	AAL-like PropLec6A	13.8	Prevotella_amnii_DNF00058	LEC
WP_036856564.1	β-sandwich Ig-like	Ig-like	17.3	Prevotella_amnii_DNF00058	LEC
WP_060933312.1	α/β barrel TIM	chi-lectin TIM	35.7	Prevotella_amnii_DNF00307	LEC
WP_060932952.1	α/β mixed LysM domain	LysM-like	14.8	Prevotella_amnii_DNF00307	LEC
WP_008451256.1	α/β OB-fold	Staphylococcal Superantigen-Like	13.9	Prevotella_amnii_DNF00307	LEC
WP_060932702.1	β-propeller	AAL-like PropLec6A	14.0	Prevotella_amnii_DNF00307	LEC
WP_155273697.1	α/β barrel TIM	chi-lectin TCLL	14.4	Prevotella_bivia_DNF00188	LEC
WP_004336437.1	β-sandwich cytolysin-like	Fungal fruit body lectin	15.9	Prevotella_bivia_DNF00188	LEC
WP_036887669.1	β-sandwich PA14 adhesin	PA14 RTX	15.5	Prevotella_bivia_DNF00188	LEC
WP_036864606.1	β-sandwich PA14 adhesin	PA14 RTX	14.0	Prevotella_bivia_DNF00188	LEC
WP_080696229.1	β-sandwich virus globular domain	Phage binding domain	13.2	Prevotella_bivia_DNF00188	LEC
WP_036866637.1	α/β OB-fold	Cholera toxin like AB5	15.0	Prevotella_bivia_DNF00320	LEC
WP_004336437.1	β-sandwich cytolysin-like	Fungal fruit body lectin	15.9	Prevotella_bivia_DNF00320	LEC
WP_036868300.1	β-sandwich PA14 adhesin	PA14 RTX	15.9	Prevotella_bivia_DNF00320	LEC
WP_155362340.1	α/β barrel TIM	chi-lectin TCLL	14.5	Prevotella_bivia_DNF00650	LEC
WP_036862683.1	β-sandwich cytolysin-like	Fungal fruit body lectin	16.0	Prevotella_bivia_DNF00650	LEC
WP_036863134.1	β-sandwich PA14 adhesin	PA14 RTX	18.7	Prevotella_bivia_DNF00650	LEC
WP_036864606.1	β-sandwich PA14 adhesin	PA14 RTX	14.0	Prevotella_bivia_DNF00650	LEC
WP_080696229.1	β-sandwich virus globular domain	Phage binding domain	13.2	Prevotella_bivia_DNF00650	LEC
WP_036863635.1	β-trefoil	Sclerotinia lectin like	12.9	Prevotella_bivia_DNF00650	LEC
WP_155273697.1	α/β barrel TIM	chi-lectin TCLL	14.4	Prevotella_bivia_GED7760C	LEC
WP_061315401.1	α/β barrel TIM	chi-lectin TCLL	12.4	Prevotella_bivia_GED7760C	LEC
WP_004336437.1	β-sandwich cytolysin-like	Fungal fruit body lectin	15.9	Prevotella_bivia_GED7760C	LEC
WP_061450520.1	β-sandwich PA14 adhesin	PA14 RTX	18.7	Prevotella_bivia_GED7760C	LEC

WP_036864606.1	β-sandwich PA14 adhesin	PA14 RTX	14.0	Prevotella_bivia_GED7760C	LEC
WP_060940877.1	β-helix	Variable Lymphocyte Receptor	15.4	Prevotella_corporis_MJR7716	LEC
WP_060940414.1	β-sandwich cytolysin-like	Fungal fruit body lectin	15.1	Prevotella_corporis_MJR7716	LEC
WP_080696229.1	β-sandwich virus globular domain	Phage binding domain	13.2	Prevotella_corporis_MJR7716	LEC
WP_036854185.1	α/β barrel TIM	chi-lectin TIM	39.5	Prevotella_denticola_DNF00960	LEC
WP_036853781.1	α/β hairpin non-globular proline-rich	P-domain of calnexin and reticulin	17.4	Prevotella_denticola_DNF00960	LEC
WP_036854009.1	β-helix	Variable Lymphocyte Receptor	17.7	Prevotella_denticola_DNF00960	LEC
WP_081940877.1	β-helix	Variable Lymphocyte Receptor	15.0	Prevotella_denticola_DNF00960	LEC
WP_036883693.1	α/β OB-fold	Thyphoid toxin like AB5	13.8	Prevotella_disiens_DNF00882	LEC
WP_036882341.1	β-helix	Variable Lymphocyte Receptor	20.7	Prevotella_disiens_DNF00882	LEC
WP_036882683.1	β-helix	Variable Lymphocyte Receptor	19.5	Prevotella_disiens_DNF00882	LEC
WP_036883578.1	β-helix	Variable Lymphocyte Receptor	16.9	Prevotella_disiens_DNF00882	LEC
WP_036882427.1	β-helix	Variable Lymphocyte Receptor	12.8	Prevotella_disiens_DNF00882	LEC
WP_156104755.1	α/β barrel TIM	chi-lectin TCLL	14.4	Prevotella_timonensis_S9-PR14	LEC
WP_036926111.1	α/β OB-fold	Cholera toxin like AB5	13.6	Prevotella_timonensis_S9-PR14	LEC
WP_036926608.1	β-helix	Variable Lymphocyte Receptor	19.7	Prevotella_timonensis_S9-PR14	LEC
WP_008126135.1	β-propeller	PVL-like PropLec7B	13.4	Prevotella_timonensis_S9-PR14	LEC
WP_008126094.1	β-propeller	PVL-like PropLec7B	13.0	Prevotella_timonensis_S9-PR14	LEC
WP_036926994.1	β-sandwich Galactose-binding domain-like	F-type lectin	13.5	Prevotella_timonensis_S9-PR14	LEC
WP_036926731.1	β-sandwich virus globular domain	Fiber knob	14.6	Prevotella_timonensis_S9-PR14	LEC
WP_036926172.1	small protein disulfide rich	Cyanobacterial scytovirin	14.3	Prevotella_timonensis_S9-PR14	LEC
WP_000143481.1	α/β mixed LysM domain	LysM-like	34.0	Streptococcus_agalactiae_UMB0049	LEC
WP_000029069.1	α/β mixed LysM domain	LysM-like	29.2	Streptococcus_agalactiae_UMB0049	LEC
WP_000783419.1	α/β mixed LysM domain	LysM-like	17.6	Streptococcus_agalactiae_UMB0049	LEC
WP_001286937.1	α/β mixed with β-sheet not classified	HOP-OMP adhesins	13.7	Streptococcus_agalactiae_UMB0049	LEC
WP_000029067.1	α/β mixed LysM domain	LysM-like	29.3	Streptococcus_agalactiae_UMB0767	LEC
WP_000783424.1	α/β mixed LysM domain	LysM-like	17.3	Streptococcus_agalactiae_UMB0767	LEC
WP_001286944.1	α/β mixed with β-sheet not classified	HOP-OMP adhesins	15.0	Streptococcus_agalactiae_UMB0767	LEC
WP_000029067.1	α/β mixed LysM domain	LysM-like	29.3	Streptococcus_agalactiae_UMB0776	LEC
WP_000783424.1	α/β mixed LysM domain	LysM-like	17.3	Streptococcus_agalactiae_UMB0776	LEC
WP_001286944.1	α/β mixed with β-sheet not classified	HOP-OMP adhesins	15.0	Streptococcus_agalactiae_UMB0776	LEC
WP_003031567.1	α/β mixed LysM domain	LysM-like	33.6	Streptococcus_anginosus_UMB0252	LEC
WP_070242161.1	α/β mixed LysM domain	LysM-like	26.0	Streptococcus_anginosus_UMB0252	LEC
WP_101749795.1	β-helix	Variable Lymphocyte Receptor	15.6	Streptococcus_anginosus_UMB0252	LEC
WP_000628972.1	β-sandwich ConA-like	ANXUR1 Malectin-like	11.6	Streptococcus_anginosus_UMB0252	LEC
WP_003033270.1	β-sandwich Galactose-binding domain-like	H-type lectin	13.4	Streptococcus_anginosus_UMB0252	LEC
WP_021001568.1	β-sandwich pili and adhesins	bacterial adhesin PsaA	15.6	Streptococcus_anginosus_UMB0252	LEC
WP_101800532.1	α/β mixed LysM domain	LysM-like	43.0	Streptococcus_anginosus_UMB0820	LEC
WP_003031567.1	α/β mixed LysM domain	LysM-like	33.6	Streptococcus_anginosus_UMB0820	LEC
WP_083307007.1	β-helix	Variable Lymphocyte Receptor	13.7	Streptococcus_anginosus_UMB0820	LEC
WP_024051801.1	β-sandwich pili and adhesins	bacterial adhesin PsaA	15.9	Streptococcus_anginosus_UMB0820	LEC
WP_101800944.1	α/β mixed LysM domain	LysM-like	43.6	Streptococcus_anginosus_UMB0839	LEC
WP_003031567.1	α/β mixed LysM domain	LysM-like	33.6	Streptococcus_anginosus_UMB0839	LEC
WP_017646700.1	α/β mixed LysM domain	LysM-like	12.6	Streptococcus_anginosus_UMB0839	LEC
WP_024053270.1	β-helix	Variable Lymphocyte Receptor	13.7	Streptococcus_anginosus_UMB0839	LEC
WP_024051801.1	β-sandwich pili and adhesins	bacterial adhesin PsaA	15.9	Streptococcus_anginosus_UMB0839	LEC
WP_060805907.1	α/β mixed LysM domain	LysM-like	22.1	Streptococcus_mitis_CMW7705B	LEC
WP_060806161.1	α/β mixed LysM domain	LysM-like	16.6	Streptococcus_mitis_CMW7705B	LEC
WP_155640150.1	α/β mixed LysM domain	LysM-like	13.9	Streptococcus_mitis_CMW7705B	LEC
WP_060805808.1	β-helix	Shigella phage Sf6	29.2	Streptococcus_mitis_CMW7705B	LEC
WP_081094117.1	β-sandwich Galactose-binding domain-like	F-type lectin	264.7	Streptococcus_mitis_CMW7705B	LEC
WP_081094120.1	β-sandwich Galactose-binding domain-like	F-type lectin	164.6	Streptococcus_mitis_CMW7705B	LEC
WP_000742895.1	β-sandwich pili and adhesins	bacterial adhesin PsaA	15.1	Streptococcus_mitis_CMW7705B	LEC
WP_060805181.1	β-sandwich pili and adhesins	bacterial adhesin UclD	12.2	Streptococcus_mitis_CMW7705B	LEC
WP_060805300.1	β-trefoil	Fungi and Clostridium β-trefoil lectin	12.9	Streptococcus_mitis_CMW7705B	LEC
WP_060627375.1	α/β mixed LysM domain	LysM-like	23.2	Streptococcus_mitis_KCOM1350	LEC
WP_060627224.1	α/β mixed LysM domain	LysM-like	15.4	Streptococcus_mitis_KCOM1350	LEC
WP_060627308.1	β-hairpin stack	Toxin repetitive domain	57.7	Streptococcus_mitis_KCOM1350	LEC
WP_049544004.1	β-hairpin stack	Toxin repetitive domain	55.7	Streptococcus_mitis_KCOM1350	LEC
WP_060627582.1	β-hairpin stack	Toxin repetitive domain	36.1	Streptococcus_mitis_KCOM1350	LEC
WP_060628486.1	β-hairpin stack	Toxin repetitive domain	30.5	Streptococcus_mitis_KCOM1350	LEC
WP_060627273.1	β-hairpin stack	Toxin repetitive domain	24.2	Streptococcus_mitis_KCOM1350	LEC
WP_060627605.1	β-hairpin stack	Toxin repetitive domain	22.4	Streptococcus_mitis_KCOM1350	LEC
WP_060627298.1	β-hairpin stack	Toxin repetitive domain	21.2	Streptococcus_mitis_KCOM1350	LEC
WP_060628696.1	β-hairpin stack	Toxin repetitive domain	20.9	Streptococcus_mitis_KCOM1350	LEC
WP_060627297.1	β-hairpin stack	Toxin repetitive domain	20.7	Streptococcus_mitis_KCOM1350	LEC
WP_060627300.1	β-hairpin stack	Toxin repetitive domain	20.4	Streptococcus_mitis_KCOM1350	LEC
WP_049544006.1	β-hairpin stack	Toxin repetitive domain	19.3	Streptococcus_mitis_KCOM1350	LEC
WP_060627603.1	β-hairpin stack	Toxin repetitive domain	18.8	Streptococcus_mitis_KCOM1350	LEC
WP_060627601.1	β-hairpin stack	Toxin repetitive domain	14.2	Streptococcus_mitis_KCOM1350	LEC
WP_060627273.1	β-sandwich 2 calcium lectin	2 calcium lectin	15.1	Streptococcus_mitis_KCOM1350	LEC
WP_060627326.1	β-sandwich ConA-like	Malectin	13.7	Streptococcus_mitis_KCOM1350	LEC
WP_060628580.1	β-sandwich pili and adhesins	bacterial adhesin PsaA	14.7	Streptococcus_mitis_KCOM1350	LEC
WP_101782075.1	α/β mixed LysM domain	LysM-like	21.8	Streptococcus_mitis_UMB0079	LEC
WP_101781600.1	β-hairpin stack	Toxin repetitive domain	39.7	Streptococcus_mitis_UMB0079	LEC
WP_101782752.1	β-hairpin stack	Toxin repetitive domain	35.0	Streptococcus_mitis_UMB0079	LEC
WP_101782751.1	β-hairpin stack	Toxin repetitive domain	32.9	Streptococcus_mitis_UMB0079	LEC
WP_101782683.1	β-hairpin stack	Toxin repetitive domain	13.3	Streptococcus_mitis_UMB0079	LEC
WP_000834742.1	β-helix	Shigella phage Sf6	29.2	Streptococcus_mitis_UMB0079	LEC
WP_101782438.1	β-sandwich ConA-like	Malectin	15.1	Streptococcus_mitis_UMB0079	LEC
WP_101782605.1	β-sandwich Galactose-binding domain-like	F-type lectin	160.9	Streptococcus_mitis_UMB0079	LEC
WP_101782162.1	β-sandwich pili and adhesins	bacterial adhesin PsaA	15.1	Streptococcus_mitis_UMB0079	LEC
WP_101782075.1	α/β mixed LysM domain	LysM-like	21.8	Streptococcus_mitis_UMB1341	LEC
WP_061759600.1	α/β mixed LysM domain	LysM-like	17.6	Streptococcus_mitis_UMB1341	LEC
WP_101785366.1	β-hairpin stack	Toxin repetitive domain	43.2	Streptococcus_mitis_UMB1341	LEC
WP_101782752.1	β-hairpin stack	Toxin repetitive domain	35.0	Streptococcus_mitis_UMB1341	LEC
WP_101782751.1	β-hairpin stack	Toxin repetitive domain	32.9	Streptococcus_mitis_UMB1341	LEC

WP_101786215.1	β-hairpin stack	Toxin repetitive domain	14.7	Streptococcus_mitis_UMB1341	LEC
WP_101786253.1	β-sandwich ConA-like	Malectin	15.0	Streptococcus_mitis_UMB1341	LEC
WP_101784689.1	β-sandwich Galactose-binding domain-like	F-type lectin	265.8	Streptococcus_mitis_UMB1341	LEC
WP_101784730.1	β-sandwich Galactose-binding domain-like	F-type lectin	161.4	Streptococcus_mitis_UMB1341	LEC
WP_000742895.1	β-sandwich pili and adhesins	bacterial adhesin PsaA	15.1	Streptococcus_mitis_UMB1341	LEC
WP_101785098.1	β-trefoil	Clitocybe lectin-like	13.5	Streptococcus_mitis_UMB1341	LEC
WP_049498743.1	α/β mixed LysM domain	LysM-like	30.4	Streptococcus_parasanguinis_UMB0216	LEC
WP_049499136.1	α/β mixed LysM domain	LysM-like	28.6	Streptococcus_parasanguinis_UMB0216	LEC
WP_049498329.1	α/β mixed LysM domain	LysM-like	21.7	Streptococcus_parasanguinis_UMB0216	LEC
WP_049504278.1	α/β OB-fold	Staphylococcal Superantigen-Like	13.4	Streptococcus_parasanguinis_UMB0216	LEC
WP_101770935.1	β-prism I	Vibrio β-prism	12.9	Streptococcus_parasanguinis_UMB0216	LEC
WP_101770741.1	β-sandwich pili and adhesins	bacterial adhesin PsaA	15.3	Streptococcus_parasanguinis_UMB0216	LEC
WP_101771075.1	small protein APPLE domain	SML2 Micronemal protein	16.2	Streptococcus_parasanguinis_UMB0216	LEC
WP_002892168.1	α/β mixed LysM domain	LysM-like	36.9	Streptococcus_salivarius_UMB0051	LEC
WP_064520593.1	α/β mixed LysM domain	LysM-like	26.6	Streptococcus_salivarius_UMB0051	LEC
WP_101799853.1	β-hairpin stack	Toxin repetitive domain	117.2	Streptococcus_salivarius_UMB0051	LEC
WP_064520775.1	β-hairpin stack	Toxin repetitive domain	108.1	Streptococcus_salivarius_UMB0051	LEC
WP_064520765.1	β-hairpin stack	Toxin repetitive domain	78.9	Streptococcus_salivarius_UMB0051	LEC
WP_064520761.1	β-hairpin stack	Toxin repetitive domain	73.3	Streptococcus_salivarius_UMB0051	LEC
WP_064520768.1	β-hairpin stack	Toxin repetitive domain	71.7	Streptococcus_salivarius_UMB0051	LEC
WP_101799858.1	β-hairpin stack	Toxin repetitive domain	68.5	Streptococcus_salivarius_UMB0051	LEC
WP_064520769.1	β-hairpin stack	Toxin repetitive domain	67.9	Streptococcus_salivarius_UMB0051	LEC
WP_064520548.1	β-hairpin stack	Toxin repetitive domain	64.9	Streptococcus_salivarius_UMB0051	LEC
WP_101799720.1	β-hairpin stack	Toxin repetitive domain	58.3	Streptococcus_salivarius_UMB0051	LEC
WP_014634753.1	β-hairpin stack	Toxin repetitive domain	58.2	Streptococcus_salivarius_UMB0051	LEC
WP_101799857.1	β-hairpin stack	Toxin repetitive domain	54.7	Streptococcus_salivarius_UMB0051	LEC
WP_080610693.1	β-hairpin stack	Toxin repetitive domain	54.3	Streptococcus_salivarius_UMB0051	LEC
WP_101799859.1	β-hairpin stack	Toxin repetitive domain	53.4	Streptococcus_salivarius_UMB0051	LEC
WP_064520766.1	β-hairpin stack	Toxin repetitive domain	51.8	Streptococcus_salivarius_UMB0051	LEC
WP_101799855.1	β-hairpin stack	Toxin repetitive domain	43.9	Streptococcus_salivarius_UMB0051	LEC
WP_014634764.1	β-hairpin stack	Toxin repetitive domain	42.8	Streptococcus_salivarius_UMB0051	LEC
WP_064520757.1	β-hairpin stack	Toxin repetitive domain	35.9	Streptococcus_salivarius_UMB0051	LEC
WP_002883853.1	β-hairpin stack	Toxin repetitive domain	32.8	Streptococcus_salivarius_UMB0051	LEC
WP_064521292.1	β-hairpin stack	Toxin repetitive domain	31.1	Streptococcus_salivarius_UMB0051	LEC
WP_011836324.1	α/β mixed LysM domain	LysM-like	33.7	Streptococcus_sanguinis_SK36	LEC
WP_002894257.1	α/β mixed LysM domain	LysM-like	32.7	Streptococcus_sanguinis_SK36	LEC
WP_011836875.1	α/β OB-fold	Pertussis toxin AB5	13.6	Streptococcus_sanguinis_SK36	LEC
WP_011836622.1	β-hairpin stack	Toxin repetitive domain	29.3	Streptococcus_sanguinis_SK36	LEC
WP_011836900.1	β-hairpin stack	Toxin repetitive domain	19.7	Streptococcus_sanguinis_SK36	LEC
WP_002895767.1	β-sandwich Galactose-binding domain-like	H-type lectin	14.9	Streptococcus_sanguinis_SK36	LEC
WP_011836739.1	β-sandwich Ig-like	serine-rich repeat adhesin	191.0	Streptococcus_sanguinis_SK36	LEC
WP_011836889.1	β-sandwich pili and adhesins	bacterial adhesin PsaA	16.2	Streptococcus_sanguinis_SK36	LEC
WP_000773146.1	CBM	CBM12	24.4	Escherichia_coli_UMB0731	CBM
WP_000059867.1	CBM	CBM22	17.4	Escherichia_coli_UMB0731	CBM
WP_021552752.1	CBM	CBM34	73.8	Escherichia_coli_UMB0731	CBM
WP_000557384.1	CBM	CBM36	15.9	Escherichia_coli_UMB0731	CBM
WP_001283717.1	CBM	CBM48	100.2	Escherichia_coli_UMB0731	CBM
WP_000192568.1	CBM	CBM48	67.5	Escherichia_coli_UMB0731	CBM
WP_001577284.1	CBM	CBM48	32.1	Escherichia_coli_UMB0731	CBM
WP_000644691.1	CBM	CBM50	105.7	Escherichia_coli_UMB0731	CBM
WP_001272558.1	CBM	CBM50	51.5	Escherichia_coli_UMB0731	CBM
WP_000522415.1	CBM	CBM50	49.5	Escherichia_coli_UMB0731	CBM
WP_001184045.1	CBM	CBM50	29.8	Escherichia_coli_UMB0731	CBM
WP_001272592.1	CBM	CBM50	27.6	Escherichia_coli_UMB0731	CBM
WP_021552816.1	CBM	CBM50	23.5	Escherichia_coli_UMB0731	CBM
WP_000598012.1	CBM	CBM50	22.2	Escherichia_coli_UMB0731	CBM
WP_000773146.1	CBM	CBM5	105.6	Escherichia_coli_UMB0731	CBM
WP_021552750.1	CBM	CBM67	22.2	Escherichia_coli_UMB0731	CBM
WP_101984944.1	CBM	CBM67	21.7	Escherichia_coli_UMB0731	CBM
WP_000773147.1	CBM	CBM12	24.4	Escherichia_coli_UMB0789	CBM
WP_001443069.1	CBM	CBM34	74.0	Escherichia_coli_UMB0789	CBM
WP_002430482.1	CBM	CBM34	15.2	Escherichia_coli_UMB0789	CBM
WP_000557384.1	CBM	CBM36	15.9	Escherichia_coli_UMB0789	CBM
WP_001283738.1	CBM	CBM48	100.3	Escherichia_coli_UMB0789	CBM
WP_000192578.1	CBM	CBM48	67.5	Escherichia_coli_UMB0789	CBM
WP_001346611.1	CBM	CBM48	32.1	Escherichia_coli_UMB0789	CBM
WP_000644685.1	CBM	CBM50	105.5	Escherichia_coli_UMB0789	CBM
WP_001272558.1	CBM	CBM50	51.5	Escherichia_coli_UMB0789	CBM
WP_000522415.1	CBM	CBM50	49.5	Escherichia_coli_UMB0789	CBM
WP_001184045.1	CBM	CBM50	29.8	Escherichia_coli_UMB0789	CBM
WP_001272582.1	CBM	CBM50	27.6	Escherichia_coli_UMB0789	CBM
WP_000817095.1	CBM	CBM50	23.5	Escherichia_coli_UMB0789	CBM
WP_016265943.1	CBM	CBM50	21.7	Escherichia_coli_UMB0789	CBM
WP_000773147.1	CBM	CBM5	105.6	Escherichia_coli_UMB0789	CBM
WP_000177874.1	CBM	CBM67	22.1	Escherichia_coli_UMB0789	CBM
WP_001082836.1	CBM	CBM67	21.7	Escherichia_coli_UMB0789	CBM
WP_000773146.1	CBM	CBM12	24.4	Escherichia_coli_UMB0900	CBM
WP_000874189.1	CBM	CBM21	14.9	Escherichia_coli_UMB0900	CBM
WP_032153858.1	CBM	CBM32	22.5	Escherichia_coli_UMB0900	CBM
WP_001538375.1	CBM	CBM34	74.5	Escherichia_coli_UMB0900	CBM
WP_000557384.1	CBM	CBM36	15.9	Escherichia_coli_UMB0900	CBM
WP_001283713.1	CBM	CBM48	100.3	Escherichia_coli_UMB0900	CBM
WP_001539975.1	CBM	CBM48	68.1	Escherichia_coli_UMB0900	CBM
WP_001774083.1	CBM	CBM48	32.1	Escherichia_coli_UMB0900	CBM
WP_000644685.1	CBM	CBM50	105.5	Escherichia_coli_UMB0900	CBM
WP_001272558.1	CBM	CBM50	51.5	Escherichia_coli_UMB0900	CBM
WP_001539402.1	CBM	CBM50	49.5	Escherichia_coli_UMB0900	CBM

WP_001184045.1	CBM	CBM50	29.8	Escherichia_coli_UMB0900	CBM
WP_001272592.1	CBM	CBM50	27.6	Escherichia_coli_UMB0900	CBM
WP_000598225.1	CBM	CBM50	21.9	Escherichia_coli_UMB0900	CBM
WP_001538802.1	CBM	CBM50	21.3	Escherichia_coli_UMB0900	CBM
WP_000773146.1	CBM	CBM5	105.6	Escherichia_coli_UMB0900	CBM
WP_001538356.1	CBM	CBM67	22.0	Escherichia_coli_UMB0900	CBM
WP_001082856.1	CBM	CBM67	21.7	Escherichia_coli_UMB0900	CBM
WP_000773146.1	CBM	CBM12	24.4	Escherichia_coli_UMB0901	CBM
WP_001142475.1	CBM	CBM12	13.9	Escherichia_coli_UMB0901	CBM
WP_000874189.1	CBM	CBM21	14.9	Escherichia_coli_UMB0901	CBM
WP_021527668.1	CBM	CBM22	17.0	Escherichia_coli_UMB0901	CBM
WP_000979377.1	CBM	CBM34	73.8	Escherichia_coli_UMB0901	CBM
WP_000557384.1	CBM	CBM36	15.9	Escherichia_coli_UMB0901	CBM
WP_001283717.1	CBM	CBM48	100.2	Escherichia_coli_UMB0901	CBM
WP_000192568.1	CBM	CBM48	67.5	Escherichia_coli_UMB0901	CBM
WP_000644691.1	CBM	CBM50	105.7	Escherichia_coli_UMB0901	CBM
WP_001272558.1	CBM	CBM50	51.5	Escherichia_coli_UMB0901	CBM
WP_000522415.1	CBM	CBM50	49.5	Escherichia_coli_UMB0901	CBM
WP_001184045.1	CBM	CBM50	29.8	Escherichia_coli_UMB0901	CBM
WP_001272592.1	CBM	CBM50	27.6	Escherichia_coli_UMB0901	CBM
WP_000817084.1	CBM	CBM50	23.5	Escherichia_coli_UMB0901	CBM
WP_000598012.1	CBM	CBM50	22.2	Escherichia_coli_UMB0901	CBM
WP_000773146.1	CBM	CBM5	105.6	Escherichia_coli_UMB0901	CBM
WP_000177876.1	CBM	CBM67	22.2	Escherichia_coli_UMB0901	CBM
WP_001082915.1	CBM	CBM67	21.7	Escherichia_coli_UMB0901	CBM
WP_000874189.1	CBM	CBM21	14.9	Escherichia_coli_UMB6721	CBM
WP_001305478.1	CBM	CBM34	73.5	Escherichia_coli_UMB6721	CBM
WP_000557378.1	CBM	CBM36	14.8	Escherichia_coli_UMB6721	CBM
WP_001283730.1	CBM	CBM48	100.0	Escherichia_coli_UMB6721	CBM
WP_000192508.1	CBM	CBM48	67.3	Escherichia_coli_UMB6721	CBM
WP_000644685.1	CBM	CBM50	105.5	Escherichia_coli_UMB6721	CBM
WP_001309712.1	CBM	CBM50	51.5	Escherichia_coli_UMB6721	CBM
WP_000522413.1	CBM	CBM50	49.5	Escherichia_coli_UMB6721	CBM
WP_001184045.1	CBM	CBM50	29.8	Escherichia_coli_UMB6721	CBM
WP_001272592.1	CBM	CBM50	27.6	Escherichia_coli_UMB6721	CBM
WP_000817111.1	CBM	CBM50	23.5	Escherichia_coli_UMB6721	CBM
WP_000598211.1	CBM	CBM50	21.8	Escherichia_coli_UMB6721	CBM
WP_000907441.1	CBM	CBM63	31.0	Escherichia_coli_UMB6721	CBM
WP_000177868.1	CBM	CBM67	22.1	Escherichia_coli_UMB6721	CBM
WP_001082832.1	CBM	CBM67	21.7	Escherichia_coli_UMB6721	CBM
WP_001305478.1	CBM	CBM34	73.5	Escherichia_coli_UMB7431	CBM
WP_000557378.1	CBM	CBM36	14.8	Escherichia_coli_UMB7431	CBM
WP_001283730.1	CBM	CBM48	100.0	Escherichia_coli_UMB7431	CBM
WP_000192508.1	CBM	CBM48	67.3	Escherichia_coli_UMB7431	CBM
WP_000644685.1	CBM	CBM50	105.5	Escherichia_coli_UMB7431	CBM
WP_001309712.1	CBM	CBM50	51.5	Escherichia_coli_UMB7431	CBM
WP_000522413.1	CBM	CBM50	49.5	Escherichia_coli_UMB7431	CBM
WP_001184045.1	CBM	CBM50	29.8	Escherichia_coli_UMB7431	CBM
WP_001272592.1	CBM	CBM50	27.6	Escherichia_coli_UMB7431	CBM
WP_000817111.1	CBM	CBM50	23.5	Escherichia_coli_UMB7431	CBM
WP_000598211.1	CBM	CBM50	21.8	Escherichia_coli_UMB7431	CBM
WP_000907441.1	CBM	CBM63	31.0	Escherichia_coli_UMB7431	CBM
WP_000177868.1	CBM	CBM67	22.1	Escherichia_coli_UMB7431	CBM
WP_001082832.1	CBM	CBM67	21.7	Escherichia_coli_UMB7431	CBM
WP_013399749.1	CBM	CBM25	74.4	Gardnerella_vaginalis_DSM4944	CBM
WP_013399749.1	CBM	CBM26	26.3	Gardnerella_vaginalis_DSM4944	CBM
WP_009994174.1	CBM	CBM34	79.3	Gardnerella_vaginalis_DSM4944	CBM
WP_013399749.1	CBM	CBM34	13.0	Gardnerella_vaginalis_DSM4944	CBM
WP_013399749.1	CBM	CBM41	136.3	Gardnerella_vaginalis_DSM4944	CBM
WP_013399655.1	CBM	CBM48	72.6	Gardnerella_vaginalis_DSM4944	CBM
WP_004118272.1	CBM	CBM48	68.8	Gardnerella_vaginalis_DSM4944	CBM
WP_013399749.1	CBM	CBM48	52.7	Gardnerella_vaginalis_DSM4944	CBM
WP_009994410.1	CBM	CBM48	39.1	Gardnerella_vaginalis_DSM4944	CBM
WP_004112269.1	CBM	CBM50	36.4	Gardnerella_vaginalis_DSM4944	CBM
WP_013399386.1	CBM	CBM66	17.2	Gardnerella_vaginalis_DSM4944	CBM
WP_009993823.1	CBM	CBM67	16.7	Gardnerella_vaginalis_DSM4944	CBM
WP_009993889.1	CBM	CBM67	15.2	Gardnerella_vaginalis_DSM4944	CBM
WP_013399569.1	CBM	CBM76	12.1	Gardnerella_vaginalis_DSM4944	CBM
WP_013399749.1	CBM	CBM83	20.1	Gardnerella_vaginalis_DSM4944	CBM
WP_060786022.1	CBM	CBM34	77.2	Gardnerella_vaginalis_GED7275B	CBM
WP_020757522.1	CBM	CBM48	72.7	Gardnerella_vaginalis_GED7275B	CBM
WP_032835868.1	CBM	CBM48	68.8	Gardnerella_vaginalis_GED7275B	CBM
WP_060785714.1	CBM	CBM48	37.5	Gardnerella_vaginalis_GED7275B	CBM
WP_004130081.1	CBM	CBM50	39.2	Gardnerella_vaginalis_GED7275B	CBM
WP_060785750.1	CBM	CBM66	16.1	Gardnerella_vaginalis_GED7275B	CBM
WP_060786790.1	CBM	CBM34	80.5	Gardnerella_vaginalis_GED7760B	CBM
WP_081090874.1	CBM	CBM48	72.5	Gardnerella_vaginalis_GED7760B	CBM
WP_060787358.1	CBM	CBM48	67.6	Gardnerella_vaginalis_GED7760B	CBM
WP_060786439.1	CBM	CBM48	37.8	Gardnerella_vaginalis_GED7760B	CBM
WP_060787275.1	CBM	CBM50	39.3	Gardnerella_vaginalis_GED7760B	CBM
WP_060786608.1	CBM	CBM66	18.8	Gardnerella_vaginalis_GED7760B	CBM
WP_101887704.1	CBM	CBM25	70.1	Gardnerella_vaginalis_UMB0032A	CBM
WP_101887704.1	CBM	CBM26	22.1	Gardnerella_vaginalis_UMB0032A	CBM
WP_101887954.1	CBM	CBM34	78.6	Gardnerella_vaginalis_UMB0032A	CBM
WP_101887704.1	CBM	CBM34	13.1	Gardnerella_vaginalis_UMB0032A	CBM
WP_101887704.1	CBM	CBM41	134.2	Gardnerella_vaginalis_UMB0032A	CBM
WP_036747199.1	CBM	CBM45	18.3	Gardnerella_vaginalis_UMB0032A	CBM

WP_004138229.1	CBM	CBM48	73.0	Gardnerella_vaginalis_UMB0032A	CBM
WP_004118272.1	CBM	CBM48	68.8	Gardnerella_vaginalis_UMB0032A	CBM
WP_101887704.1	CBM	CBM48	52.8	Gardnerella_vaginalis_UMB0032A	CBM
WP_101888036.1	CBM	CBM48	39.1	Gardnerella_vaginalis_UMB0032A	CBM
WP_019261457.1	CBM	CBM50	36.4	Gardnerella_vaginalis_UMB0032A	CBM
WP_101887786.1	CBM	CBM66	18.1	Gardnerella_vaginalis_UMB0032A	CBM
WP_101887711.1	CBM	CBM67	19.2	Gardnerella_vaginalis_UMB0032A	CBM
WP_101887763.1	CBM	CBM67	15.1	Gardnerella_vaginalis_UMB0032A	CBM
WP_101887704.1	CBM	CBM83	20.1	Gardnerella_vaginalis_UMB0032A	CBM
WP_101887704.1	CBM	CBM25	70.1	Gardnerella_vaginalis_UMB0032B	CBM
WP_101887704.1	CBM	CBM26	22.1	Gardnerella_vaginalis_UMB0032B	CBM
WP_101887954.1	CBM	CBM34	78.6	Gardnerella_vaginalis_UMB0032B	CBM
WP_101887704.1	CBM	CBM34	13.1	Gardnerella_vaginalis_UMB0032B	CBM
WP_101887704.1	CBM	CBM41	134.2	Gardnerella_vaginalis_UMB0032B	CBM
WP_036747199.1	CBM	CBM45	18.3	Gardnerella_vaginalis_UMB0032B	CBM
WP_004138229.1	CBM	CBM48	73.0	Gardnerella_vaginalis_UMB0032B	CBM
WP_004118272.1	CBM	CBM48	68.8	Gardnerella_vaginalis_UMB0032B	CBM
WP_101887704.1	CBM	CBM48	52.8	Gardnerella_vaginalis_UMB0032B	CBM
WP_101888036.1	CBM	CBM48	39.1	Gardnerella_vaginalis_UMB0032B	CBM
WP_019261457.1	CBM	CBM50	36.4	Gardnerella_vaginalis_UMB0032B	CBM
WP_101887786.1	CBM	CBM66	18.1	Gardnerella_vaginalis_UMB0032B	CBM
WP_101887711.1	CBM	CBM67	19.2	Gardnerella_vaginalis_UMB0032B	CBM
WP_101887763.1	CBM	CBM67	15.1	Gardnerella_vaginalis_UMB0032B	CBM
WP_101887704.1	CBM	CBM83	20.1	Gardnerella_vaginalis_UMB0032B	CBM
WP_081264702.1	CBM	CBM25	72.7	Gardnerella_vaginalis_UMB0061	CBM
WP_081264702.1	CBM	CBM26	24.1	Gardnerella_vaginalis_UMB0061	CBM
WP_065189315.1	CBM	CBM32	51.4	Gardnerella_vaginalis_UMB0061	CBM
WP_101888598.1	CBM	CBM32	34.4	Gardnerella_vaginalis_UMB0061	CBM
WP_065189466.1	CBM	CBM34	79.4	Gardnerella_vaginalis_UMB0061	CBM
WP_081264702.1	CBM	CBM41	136.0	Gardnerella_vaginalis_UMB0061	CBM
WP_081264748.1	CBM	CBM48	72.7	Gardnerella_vaginalis_UMB0061	CBM
WP_004118272.1	CBM	CBM48	68.8	Gardnerella_vaginalis_UMB0061	CBM
WP_081264702.1	CBM	CBM48	52.8	Gardnerella_vaginalis_UMB0061	CBM
WP_004138446.1	CBM	CBM48	39.1	Gardnerella_vaginalis_UMB0061	CBM
WP_004112269.1	CBM	CBM50	36.4	Gardnerella_vaginalis_UMB0061	CBM
WP_101888661.1	CBM	CBM66	16.0	Gardnerella_vaginalis_UMB0061	CBM
WP_064623052.1	CBM	CBM67	19.3	Gardnerella_vaginalis_UMB0061	CBM
WP_065189314.1	CBM	CBM67	15.1	Gardnerella_vaginalis_UMB0061	CBM
WP_081264702.1	CBM	CBM83	19.0	Gardnerella_vaginalis_UMB0061	CBM
WP_064621764.1	CBM	CBM25	55.2	Gardnerella_vaginalis_UMB0170	CBM
WP_064621764.1	CBM	CBM26	29.0	Gardnerella_vaginalis_UMB0170	CBM
WP_041160459.1	CBM	CBM27	13.8	Gardnerella_vaginalis_UMB0170	CBM
WP_064621764.1	CBM	CBM41	131.6	Gardnerella_vaginalis_UMB0170	CBM
WP_100066738.1	CBM	CBM48	77.2	Gardnerella_vaginalis_UMB0170	CBM
WP_064621526.1	CBM	CBM48	66.7	Gardnerella_vaginalis_UMB0170	CBM
WP_064621764.1	CBM	CBM48	53.9	Gardnerella_vaginalis_UMB0170	CBM
WP_004108138.1	CBM	CBM48	45.2	Gardnerella_vaginalis_UMB0170	CBM
WP_012914030.1	CBM	CBM50	33.9	Gardnerella_vaginalis_UMB0170	CBM
WP_064622025.1	CBM	CBM51	14.1	Gardnerella_vaginalis_UMB0170	CBM
WP_064621764.1	CBM	CBM83	30.4	Gardnerella_vaginalis_UMB0170	CBM
WP_081264607.1	CBM	CBM25	74.1	Gardnerella_vaginalis_UMB0233	CBM
WP_081264607.1	CBM	CBM26	26.5	Gardnerella_vaginalis_UMB0233	CBM
WP_032834382.1	CBM	CBM32	34.4	Gardnerella_vaginalis_UMB0233	CBM
WP_064622899.1	CBM	CBM34	80.6	Gardnerella_vaginalis_UMB0233	CBM
WP_081264607.1	CBM	CBM41	136.3	Gardnerella_vaginalis_UMB0233	CBM
WP_004138229.1	CBM	CBM48	73.0	Gardnerella_vaginalis_UMB0233	CBM
WP_004118272.1	CBM	CBM48	68.8	Gardnerella_vaginalis_UMB0233	CBM
WP_081264607.1	CBM	CBM48	52.9	Gardnerella_vaginalis_UMB0233	CBM
WP_064623100.1	CBM	CBM48	39.1	Gardnerella_vaginalis_UMB0233	CBM
WP_004112269.1	CBM	CBM50	36.4	Gardnerella_vaginalis_UMB0233	CBM
WP_064623276.1	CBM	CBM66	18.2	Gardnerella_vaginalis_UMB0233	CBM
WP_064623052.1	CBM	CBM67	19.3	Gardnerella_vaginalis_UMB0233	CBM
WP_101892064.1	CBM	CBM67	15.1	Gardnerella_vaginalis_UMB0233	CBM
WP_081264607.1	CBM	CBM83	20.1	Gardnerella_vaginalis_UMB0233	CBM
WP_064621764.1	CBM	CBM25	55.2	Gardnerella_vaginalis_UMB0264	CBM
WP_064621764.1	CBM	CBM26	29.0	Gardnerella_vaginalis_UMB0264	CBM
WP_041160459.1	CBM	CBM27	13.8	Gardnerella_vaginalis_UMB0264	CBM
WP_064621764.1	CBM	CBM41	131.6	Gardnerella_vaginalis_UMB0264	CBM
WP_100066738.1	CBM	CBM48	77.2	Gardnerella_vaginalis_UMB0264	CBM
WP_064621526.1	CBM	CBM48	66.7	Gardnerella_vaginalis_UMB0264	CBM
WP_064621764.1	CBM	CBM48	53.9	Gardnerella_vaginalis_UMB0264	CBM
WP_004108138.1	CBM	CBM48	45.2	Gardnerella_vaginalis_UMB0264	CBM
WP_012914030.1	CBM	CBM50	33.9	Gardnerella_vaginalis_UMB0264	CBM
WP_064622025.1	CBM	CBM51	14.1	Gardnerella_vaginalis_UMB0264	CBM
WP_064621764.1	CBM	CBM83	30.4	Gardnerella_vaginalis_UMB0264	CBM
WP_101886591.1	CBM	CBM25	74.6	Gardnerella_vaginalis_UMB0298	CBM
WP_101886591.1	CBM	CBM26	26.3	Gardnerella_vaginalis_UMB0298	CBM
WP_101886661.1	CBM	CBM32	51.5	Gardnerella_vaginalis_UMB0298	CBM
WP_004112247.1	CBM	CBM32	34.4	Gardnerella_vaginalis_UMB0298	CBM
WP_004117717.1	CBM	CBM34	79.3	Gardnerella_vaginalis_UMB0298	CBM
WP_101886591.1	CBM	CBM34	13.2	Gardnerella_vaginalis_UMB0298	CBM
WP_101886591.1	CBM	CBM41	136.4	Gardnerella_vaginalis_UMB0298	CBM
WP_004138229.1	CBM	CBM48	73.0	Gardnerella_vaginalis_UMB0298	CBM
WP_004112514.1	CBM	CBM48	68.8	Gardnerella_vaginalis_UMB0298	CBM
WP_101886591.1	CBM	CBM48	52.7	Gardnerella_vaginalis_UMB0298	CBM
WP_004138446.1	CBM	CBM48	39.1	Gardnerella_vaginalis_UMB0298	CBM
WP_004112269.1	CBM	CBM50	36.4	Gardnerella_vaginalis_UMB0298	CBM

WP_101886676.1	CBM	CBM66	15.9	Gardnerella_vaginalis_UMB0298	CBM
WP_101886608.1	CBM	CBM67	16.7	Gardnerella_vaginalis_UMB0298	CBM
WP_101886660.1	CBM	CBM67	15.0	Gardnerella_vaginalis_UMB0298	CBM
WP_101886399.1	CBM	CBM76	12.1	Gardnerella_vaginalis_UMB0298	CBM
WP_101886591.1	CBM	CBM83	20.1	Gardnerella_vaginalis_UMB0298	CBM
WP_101886591.1	CBM	CBM25	74.6	Gardnerella_vaginalis_UMB0386	CBM
WP_101886591.1	CBM	CBM26	26.3	Gardnerella_vaginalis_UMB0386	CBM
WP_101886661.1	CBM	CBM32	51.5	Gardnerella_vaginalis_UMB0386	CBM
WP_004112247.1	CBM	CBM32	34.4	Gardnerella_vaginalis_UMB0386	CBM
WP_004117717.1	CBM	CBM34	79.3	Gardnerella_vaginalis_UMB0386	CBM
WP_101886591.1	CBM	CBM34	13.2	Gardnerella_vaginalis_UMB0386	CBM
WP_101886591.1	CBM	CBM41	136.4	Gardnerella_vaginalis_UMB0386	CBM
WP_004138229.1	CBM	CBM48	73.0	Gardnerella_vaginalis_UMB0386	CBM
WP_004112514.1	CBM	CBM48	68.8	Gardnerella_vaginalis_UMB0386	CBM
WP_101886591.1	CBM	CBM48	52.7	Gardnerella_vaginalis_UMB0386	CBM
WP_004138446.1	CBM	CBM48	39.1	Gardnerella_vaginalis_UMB0386	CBM
WP_004112269.1	CBM	CBM50	36.4	Gardnerella_vaginalis_UMB0386	CBM
WP_101886676.1	CBM	CBM66	15.9	Gardnerella_vaginalis_UMB0386	CBM
WP_101886608.1	CBM	CBM67	16.7	Gardnerella_vaginalis_UMB0386	CBM
WP_101886660.1	CBM	CBM67	15.0	Gardnerella_vaginalis_UMB0386	CBM
WP_101886399.1	CBM	CBM76	12.1	Gardnerella_vaginalis_UMB0386	CBM
WP_101886591.1	CBM	CBM83	20.1	Gardnerella_vaginalis_UMB0386	CBM
WP_064340199.1	CBM	CBM25	56.6	Gardnerella_vaginalis_UMB0682	CBM
WP_064340199.1	CBM	CBM41	120.2	Gardnerella_vaginalis_UMB0682	CBM
WP_101885991.1	CBM	CBM48	77.2	Gardnerella_vaginalis_UMB0682	CBM
WP_032835386.1	CBM	CBM48	66.7	Gardnerella_vaginalis_UMB0682	CBM
WP_064340199.1	CBM	CBM48	58.0	Gardnerella_vaginalis_UMB0682	CBM
WP_064340167.1	CBM	CBM48	46.1	Gardnerella_vaginalis_UMB0682	CBM
WP_064340256.1	CBM	CBM50	33.8	Gardnerella_vaginalis_UMB0682	CBM
WP_064340199.1	CBM	CBM83	21.6	Gardnerella_vaginalis_UMB0682	CBM
WP_102720811.1	CBM	CBM25	74.5	Gardnerella_vaginalis_UMB0768	CBM
WP_102720811.1	CBM	CBM26	26.3	Gardnerella_vaginalis_UMB0768	CBM
WP_102720880.1	CBM	CBM32	51.5	Gardnerella_vaginalis_UMB0768	CBM
WP_032834382.1	CBM	CBM32	34.4	Gardnerella_vaginalis_UMB0768	CBM
WP_004113514.1	CBM	CBM34	79.3	Gardnerella_vaginalis_UMB0768	CBM
WP_102720811.1	CBM	CBM41	136.4	Gardnerella_vaginalis_UMB0768	CBM
WP_004138229.1	CBM	CBM48	73.0	Gardnerella_vaginalis_UMB0768	CBM
WP_102720641.1	CBM	CBM48	68.8	Gardnerella_vaginalis_UMB0768	CBM
WP_102720811.1	CBM	CBM48	52.9	Gardnerella_vaginalis_UMB0768	CBM
WP_102720966.1	CBM	CBM48	39.1	Gardnerella_vaginalis_UMB0768	CBM
WP_004112269.1	CBM	CBM50	36.4	Gardnerella_vaginalis_UMB0768	CBM
WP_102720896.1	CBM	CBM66	16.0	Gardnerella_vaginalis_UMB0768	CBM
WP_102720821.1	CBM	CBM67	19.3	Gardnerella_vaginalis_UMB0768	CBM
WP_102720879.1	CBM	CBM67	15.0	Gardnerella_vaginalis_UMB0768	CBM
WP_102720811.1	CBM	CBM83	20.1	Gardnerella_vaginalis_UMB0768	CBM
WP_101891751.1	CBM	CBM25	71.0	Gardnerella_vaginalis_UMB0770	CBM
WP_101891751.1	CBM	CBM26	25.3	Gardnerella_vaginalis_UMB0770	CBM
WP_101886661.1	CBM	CBM32	51.5	Gardnerella_vaginalis_UMB0770	CBM
WP_101891957.1	CBM	CBM34	79.4	Gardnerella_vaginalis_UMB0770	CBM
WP_101891751.1	CBM	CBM34	13.1	Gardnerella_vaginalis_UMB0770	CBM
WP_101891751.1	CBM	CBM41	132.0	Gardnerella_vaginalis_UMB0770	CBM
WP_101891944.1	CBM	CBM48	73.0	Gardnerella_vaginalis_UMB0770	CBM
WP_004118272.1	CBM	CBM48	68.8	Gardnerella_vaginalis_UMB0770	CBM
WP_101891751.1	CBM	CBM48	52.7	Gardnerella_vaginalis_UMB0770	CBM
WP_101892005.1	CBM	CBM48	39.1	Gardnerella_vaginalis_UMB0770	CBM
WP_004112269.1	CBM	CBM50	36.4	Gardnerella_vaginalis_UMB0770	CBM
WP_101891675.1	CBM	CBM66	17.3	Gardnerella_vaginalis_UMB0770	CBM
WP_101887711.1	CBM	CBM67	19.2	Gardnerella_vaginalis_UMB0770	CBM
WP_101891697.1	CBM	CBM67	15.1	Gardnerella_vaginalis_UMB0770	CBM
WP_013399569.1	CBM	CBM76	12.1	Gardnerella_vaginalis_UMB0770	CBM
WP_101891751.1	CBM	CBM83	20.1	Gardnerella_vaginalis_UMB0770	CBM
WP_101889970.1	CBM	CBM32	51.0	Gardnerella_vaginalis_UMB0775	CBM
WP_004138265.1	CBM	CBM34	80.7	Gardnerella_vaginalis_UMB0775	CBM
WP_004138229.1	CBM	CBM48	73.0	Gardnerella_vaginalis_UMB0775	CBM
WP_004118272.1	CBM	CBM48	68.8	Gardnerella_vaginalis_UMB0775	CBM
WP_101890405.1	CBM	CBM48	39.1	Gardnerella_vaginalis_UMB0775	CBM
WP_004112269.1	CBM	CBM50	36.4	Gardnerella_vaginalis_UMB0775	CBM
WP_004137655.1	CBM	CBM66	18.1	Gardnerella_vaginalis_UMB0775	CBM
WP_101890020.1	CBM	CBM67	19.3	Gardnerella_vaginalis_UMB0775	CBM
WP_101889971.1	CBM	CBM67	15.1	Gardnerella_vaginalis_UMB0775	CBM
WP_101896601.1	CBM	CBM34	78.3	Gardnerella_vaginalis_UMB0830	CBM
WP_020757522.1	CBM	CBM48	72.7	Gardnerella_vaginalis_UMB0830	CBM
WP_018633810.1	CBM	CBM48	68.3	Gardnerella_vaginalis_UMB0830	CBM
WP_101896692.1	CBM	CBM48	41.4	Gardnerella_vaginalis_UMB0830	CBM
WP_101896639.1	CBM	CBM50	39.4	Gardnerella_vaginalis_UMB0830	CBM
WP_101896440.1	CBM	CBM66	15.9	Gardnerella_vaginalis_UMB0830	CBM
WP_101889252.1	CBM	CBM34	77.7	Gardnerella_vaginalis_UMB0833	CBM
WP_101889414.1	CBM	CBM48	72.3	Gardnerella_vaginalis_UMB0833	CBM
WP_032835868.1	CBM	CBM48	68.8	Gardnerella_vaginalis_UMB0833	CBM
WP_101889832.1	CBM	CBM48	38.6	Gardnerella_vaginalis_UMB0833	CBM
WP_101889680.1	CBM	CBM50	39.3	Gardnerella_vaginalis_UMB0833	CBM
WP_101889453.1	CBM	CBM66	16.0	Gardnerella_vaginalis_UMB0833	CBM
WP_101886184.1	CBM	CBM25	55.2	Gardnerella_vaginalis_UMB0912	CBM
WP_101886184.1	CBM	CBM26	28.7	Gardnerella_vaginalis_UMB0912	CBM
WP_101886184.1	CBM	CBM41	134.9	Gardnerella_vaginalis_UMB0912	CBM
WP_101885991.1	CBM	CBM48	77.2	Gardnerella_vaginalis_UMB0912	CBM
WP_032835386.1	CBM	CBM48	66.7	Gardnerella_vaginalis_UMB0912	CBM

WP_101886184.1	CBM	CBM48	52.5	Gardnerella_vaginalis_UMB0912	CBM
WP_101886208.1	CBM	CBM48	45.2	Gardnerella_vaginalis_UMB0912	CBM
WP_064340256.1	CBM	CBM50	33.8	Gardnerella_vaginalis_UMB0912	CBM
WP_101886184.1	CBM	CBM83	30.3	Gardnerella_vaginalis_UMB0912	CBM
WP_101886184.1	CBM	CBM25	55.2	Gardnerella_vaginalis_UMB0913	CBM
WP_101886184.1	CBM	CBM26	28.7	Gardnerella_vaginalis_UMB0913	CBM
WP_101886184.1	CBM	CBM41	134.9	Gardnerella_vaginalis_UMB0913	CBM
WP_101885991.1	CBM	CBM48	77.2	Gardnerella_vaginalis_UMB0913	CBM
WP_032835386.1	CBM	CBM48	66.7	Gardnerella_vaginalis_UMB0913	CBM
WP_101886184.1	CBM	CBM48	52.5	Gardnerella_vaginalis_UMB0913	CBM
WP_101886208.1	CBM	CBM48	45.2	Gardnerella_vaginalis_UMB0913	CBM
WP_064340256.1	CBM	CBM50	33.8	Gardnerella_vaginalis_UMB0913	CBM
WP_101886184.1	CBM	CBM83	30.3	Gardnerella_vaginalis_UMB0913	CBM
WP_068813721.1	CBM	CBM11	15.4	Lactobacillus_crispatus_C037	CBM
WP_013085889.1	CBM	CBM12	12.9	Lactobacillus_crispatus_C037	CBM
WP_068813884.1	CBM	CBM17	12.8	Lactobacillus_crispatus_C037	CBM
WP_068813412.1	CBM	CBM32	24.2	Lactobacillus_crispatus_C037	CBM
WP_068813604.1	CBM	CBM34	83.2	Lactobacillus_crispatus_C037	CBM
WP_005724064.1	CBM	CBM37	16.2	Lactobacillus_crispatus_C037	CBM
WP_005719486.1	CBM	CBM41	44.0	Lactobacillus_crispatus_C037	CBM
WP_005719486.1	CBM	CBM48	44.2	Lactobacillus_crispatus_C037	CBM
WP_005725874.1	CBM	CBM50	43.8	Lactobacillus_crispatus_C037	CBM
WP_068812980.1	CBM	CBM50	40.0	Lactobacillus_crispatus_C037	CBM
WP_068813001.1	CBM	CBM50	23.0	Lactobacillus_crispatus_C037	CBM
WP_068813882.1	CBM	CBM51	15.4	Lactobacillus_crispatus_C037	CBM
WP_005721518.1	CBM	CBM5	12.7	Lactobacillus_crispatus_C037	CBM
WP_068813676.1	CBM	CBM6	14.1	Lactobacillus_crispatus_C037	CBM
WP_005728417.1	CBM	CBM65	13.8	Lactobacillus_crispatus_C037	CBM
WP_005720567.1	CBM	CBM66	36.2	Lactobacillus_crispatus_C037	CBM
WP_013086596.1	CBM	CBM67	15.3	Lactobacillus_crispatus_C037	CBM
WP_005720501.1	CBM	CBM12	13.5	Lactobacillus_crispatus_MV-1A-US	CBM
WP_005721705.1	CBM	CBM32	23.2	Lactobacillus_crispatus_MV-1A-US	CBM
WP_005720049.1	CBM	CBM34	84.5	Lactobacillus_crispatus_MV-1A-US	CBM
WP_005718438.1	CBM	CBM37	16.2	Lactobacillus_crispatus_MV-1A-US	CBM
WP_005719486.1	CBM	CBM41	44.0	Lactobacillus_crispatus_MV-1A-US	CBM
WP_005719486.1	CBM	CBM48	44.2	Lactobacillus_crispatus_MV-1A-US	CBM
WP_020993138.1	CBM	CBM50	43.8	Lactobacillus_crispatus_MV-1A-US	CBM
WP_005722097.1	CBM	CBM50	38.3	Lactobacillus_crispatus_MV-1A-US	CBM
WP_005722111.1	CBM	CBM50	34.4	Lactobacillus_crispatus_MV-1A-US	CBM
WP_005719393.1	CBM	CBM51	15.2	Lactobacillus_crispatus_MV-1A-US	CBM
WP_005722537.1	CBM	CBM5	13.3	Lactobacillus_crispatus_MV-1A-US	CBM
WP_005720567.1	CBM	CBM66	36.2	Lactobacillus_crispatus_MV-1A-US	CBM
WP_005719548.1	CBM	CBM67	15.7	Lactobacillus_crispatus_MV-1A-US	CBM
WP_005727913.1	CBM	CBM12	15.3	Lactobacillus_crispatus_SJ-3C-US	CBM
WP_035163454.1	CBM	CBM32	22.6	Lactobacillus_crispatus_SJ-3C-US	CBM
WP_005728199.1	CBM	CBM34	84.5	Lactobacillus_crispatus_SJ-3C-US	CBM
WP_005718438.1	CBM	CBM37	16.2	Lactobacillus_crispatus_SJ-3C-US	CBM
WP_005724991.1	CBM	CBM41	44.0	Lactobacillus_crispatus_SJ-3C-US	CBM
WP_005724991.1	CBM	CBM48	43.2	Lactobacillus_crispatus_SJ-3C-US	CBM
WP_005725874.1	CBM	CBM50	43.8	Lactobacillus_crispatus_SJ-3C-US	CBM
WP_005721518.1	CBM	CBM5	12.7	Lactobacillus_crispatus_SJ-3C-US	CBM
WP_005728417.1	CBM	CBM65	13.8	Lactobacillus_crispatus_SJ-3C-US	CBM
WP_005720567.1	CBM	CBM66	36.2	Lactobacillus_crispatus_SJ-3C-US	CBM
WP_068813721.1	CBM	CBM11	15.4	Lactobacillus_crispatus_UMB0040	CBM
WP_013085889.1	CBM	CBM12	12.9	Lactobacillus_crispatus_UMB0040	CBM
WP_068813412.1	CBM	CBM32	24.2	Lactobacillus_crispatus_UMB0040	CBM
WP_068813604.1	CBM	CBM34	83.2	Lactobacillus_crispatus_UMB0040	CBM
WP_101891133.1	CBM	CBM37	16.2	Lactobacillus_crispatus_UMB0040	CBM
WP_101891276.1	CBM	CBM41	44.0	Lactobacillus_crispatus_UMB0040	CBM
WP_101891276.1	CBM	CBM48	44.3	Lactobacillus_crispatus_UMB0040	CBM
WP_005725874.1	CBM	CBM50	43.8	Lactobacillus_crispatus_UMB0040	CBM
WP_005719393.1	CBM	CBM51	15.2	Lactobacillus_crispatus_UMB0040	CBM
WP_005721518.1	CBM	CBM5	12.7	Lactobacillus_crispatus_UMB0040	CBM
WP_068813676.1	CBM	CBM6	14.1	Lactobacillus_crispatus_UMB0040	CBM
WP_005728417.1	CBM	CBM65	13.8	Lactobacillus_crispatus_UMB0040	CBM
WP_005720567.1	CBM	CBM66	36.2	Lactobacillus_crispatus_UMB0040	CBM
WP_013086596.1	CBM	CBM67	15.3	Lactobacillus_crispatus_UMB0040	CBM
WP_101891266.1	CBM	CBM70	13.5	Lactobacillus_crispatus_UMB0040	CBM
WP_005727913.1	CBM	CBM12	15.3	Lactobacillus_crispatus_UMB0044	CBM
WP_005718343.1	CBM	CBM32	21.9	Lactobacillus_crispatus_UMB0044	CBM
WP_005720049.1	CBM	CBM34	84.5	Lactobacillus_crispatus_UMB0044	CBM
WP_005718438.1	CBM	CBM37	16.2	Lactobacillus_crispatus_UMB0044	CBM
WP_005719486.1	CBM	CBM41	44.0	Lactobacillus_crispatus_UMB0044	CBM
WP_005718977.1	CBM	CBM46	15.8	Lactobacillus_crispatus_UMB0044	CBM
WP_005719486.1	CBM	CBM48	44.2	Lactobacillus_crispatus_UMB0044	CBM
WP_005725874.1	CBM	CBM50	43.8	Lactobacillus_crispatus_UMB0044	CBM
WP_005725091.1	CBM	CBM50	36.1	Lactobacillus_crispatus_UMB0044	CBM
WP_005719041.1	CBM	CBM50	29.4	Lactobacillus_crispatus_UMB0044	CBM
WP_005719014.1	CBM	CBM50	22.1	Lactobacillus_crispatus_UMB0044	CBM
WP_005728417.1	CBM	CBM65	13.8	Lactobacillus_crispatus_UMB0044	CBM
WP_005720567.1	CBM	CBM66	36.2	Lactobacillus_crispatus_UMB0044	CBM
WP_005719548.1	CBM	CBM67	15.7	Lactobacillus_crispatus_UMB0044	CBM
WP_005724247.1	CBM	CBM12	13.1	Lactobacillus_crispatus_UMB0054	CBM
WP_101891528.1	CBM	CBM12	12.6	Lactobacillus_crispatus_UMB0054	CBM
WP_101891596.1	CBM	CBM32	23.2	Lactobacillus_crispatus_UMB0054	CBM
WP_005720049.1	CBM	CBM34	84.5	Lactobacillus_crispatus_UMB0054	CBM
WP_101891133.1	CBM	CBM37	16.2	Lactobacillus_crispatus_UMB0054	CBM

WP_005724991.1	CBM	CBM41	44.0	Lactobacillus_crispatus_UMB0054	CBM
WP_005724991.1	CBM	CBM48	43.2	Lactobacillus_crispatus_UMB0054	CBM
WP_005725874.1	CBM	CBM50	43.8	Lactobacillus_crispatus_UMB0054	CBM
WP_005719041.1	CBM	CBM50	29.4	Lactobacillus_crispatus_UMB0054	CBM
WP_005719014.1	CBM	CBM50	22.1	Lactobacillus_crispatus_UMB0054	CBM
WP_005719393.1	CBM	CBM51	15.2	Lactobacillus_crispatus_UMB0054	CBM
WP_005722705.1	CBM	CBM5	12.7	Lactobacillus_crispatus_UMB0054	CBM
WP_005720567.1	CBM	CBM66	36.2	Lactobacillus_crispatus_UMB0054	CBM
WP_068813721.1	CBM	CBM11	15.4	Lactobacillus_crispatus_UMB0085	CBM
WP_013085889.1	CBM	CBM12	12.9	Lactobacillus_crispatus_UMB0085	CBM
WP_068813884.1	CBM	CBM17	12.8	Lactobacillus_crispatus_UMB0085	CBM
WP_068813412.1	CBM	CBM32	24.2	Lactobacillus_crispatus_UMB0085	CBM
WP_068813604.1	CBM	CBM34	83.2	Lactobacillus_crispatus_UMB0085	CBM
WP_005724064.1	CBM	CBM37	16.2	Lactobacillus_crispatus_UMB0085	CBM
WP_005719486.1	CBM	CBM41	44.0	Lactobacillus_crispatus_UMB0085	CBM
WP_005719486.1	CBM	CBM48	44.2	Lactobacillus_crispatus_UMB0085	CBM
WP_005725874.1	CBM	CBM50	43.8	Lactobacillus_crispatus_UMB0085	CBM
WP_068812980.1	CBM	CBM50	40.0	Lactobacillus_crispatus_UMB0085	CBM
WP_101883647.1	CBM	CBM50	22.8	Lactobacillus_crispatus_UMB0085	CBM
WP_068813882.1	CBM	CBM51	15.4	Lactobacillus_crispatus_UMB0085	CBM
WP_005721518.1	CBM	CBM5	12.7	Lactobacillus_crispatus_UMB0085	CBM
WP_068813676.1	CBM	CBM6	14.1	Lactobacillus_crispatus_UMB0085	CBM
WP_005728417.1	CBM	CBM65	13.8	Lactobacillus_crispatus_UMB0085	CBM
WP_005720567.1	CBM	CBM66	36.2	Lactobacillus_crispatus_UMB0085	CBM
WP_013086596.1	CBM	CBM67	15.3	Lactobacillus_crispatus_UMB0085	CBM
WP_005727913.1	CBM	CBM12	15.3	Lactobacillus_crispatus_UMB0803	CBM
WP_005718343.1	CBM	CBM32	21.9	Lactobacillus_crispatus_UMB0803	CBM
WP_005720049.1	CBM	CBM34	84.5	Lactobacillus_crispatus_UMB0803	CBM
WP_005718438.1	CBM	CBM37	16.2	Lactobacillus_crispatus_UMB0803	CBM
WP_005725874.1	CBM	CBM50	43.8	Lactobacillus_crispatus_UMB0803	CBM
WP_005725113.1	CBM	CBM50	38.3	Lactobacillus_crispatus_UMB0803	CBM
WP_005725091.1	CBM	CBM50	36.1	Lactobacillus_crispatus_UMB0803	CBM
WP_005721518.1	CBM	CBM5	12.7	Lactobacillus_crispatus_UMB0803	CBM
WP_005728417.1	CBM	CBM65	13.8	Lactobacillus_crispatus_UMB0803	CBM
WP_005720567.1	CBM	CBM66	36.2	Lactobacillus_crispatus_UMB0803	CBM
WP_065989990.1	CBM	CBM12	13.1	Lactobacillus_crispatus_UMB0824	CBM
WP_101886974.1	CBM	CBM32	24.8	Lactobacillus_crispatus_UMB0824	CBM
WP_101887340.1	CBM	CBM34	84.0	Lactobacillus_crispatus_UMB0824	CBM
WP_101887002.1	CBM	CBM41	50.8	Lactobacillus_crispatus_UMB0824	CBM
WP_101887002.1	CBM	CBM48	42.6	Lactobacillus_crispatus_UMB0824	CBM
WP_005725874.1	CBM	CBM50	43.8	Lactobacillus_crispatus_UMB0824	CBM
WP_005721518.1	CBM	CBM5	12.7	Lactobacillus_crispatus_UMB0824	CBM
WP_089145832.1	CBM	CBM6	14.2	Lactobacillus_crispatus_UMB0824	CBM
WP_101887508.1	CBM	CBM66	36.0	Lactobacillus_crispatus_UMB0824	CBM
WP_101887370.1	CBM	CBM67	15.3	Lactobacillus_crispatus_UMB0824	CBM
WP_005720501.1	CBM	CBM12	13.5	Lactobacillus_crispatus_UMB1398	CBM
WP_101885609.1	CBM	CBM32	23.2	Lactobacillus_crispatus_UMB1398	CBM
WP_101885584.1	CBM	CBM34	84.5	Lactobacillus_crispatus_UMB1398	CBM
WP_005718438.1	CBM	CBM37	16.2	Lactobacillus_crispatus_UMB1398	CBM
WP_101885639.1	CBM	CBM41	44.0	Lactobacillus_crispatus_UMB1398	CBM
WP_005718977.1	CBM	CBM46	15.8	Lactobacillus_crispatus_UMB1398	CBM
WP_101885639.1	CBM	CBM48	43.2	Lactobacillus_crispatus_UMB1398	CBM
WP_020993138.1	CBM	CBM50	43.8	Lactobacillus_crispatus_UMB1398	CBM
WP_005719041.1	CBM	CBM50	29.4	Lactobacillus_crispatus_UMB1398	CBM
WP_101885513.1	CBM	CBM50	22.8	Lactobacillus_crispatus_UMB1398	CBM
WP_005719393.1	CBM	CBM51	15.2	Lactobacillus_crispatus_UMB1398	CBM
WP_005720567.1	CBM	CBM66	36.2	Lactobacillus_crispatus_UMB1398	CBM
WP_005719548.1	CBM	CBM67	15.7	Lactobacillus_crispatus_UMB1398	CBM
WP_060462441.1	CBM	CBM11	16.2	Lactobacillus_crispatus_VMC3	CBM
WP_060462070.1	CBM	CBM12	13.1	Lactobacillus_crispatus_VMC3	CBM
WP_060462429.1	CBM	CBM32	23.3	Lactobacillus_crispatus_VMC3	CBM
WP_060461794.1	CBM	CBM34	84.5	Lactobacillus_crispatus_VMC3	CBM
WP_057726229.1	CBM	CBM37	16.2	Lactobacillus_crispatus_VMC3	CBM
WP_005725874.1	CBM	CBM50	43.8	Lactobacillus_crispatus_VMC3	CBM
WP_005721518.1	CBM	CBM5	12.7	Lactobacillus_crispatus_VMC3	CBM
WP_060462616.1	CBM	CBM9	14.8	Lactobacillus_crispatus_VMC3	CBM
WP_060461674.1	CBM	CBM66	234.6	Lactobacillus_crispatus_VMC3	CBM
WP_060462088.1	CBM	CBM66	36.2	Lactobacillus_crispatus_VMC3	CBM
WP_060462408.1	CBM	CBM67	101.2	Lactobacillus_crispatus_VMC3	CBM
WP_060462003.1	CBM	CBM67	15.7	Lactobacillus_crispatus_VMC3	CBM
WP_005720501.1	CBM	CBM12	13.5	Lactobacillus_crispatus_VMC4	CBM
WP_005718343.1	CBM	CBM32	21.9	Lactobacillus_crispatus_VMC4	CBM
WP_005720049.1	CBM	CBM34	84.5	Lactobacillus_crispatus_VMC4	CBM
WP_005718438.1	CBM	CBM37	16.2	Lactobacillus_crispatus_VMC4	CBM
WP_081079761.1	CBM	CBM41	44.0	Lactobacillus_crispatus_VMC4	CBM
WP_081079761.1	CBM	CBM48	44.2	Lactobacillus_crispatus_VMC4	CBM
WP_020993138.1	CBM	CBM50	43.8	Lactobacillus_crispatus_VMC4	CBM
WP_060463456.1	CBM	CBM50	40.0	Lactobacillus_crispatus_VMC4	CBM
WP_060463493.1	CBM	CBM50	36.2	Lactobacillus_crispatus_VMC4	CBM
WP_060463506.1	CBM	CBM50	32.3	Lactobacillus_crispatus_VMC4	CBM
WP_133473092.1	CBM	CBM50	23.0	Lactobacillus_crispatus_VMC4	CBM
WP_005719393.1	CBM	CBM51	15.2	Lactobacillus_crispatus_VMC4	CBM
WP_005720567.1	CBM	CBM66	36.2	Lactobacillus_crispatus_VMC4	CBM
WP_005719548.1	CBM	CBM67	15.7	Lactobacillus_crispatus_VMC4	CBM
WP_005727913.1	CBM	CBM12	15.3	Lactobacillus_crispatus_VMC5	CBM
WP_060464023.1	CBM	CBM32	27.6	Lactobacillus_crispatus_VMC5	CBM
WP_005728199.1	CBM	CBM34	84.5	Lactobacillus_crispatus_VMC5	CBM

WP_005718438.1	CBM	CBM37	16.2	Lactobacillus_crispatus_VMC5	CBM
WP_005724991.1	CBM	CBM41	44.0	Lactobacillus_crispatus_VMC5	CBM
WP_005724991.1	CBM	CBM48	43.2	Lactobacillus_crispatus_VMC5	CBM
WP_005725874.1	CBM	CBM50	43.8	Lactobacillus_crispatus_VMC5	CBM
WP_005728964.1	CBM	CBM50	40.0	Lactobacillus_crispatus_VMC5	CBM
WP_005725113.1	CBM	CBM50	38.3	Lactobacillus_crispatus_VMC5	CBM
WP_005725091.1	CBM	CBM50	36.1	Lactobacillus_crispatus_VMC5	CBM
WP_005724903.1	CBM	CBM50	22.1	Lactobacillus_crispatus_VMC5	CBM
WP_005728417.1	CBM	CBM65	13.8	Lactobacillus_crispatus_VMC5	CBM
WP_005720567.1	CBM	CBM66	36.2	Lactobacillus_crispatus_VMC5	CBM
WP_060464344.1	CBM	CBM12	13.1	Lactobacillus_crispatus_VMC7	CBM
WP_060464341.1	CBM	CBM32	21.5	Lactobacillus_crispatus_VMC7	CBM
WP_005720049.1	CBM	CBM34	84.5	Lactobacillus_crispatus_VMC7	CBM
WP_005718438.1	CBM	CBM37	16.2	Lactobacillus_crispatus_VMC7	CBM
WP_005719486.1	CBM	CBM41	44.0	Lactobacillus_crispatus_VMC7	CBM
WP_005719486.1	CBM	CBM48	44.2	Lactobacillus_crispatus_VMC7	CBM
WP_005725874.1	CBM	CBM50	43.8	Lactobacillus_crispatus_VMC7	CBM
WP_005722705.1	CBM	CBM5	12.7	Lactobacillus_crispatus_VMC7	CBM
WP_005720567.1	CBM	CBM66	36.2	Lactobacillus_crispatus_VMC7	CBM
WP_060464192.1	CBM	CBM67	15.7	Lactobacillus_crispatus_VMC7	CBM
WP_005727913.1	CBM	CBM12	15.3	Lactobacillus_crispatus_VMC8	CBM
WP_060462936.1	CBM	CBM32	27.8	Lactobacillus_crispatus_VMC8	CBM
WP_005728199.1	CBM	CBM34	84.5	Lactobacillus_crispatus_VMC8	CBM
WP_005718438.1	CBM	CBM37	16.2	Lactobacillus_crispatus_VMC8	CBM
WP_005724991.1	CBM	CBM41	44.0	Lactobacillus_crispatus_VMC8	CBM
WP_005724991.1	CBM	CBM48	43.2	Lactobacillus_crispatus_VMC8	CBM
WP_060462836.1	CBM	CBM4	18.6	Lactobacillus_crispatus_VMC8	CBM
WP_060462857.1	CBM	CBM50	56.1	Lactobacillus_crispatus_VMC8	CBM
WP_060462944.1	CBM	CBM50	43.9	Lactobacillus_crispatus_VMC8	CBM
WP_005728964.1	CBM	CBM50	40.0	Lactobacillus_crispatus_VMC8	CBM
WP_005725113.1	CBM	CBM50	38.3	Lactobacillus_crispatus_VMC8	CBM
WP_005725091.1	CBM	CBM50	36.1	Lactobacillus_crispatus_VMC8	CBM
WP_060462882.1	CBM	CBM50	24.9	Lactobacillus_crispatus_VMC8	CBM
WP_005724903.1	CBM	CBM50	22.1	Lactobacillus_crispatus_VMC8	CBM
WP_060462836.1	CBM	CBM61	18.0	Lactobacillus_crispatus_VMC8	CBM
WP_005728417.1	CBM	CBM65	13.8	Lactobacillus_crispatus_VMC8	CBM
WP_005720567.1	CBM	CBM66	36.2	Lactobacillus_crispatus_VMC8	CBM
WP_013439368.1	CBM	CBM11	15.8	Lactobacillus_delbrueckii_UMB0003	CBM
WP_013438990.1	CBM	CBM32	45.2	Lactobacillus_delbrueckii_UMB0003	CBM
WP_013439633.1	CBM	CBM32	16.9	Lactobacillus_delbrueckii_UMB0003	CBM
WP_013438985.1	CBM	CBM34	89.7	Lactobacillus_delbrueckii_UMB0003	CBM
WP_013438985.1	CBM	CBM48	13.6	Lactobacillus_delbrueckii_UMB0003	CBM
WP_013438958.1	CBM	CBM50	105.9	Lactobacillus_delbrueckii_UMB0003	CBM
WP_013439398.1	CBM	CBM50	48.1	Lactobacillus_delbrueckii_UMB0003	CBM
WP_013439722.1	CBM	CBM50	39.8	Lactobacillus_delbrueckii_UMB0003	CBM
WP_013440066.1	CBM	CBM66	14.0	Lactobacillus_delbrueckii_UMB0003	CBM
WP_041811628.1	CBM	CBM67	14.0	Lactobacillus_delbrueckii_UMB0003	CBM
WP_101889163.1	CBM	CBM49	13.3	Lactobacillus_fermentum_UMB0187	CBM
WP_101889000.1	CBM	CBM50	229.4	Lactobacillus_fermentum_UMB0187	CBM
WP_101888759.1	CBM	CBM50	134.6	Lactobacillus_fermentum_UMB0187	CBM
WP_100184171.1	CBM	CBM50	44.2	Lactobacillus_fermentum_UMB0187	CBM
WP_101889135.1	CBM	CBM50	41.7	Lactobacillus_fermentum_UMB0187	CBM
WP_014562247.1	CBM	CBM50	36.3	Lactobacillus_fermentum_UMB0187	CBM
WP_101888823.1	CBM	CBM50	29.5	Lactobacillus_fermentum_UMB0187	CBM
WP_101888803.1	CBM	CBM50	25.7	Lactobacillus_fermentum_UMB0187	CBM
WP_012391639.1	CBM	CBM66	16.0	Lactobacillus_fermentum_UMB0187	CBM
WP_014562148.1	CBM	CBM67	20.6	Lactobacillus_fermentum_UMB0187	CBM
WP_003672153.1	CBM	CBM67	13.9	Lactobacillus_fermentum_UMB0187	CBM
WP_003650392.1	CBM	CBM11	15.1	Lactobacillus_gasseri_202-4	CBM
WP_003651966.1	CBM	CBM15	12.7	Lactobacillus_gasseri_202-4	CBM
WP_003651391.1	CBM	CBM32	38.4	Lactobacillus_gasseri_202-4	CBM
WP_003647893.1	CBM	CBM34	99.6	Lactobacillus_gasseri_202-4	CBM
WP_003647408.1	CBM	CBM50	37.9	Lactobacillus_gasseri_202-4	CBM
WP_003651799.1	CBM	CBM66	20.1	Lactobacillus_gasseri_202-4	CBM
WP_003651391.1	CBM	CBM32	38.4	Lactobacillus_gasseri_SJ-9E-US	CBM
WP_003647893.1	CBM	CBM34	99.6	Lactobacillus_gasseri_SJ-9E-US	CBM
WP_003647408.1	CBM	CBM50	37.9	Lactobacillus_gasseri_SJ-9E-US	CBM
WP_138272386.1	CBM	CBM50	22.9	Lactobacillus_gasseri_SJ-9E-US	CBM
WP_003655369.1	CBM	CBM66	15.0	Lactobacillus_gasseri_SJ-9E-US	CBM
WP_003651391.1	CBM	CBM32	38.4	Lactobacillus_gasseri_SV-16A-US	CBM
WP_035425071.1	CBM	CBM34	99.6	Lactobacillus_gasseri_SV-16A-US	CBM
WP_003647408.1	CBM	CBM50	37.9	Lactobacillus_gasseri_SV-16A-US	CBM
WP_003654218.1	CBM	CBM50	13.1	Lactobacillus_gasseri_SV-16A-US	CBM
WP_003655369.1	CBM	CBM66	15.0	Lactobacillus_gasseri_SV-16A-US	CBM
WP_003651966.1	CBM	CBM15	12.7	Lactobacillus_gasseri_UMB0045b	CBM
WP_003651391.1	CBM	CBM32	38.4	Lactobacillus_gasseri_UMB0045b	CBM
WP_003647893.1	CBM	CBM34	99.6	Lactobacillus_gasseri_UMB0045b	CBM
WP_003647408.1	CBM	CBM50	37.9	Lactobacillus_gasseri_UMB0045b	CBM
WP_003651799.1	CBM	CBM66	20.1	Lactobacillus_gasseri_UMB0045b	CBM
WP_003651966.1	CBM	CBM15	12.7	Lactobacillus_gasseri_UMB0045	CBM
WP_003651391.1	CBM	CBM32	38.4	Lactobacillus_gasseri_UMB0045	CBM
WP_003647893.1	CBM	CBM34	99.6	Lactobacillus_gasseri_UMB0045	CBM
WP_003647408.1	CBM	CBM50	37.9	Lactobacillus_gasseri_UMB0045	CBM
WP_003651799.1	CBM	CBM66	20.1	Lactobacillus_gasseri_UMB0045	CBM
WP_039156886.1	CBM	CBM11	15.3	Lactobacillus_gasseri_UMB0099	CBM
WP_003649215.1	CBM	CBM12	13.4	Lactobacillus_gasseri_UMB0099	CBM
WP_101890904.1	CBM	CBM32	38.6	Lactobacillus_gasseri_UMB0099	CBM

WP_003649526.1	CBM	CBM34	99.8	Lactobacillus_gasseri_UMB0099	CBM
WP_101890772.1	CBM	CBM50	36.4	Lactobacillus_gasseri_UMB0099	CBM
WP_077958771.1	CBM	CBM54	13.3	Lactobacillus_gasseri_UMB0099	CBM
WP_101891075.1	CBM	CBM66	19.2	Lactobacillus_gasseri_UMB0099	CBM
TVV24203.1	CBM	CBM34	99.6	Lactobacillus_gasseri_UMB1399	CBM
TVV20913.1	CBM	CBM50	37.9	Lactobacillus_gasseri_UMB1399	CBM
TVV23980.1	CBM	CBM66	20.1	Lactobacillus_gasseri_UMB1399	CBM
WP_035511957.1	CBM	CBM32	57.9	Lactobacillus_iners_ATCC55195	CBM
WP_006737124.1	CBM	CBM32	26.9	Lactobacillus_iners_ATCC55195	CBM
WP_006730276.1	CBM	CBM34	96.0	Lactobacillus_iners_ATCC55195	CBM
WP_006735116.1	CBM	CBM38	14.6	Lactobacillus_iners_ATCC55195	CBM
WP_006736746.1	CBM	CBM41	87.6	Lactobacillus_iners_ATCC55195	CBM
WP_035511957.1	CBM	CBM47	28.6	Lactobacillus_iners_ATCC55195	CBM
WP_006736746.1	CBM	CBM48	41.3	Lactobacillus_iners_ATCC55195	CBM
WP_006736930.1	CBM	CBM48	35.4	Lactobacillus_iners_ATCC55195	CBM
WP_006736235.1	CBM	CBM48	35.4	Lactobacillus_iners_ATCC55195	CBM
WP_035511957.1	CBM	CBM51	91.3	Lactobacillus_iners_ATCC55195	CBM
WP_006731315.1	CBM	CBM56	15.5	Lactobacillus_iners_ATCC55195	CBM
WP_006736746.1	CBM	CBM60	15.9	Lactobacillus_iners_ATCC55195	CBM
WP_006736783.1	CBM	CBM64	13.4	Lactobacillus_iners_ATCC55195	CBM
WP_010221941.1	CBM	CBM32	57.8	Lactobacillus_iners_DSM13335	CBM
WP_006729357.1	CBM	CBM32	24.8	Lactobacillus_iners_DSM13335	CBM
WP_006729724.1	CBM	CBM34	96.0	Lactobacillus_iners_DSM13335	CBM
WP_006729959.1	CBM	CBM38	14.6	Lactobacillus_iners_DSM13335	CBM
WP_006729871.1	CBM	CBM40	12.9	Lactobacillus_iners_DSM13335	CBM
WP_006728964.1	CBM	CBM41	87.1	Lactobacillus_iners_DSM13335	CBM
WP_010221941.1	CBM	CBM47	28.4	Lactobacillus_iners_DSM13335	CBM
WP_006728964.1	CBM	CBM48	41.0	Lactobacillus_iners_DSM13335	CBM
WP_006729893.1	CBM	CBM48	35.4	Lactobacillus_iners_DSM13335	CBM
WP_006729889.1	CBM	CBM48	35.3	Lactobacillus_iners_DSM13335	CBM
WP_010221941.1	CBM	CBM51	91.1	Lactobacillus_iners_DSM13335	CBM
WP_006728964.1	CBM	CBM60	13.9	Lactobacillus_iners_DSM13335	CBM
WP_006729768.1	CBM	CBM64	13.3	Lactobacillus_iners_DSM13335	CBM
WP_006735769.1	CBM	CBM11	13.0	Lactobacillus_iners_LEAF2052A-d	CBM
WP_006735654.1	CBM	CBM32	57.9	Lactobacillus_iners_LEAF2052A-d	CBM
WP_006735652.1	CBM	CBM32	26.6	Lactobacillus_iners_LEAF2052A-d	CBM
WP_006735377.1	CBM	CBM34	96.0	Lactobacillus_iners_LEAF2052A-d	CBM
WP_006731889.1	CBM	CBM38	14.6	Lactobacillus_iners_LEAF2052A-d	CBM
WP_006735465.1	CBM	CBM41	85.7	Lactobacillus_iners_LEAF2052A-d	CBM
WP_006735654.1	CBM	CBM47	28.4	Lactobacillus_iners_LEAF2052A-d	CBM
WP_006735465.1	CBM	CBM48	41.0	Lactobacillus_iners_LEAF2052A-d	CBM
WP_050774287.1	CBM	CBM48	36.0	Lactobacillus_iners_LEAF2052A-d	CBM
WP_006730145.1	CBM	CBM48	35.4	Lactobacillus_iners_LEAF2052A-d	CBM
WP_006735654.1	CBM	CBM51	91.3	Lactobacillus_iners_LEAF2052A-d	CBM
WP_006735465.1	CBM	CBM60	14.7	Lactobacillus_iners_LEAF2052A-d	CBM
WP_006735409.1	CBM	CBM64	13.3	Lactobacillus_iners_LEAF2052A-d	CBM
WP_006734139.1	CBM	CBM11	13.2	Lactobacillus_iners_SPIN2503V10-d	CBM
WP_006734027.1	CBM	CBM32	58.0	Lactobacillus_iners_SPIN2503V10-d	CBM
WP_006734037.1	CBM	CBM32	25.3	Lactobacillus_iners_SPIN2503V10-d	CBM
WP_006734339.1	CBM	CBM38	14.6	Lactobacillus_iners_SPIN2503V10-d	CBM
WP_006734523.1	CBM	CBM40	11.8	Lactobacillus_iners_SPIN2503V10-d	CBM
WP_006734460.1	CBM	CBM41	90.2	Lactobacillus_iners_SPIN2503V10-d	CBM
WP_006734027.1	CBM	CBM47	28.7	Lactobacillus_iners_SPIN2503V10-d	CBM
WP_006734460.1	CBM	CBM48	41.3	Lactobacillus_iners_SPIN2503V10-d	CBM
WP_050777054.1	CBM	CBM48	35.9	Lactobacillus_iners_SPIN2503V10-d	CBM
WP_006734027.1	CBM	CBM51	91.4	Lactobacillus_iners_SPIN2503V10-d	CBM
WP_006734460.1	CBM	CBM60	15.5	Lactobacillus_iners_SPIN2503V10-d	CBM
WP_102720283.1	CBM	CBM32	57.8	Lactobacillus_iners_UMB0030	CBM
WP_102720271.1	CBM	CBM32	25.3	Lactobacillus_iners_UMB0030	CBM
WP_006730276.1	CBM	CBM34	96.0	Lactobacillus_iners_UMB0030	CBM
WP_006734339.1	CBM	CBM38	14.6	Lactobacillus_iners_UMB0030	CBM
WP_006734523.1	CBM	CBM40	11.8	Lactobacillus_iners_UMB0030	CBM
WP_102720455.1	CBM	CBM41	88.3	Lactobacillus_iners_UMB0030	CBM
WP_102720283.1	CBM	CBM47	28.2	Lactobacillus_iners_UMB0030	CBM
WP_102720455.1	CBM	CBM48	41.3	Lactobacillus_iners_UMB0030	CBM
WP_006736235.1	CBM	CBM48	35.4	Lactobacillus_iners_UMB0030	CBM
WP_102720398.1	CBM	CBM48	35.2	Lactobacillus_iners_UMB0030	CBM
WP_102720283.1	CBM	CBM51	91.1	Lactobacillus_iners_UMB0030	CBM
WP_102720455.1	CBM	CBM60	16.1	Lactobacillus_iners_UMB0030	CBM
WP_006729768.1	CBM	CBM64	13.3	Lactobacillus_iners_UMB0030	CBM
WP_102215359.1	CBM	CBM32	55.3	Lactobacillus_iners_UMB0033	CBM
WP_102215376.1	CBM	CBM32	24.9	Lactobacillus_iners_UMB0033	CBM
WP_102215514.1	CBM	CBM34	97.2	Lactobacillus_iners_UMB0033	CBM
WP_006734339.1	CBM	CBM38	14.6	Lactobacillus_iners_UMB0033	CBM
WP_102215423.1	CBM	CBM41	87.2	Lactobacillus_iners_UMB0033	CBM
WP_102215359.1	CBM	CBM47	25.7	Lactobacillus_iners_UMB0033	CBM
WP_102215423.1	CBM	CBM48	41.3	Lactobacillus_iners_UMB0033	CBM
WP_006730145.1	CBM	CBM48	35.4	Lactobacillus_iners_UMB0033	CBM
WP_102215235.1	CBM	CBM48	35.0	Lactobacillus_iners_UMB0033	CBM
WP_102215567.1	CBM	CBM50	52.6	Lactobacillus_iners_UMB0033	CBM
WP_102215551.1	CBM	CBM50	37.9	Lactobacillus_iners_UMB0033	CBM
WP_102215359.1	CBM	CBM51	90.9	Lactobacillus_iners_UMB0033	CBM
WP_006731315.1	CBM	CBM56	15.5	Lactobacillus_iners_UMB0033	CBM
WP_102215423.1	CBM	CBM60	14.5	Lactobacillus_iners_UMB0033	CBM
WP_102215506.1	CBM	CBM64	13.3	Lactobacillus_iners_UMB0033	CBM
WP_102695591.1	CBM	CBM32	25.3	Lactobacillus_iners_UMB1051	CBM
WP_006730276.1	CBM	CBM34	96.0	Lactobacillus_iners_UMB1051	CBM

WP_006734339.1	CBM	CBM38	14.6	Lactobacillus_iners_UMB1051	CBM
WP_006734523.1	CBM	CBM40	11.8	Lactobacillus_iners_UMB1051	CBM
WP_006736668.1	CBM	CBM48	35.4	Lactobacillus_iners_UMB1051	CBM
WP_102695329.1	CBM	CBM48	35.2	Lactobacillus_iners_UMB1051	CBM
WP_006736091.1	CBM	CBM56	15.5	Lactobacillus_iners_UMB1051	CBM
WP_006734944.1	CBM	CBM64	13.4	Lactobacillus_iners_UMB1051	CBM
WP_006586831.1	CBM	CBM11	16.7	Lactobacillus_jensenii_115-3-CHN	CBM
WP_022091380.1	CBM	CBM32	29.9	Lactobacillus_jensenii_115-3-CHN	CBM
WP_006585985.1	CBM	CBM34	98.1	Lactobacillus_jensenii_115-3-CHN	CBM
WP_006588937.1	CBM	CBM37	13.7	Lactobacillus_jensenii_115-3-CHN	CBM
WP_006587529.1	CBM	CBM4	14.0	Lactobacillus_jensenii_115-3-CHN	CBM
WP_006587239.1	CBM	CBM50	43.8	Lactobacillus_jensenii_115-3-CHN	CBM
WP_006586905.1	CBM	CBM61	14.3	Lactobacillus_jensenii_115-3-CHN	CBM
WP_006586883.1	CBM	CBM66	21.2	Lactobacillus_jensenii_115-3-CHN	CBM
WP_006587322.1	CBM	CBM67	14.8	Lactobacillus_jensenii_115-3-CHN	CBM
WP_006584838.1	CBM	CBM11	17.7	Lactobacillus_jensenii_269-3	CBM
WP_006587747.1	CBM	CBM32	28.2	Lactobacillus_jensenii_269-3	CBM
WP_006585326.1	CBM	CBM34	97.6	Lactobacillus_jensenii_269-3	CBM
WP_006584589.1	CBM	CBM50	42.3	Lactobacillus_jensenii_269-3	CBM
WP_006584900.1	CBM	CBM61	14.7	Lactobacillus_jensenii_269-3	CBM
WP_006584838.1	CBM	CBM11	17.7	Lactobacillus_jensenii_SJ-7A-US	CBM
WP_006587747.1	CBM	CBM32	28.2	Lactobacillus_jensenii_SJ-7A-US	CBM
WP_006585326.1	CBM	CBM34	97.6	Lactobacillus_jensenii_SJ-7A-US	CBM
WP_006584589.1	CBM	CBM50	42.3	Lactobacillus_jensenii_SJ-7A-US	CBM
WP_006584838.1	CBM	CBM11	17.7	Lactobacillus_jensenii_UMB0007	CBM
WP_006587747.1	CBM	CBM32	28.2	Lactobacillus_jensenii_UMB0007	CBM
WP_006585326.1	CBM	CBM34	97.6	Lactobacillus_jensenii_UMB0007	CBM
WP_006584589.1	CBM	CBM50	42.3	Lactobacillus_jensenii_UMB0007	CBM
WP_144850924.1	CBM	CBM11	17.2	Lactobacillus_jensenii_UMB0034	CBM
WP_006587747.1	CBM	CBM32	28.2	Lactobacillus_jensenii_UMB0034	CBM
WP_006585326.1	CBM	CBM34	97.6	Lactobacillus_jensenii_UMB0034	CBM
WP_006584589.1	CBM	CBM50	42.3	Lactobacillus_jensenii_UMB0034	CBM
WP_075362454.1	CBM	CBM61	14.5	Lactobacillus_jensenii_UMB0034	CBM
WP_006584838.1	CBM	CBM11	17.7	Lactobacillus_jensenii_UMB0037	CBM
WP_006587747.1	CBM	CBM32	28.2	Lactobacillus_jensenii_UMB0037	CBM
WP_144798779.1	CBM	CBM34	97.1	Lactobacillus_jensenii_UMB0037	CBM
WP_006584589.1	CBM	CBM50	42.3	Lactobacillus_jensenii_UMB0037	CBM
WP_144798644.1	CBM	CBM61	17.5	Lactobacillus_jensenii_UMB0037	CBM
WP_006584838.1	CBM	CBM11	17.7	Lactobacillus_jensenii_UMB0077	CBM
WP_006587747.1	CBM	CBM32	28.2	Lactobacillus_jensenii_UMB0077	CBM
WP_006585326.1	CBM	CBM34	97.6	Lactobacillus_jensenii_UMB0077	CBM
WP_006584589.1	CBM	CBM50	42.3	Lactobacillus_jensenii_UMB0077	CBM
WP_101850180.1	CBM	CBM53	15.0	Lactobacillus_jensenii_UMB0077	CBM
WP_075362454.1	CBM	CBM61	14.5	Lactobacillus_jensenii_UMB0077	CBM
WP_006584838.1	CBM	CBM11	17.7	Lactobacillus_jensenii_UMB1307	CBM
WP_006587747.1	CBM	CBM32	28.2	Lactobacillus_jensenii_UMB1307	CBM
WP_006585326.1	CBM	CBM34	97.6	Lactobacillus_jensenii_UMB1307	CBM
WP_006584589.1	CBM	CBM50	42.3	Lactobacillus_jensenii_UMB1307	CBM
WP_075362454.1	CBM	CBM61	14.5	Lactobacillus_jensenii_UMB1307	CBM
WP_006586831.1	CBM	CBM11	16.7	Lactobacillus_jensenii_UMB1355	CBM
WP_022091380.1	CBM	CBM32	29.9	Lactobacillus_jensenii_UMB1355	CBM
WP_144772296.1	CBM	CBM34	98.1	Lactobacillus_jensenii_UMB1355	CBM
WP_144772339.1	CBM	CBM37	13.9	Lactobacillus_jensenii_UMB1355	CBM
WP_006587529.1	CBM	CBM4	14.0	Lactobacillus_jensenii_UMB1355	CBM
WP_006586905.1	CBM	CBM61	14.3	Lactobacillus_jensenii_UMB1355	CBM
WP_006586883.1	CBM	CBM66	21.2	Lactobacillus_jensenii_UMB1355	CBM
WP_144772236.1	CBM	CBM67	13.9	Lactobacillus_jensenii_UMB1355	CBM
WP_003567052.1	CBM	CBM12	13.5	Lactobacillus_rhamnosus_51B	CBM
WP_005690458.1	CBM	CBM30	14.0	Lactobacillus_rhamnosus_51B	CBM
WP_005690728.1	CBM	CBM32	54.8	Lactobacillus_rhamnosus_51B	CBM
WP_014571231.1	CBM	CBM34	101.4	Lactobacillus_rhamnosus_51B	CBM
WP_005692830.1	CBM	CBM34	25.1	Lactobacillus_rhamnosus_51B	CBM
WP_005693009.1	CBM	CBM35	14.6	Lactobacillus_rhamnosus_51B	CBM
WP_005691421.1	CBM	CBM36	15.3	Lactobacillus_rhamnosus_51B	CBM
WP_005690728.1	CBM	CBM47	14.2	Lactobacillus_rhamnosus_51B	CBM
WP_014571465.1	CBM	CBM48	63.2	Lactobacillus_rhamnosus_51B	CBM
WP_032956665.1	CBM	CBM50	47.6	Lactobacillus_rhamnosus_51B	CBM
WP_032957311.1	CBM	CBM50	42.2	Lactobacillus_rhamnosus_51B	CBM
WP_005689277.1	CBM	CBM50	40.8	Lactobacillus_rhamnosus_51B	CBM
WP_005691582.1	CBM	CBM50	37.8	Lactobacillus_rhamnosus_51B	CBM
WP_014571440.1	CBM	CBM50	28.3	Lactobacillus_rhamnosus_51B	CBM
WP_014571436.1	CBM	CBM50	21.5	Lactobacillus_rhamnosus_51B	CBM
WP_005690316.1	CBM	CBM66	17.9	Lactobacillus_rhamnosus_51B	CBM
WP_032957200.1	CBM	CBM67	73.9	Lactobacillus_rhamnosus_51B	CBM
WP_014571015.1	CBM	CBM78	14.1	Lactobacillus_rhamnosus_51B	CBM
WP_003717418.1	CBM	CBM12	12.4	Lactobacillus_vaginalis_ATCC49540	CBM
WP_003717885.1	CBM	CBM37	15.8	Lactobacillus_vaginalis_ATCC49540	CBM
WP_003716398.1	CBM	CBM50	255.2	Lactobacillus_vaginalis_ATCC49540	CBM
WP_003717892.1	CBM	CBM50	145.2	Lactobacillus_vaginalis_ATCC49540	CBM
WP_003716856.1	CBM	CBM50	47.7	Lactobacillus_vaginalis_ATCC49540	CBM
WP_003717627.1	CBM	CBM50	44.1	Lactobacillus_vaginalis_ATCC49540	CBM
WP_003717211.1	CBM	CBM50	43.3	Lactobacillus_vaginalis_ATCC49540	CBM
WP_003717208.1	CBM	CBM50	43.2	Lactobacillus_vaginalis_ATCC49540	CBM
WP_003717015.1	CBM	CBM50	28.1	Lactobacillus_vaginalis_ATCC49540	CBM
WP_003717418.1	CBM	CBM5	15.8	Lactobacillus_vaginalis_ATCC49540	CBM
WP_040531453.1	CBM	CBM61	13.8	Lactobacillus_vaginalis_ATCC49540	CBM
WP_003717500.1	CBM	CBM67	18.2	Lactobacillus_vaginalis_ATCC49540	CBM

WP_036854677.1	CBM	CBM16	31.6	Prevotella_amnii_DNF00058	CBM
WP_036853695.1	CBM	CBM20	70.9	Prevotella_amnii_DNF00058	CBM
WP_024992730.1	CBM	CBM20	14.8	Prevotella_amnii_DNF00058	CBM
WP_036856540.1	CBM	CBM20	14.7	Prevotella_amnii_DNF00058	CBM
WP_036856035.1	CBM	CBM26	51.2	Prevotella_amnii_DNF00058	CBM
WP_036854073.1	CBM	CBM32	34.1	Prevotella_amnii_DNF00058	CBM
WP_036853945.1	CBM	CBM32	20.1	Prevotella_amnii_DNF00058	CBM
WP_036853787.1	CBM	CBM34	14.7	Prevotella_amnii_DNF00058	CBM
WP_036854677.1	CBM	CBM37	15.6	Prevotella_amnii_DNF00058	CBM
WP_036854279.1	CBM	CBM3	14.4	Prevotella_amnii_DNF00058	CBM
WP_036855277.1	CBM	CBM3	13.5	Prevotella_amnii_DNF00058	CBM
WP_036856540.1	CBM	CBM48	40.1	Prevotella_amnii_DNF00058	CBM
WP_036855513.1	CBM	CBM49	13.1	Prevotella_amnii_DNF00058	CBM
WP_036854677.1	CBM	CBM4	19.1	Prevotella_amnii_DNF00058	CBM
WP_036854181.1	CBM	CBM50	51.8	Prevotella_amnii_DNF00058	CBM
WP_036855085.1	CBM	CBM50	37.7	Prevotella_amnii_DNF00058	CBM
WP_008447567.1	CBM	CBM50	22.0	Prevotella_amnii_DNF00058	CBM
WP_008450143.1	CBM	CBM60	14.2	Prevotella_amnii_DNF00058	CBM
WP_036854677.1	CBM	CBM61	26.5	Prevotella_amnii_DNF00058	CBM
WP_036854677.1	CBM	CBM8	15.2	Prevotella_amnii_DNF00058	CBM
WP_036854083.1	CBM	CBM9	30.1	Prevotella_amnii_DNF00058	CBM
WP_036853948.1	CBM	CBM66	19.3	Prevotella_amnii_DNF00058	CBM
WP_036855622.1	CBM	CBM67	29.5	Prevotella_amnii_DNF00058	CBM
WP_036854472.1	CBM	CBM73	12.7	Prevotella_amnii_DNF00058	CBM
WP_060932750.1	CBM	CBM16	30.9	Prevotella_amnii_DNF00307	CBM
WP_036853695.1	CBM	CBM20	70.9	Prevotella_amnii_DNF00307	CBM
WP_060932747.1	CBM	CBM20	15.2	Prevotella_amnii_DNF00307	CBM
WP_024992730.1	CBM	CBM20	14.8	Prevotella_amnii_DNF00307	CBM
WP_060932772.1	CBM	CBM26	51.2	Prevotella_amnii_DNF00307	CBM
WP_060932664.1	CBM	CBM32	36.8	Prevotella_amnii_DNF00307	CBM
WP_060932855.1	CBM	CBM32	22.4	Prevotella_amnii_DNF00307	CBM
WP_060932750.1	CBM	CBM37	14.7	Prevotella_amnii_DNF00307	CBM
WP_060932728.1	CBM	CBM3	14.4	Prevotella_amnii_DNF00307	CBM
WP_060932747.1	CBM	CBM48	39.5	Prevotella_amnii_DNF00307	CBM
WP_060932681.1	CBM	CBM49	13.1	Prevotella_amnii_DNF00307	CBM
WP_060932750.1	CBM	CBM4	17.6	Prevotella_amnii_DNF00307	CBM
WP_060932952.1	CBM	CBM50	51.5	Prevotella_amnii_DNF00307	CBM
WP_036855085.1	CBM	CBM50	37.7	Prevotella_amnii_DNF00307	CBM
WP_060932876.1	CBM	CBM50	22.7	Prevotella_amnii_DNF00307	CBM
WP_019035691.1	CBM	CBM60	16.8	Prevotella_amnii_DNF00307	CBM
WP_060932750.1	CBM	CBM61	24.4	Prevotella_amnii_DNF00307	CBM
WP_060932750.1	CBM	CBM8	13.7	Prevotella_amnii_DNF00307	CBM
WP_060932661.1	CBM	CBM9	28.4	Prevotella_amnii_DNF00307	CBM
WP_060932913.1	CBM	CBM63	13.1	Prevotella_amnii_DNF00307	CBM
WP_060932857.1	CBM	CBM66	20.3	Prevotella_amnii_DNF00307	CBM
WP_060932813.1	CBM	CBM67	29.5	Prevotella_amnii_DNF00307	CBM
WP_008449381.1	CBM	CBM73	12.7	Prevotella_amnii_DNF00307	CBM
WP_036886371.1	CBM	CBM13	21.4	Prevotella_bivia_DNF00188	CBM
WP_036887723.1	CBM	CBM16	17.4	Prevotella_bivia_DNF00188	CBM
WP_036886124.1	CBM	CBM20	65.6	Prevotella_bivia_DNF00188	CBM
WP_036886071.1	CBM	CBM26	56.3	Prevotella_bivia_DNF00188	CBM
WP_036886551.1	CBM	CBM32	38.1	Prevotella_bivia_DNF00188	CBM
WP_036886193.1	CBM	CBM32	37.6	Prevotella_bivia_DNF00188	CBM
WP_036861930.1	CBM	CBM34	14.2	Prevotella_bivia_DNF00188	CBM
WP_080728706.1	CBM	CBM35	14.8	Prevotella_bivia_DNF00188	CBM
WP_036887723.1	CBM	CBM37	17.1	Prevotella_bivia_DNF00188	CBM
WP_036861824.1	CBM	CBM48	35.6	Prevotella_bivia_DNF00188	CBM
WP_036887723.1	CBM	CBM4	18.4	Prevotella_bivia_DNF00188	CBM
WP_036887374.1	CBM	CBM50	57.7	Prevotella_bivia_DNF00188	CBM
WP_036862700.1	CBM	CBM50	31.2	Prevotella_bivia_DNF00188	CBM
WP_036887627.1	CBM	CBM50	22.6	Prevotella_bivia_DNF00188	CBM
WP_036887723.1	CBM	CBM61	18.4	Prevotella_bivia_DNF00188	CBM
WP_036864555.1	CBM	CBM9	138.3	Prevotella_bivia_DNF00188	CBM
WP_036886567.1	CBM	CBM9	31.1	Prevotella_bivia_DNF00188	CBM
WP_036887325.1	CBM	CBM66	23.1	Prevotella_bivia_DNF00188	CBM
WP_036866282.1	CBM	CBM13	21.3	Prevotella_bivia_DNF00320	CBM
WP_036868425.1	CBM	CBM16	17.4	Prevotella_bivia_DNF00320	CBM
WP_036865639.1	CBM	CBM20	65.0	Prevotella_bivia_DNF00320	CBM
WP_036865774.1	CBM	CBM26	56.3	Prevotella_bivia_DNF00320	CBM
WP_036866570.1	CBM	CBM32	38.1	Prevotella_bivia_DNF00320	CBM
WP_036865822.1	CBM	CBM32	36.4	Prevotella_bivia_DNF00320	CBM
WP_036868309.1	CBM	CBM34	14.2	Prevotella_bivia_DNF00320	CBM
WP_080728626.1	CBM	CBM35	14.4	Prevotella_bivia_DNF00320	CBM
WP_036868425.1	CBM	CBM37	17.1	Prevotella_bivia_DNF00320	CBM
WP_036861824.1	CBM	CBM48	35.6	Prevotella_bivia_DNF00320	CBM
WP_036868425.1	CBM	CBM4	16.8	Prevotella_bivia_DNF00320	CBM
WP_036868688.1	CBM	CBM50	57.7	Prevotella_bivia_DNF00320	CBM
WP_036862700.1	CBM	CBM50	31.2	Prevotella_bivia_DNF00320	CBM
WP_036866519.1	CBM	CBM50	22.7	Prevotella_bivia_DNF00320	CBM
WP_036868425.1	CBM	CBM61	18.6	Prevotella_bivia_DNF00320	CBM
WP_036868734.1	CBM	CBM9	135.1	Prevotella_bivia_DNF00320	CBM
WP_036866562.1	CBM	CBM9	32.1	Prevotella_bivia_DNF00320	CBM
WP_036866007.1	CBM	CBM66	23.1	Prevotella_bivia_DNF00320	CBM
WP_036861685.1	CBM	CBM13	21.2	Prevotella_bivia_DNF00650	CBM
WP_036864304.1	CBM	CBM16	23.8	Prevotella_bivia_DNF00650	CBM
WP_036862732.1	CBM	CBM20	65.6	Prevotella_bivia_DNF00650	CBM
WP_036862213.1	CBM	CBM26	56.3	Prevotella_bivia_DNF00650	CBM

WP_036862008.1	CBM	CBM32	38.1	Prevotella_bivia_DNF00650	CBM
WP_036862875.1	CBM	CBM32	37.4	Prevotella_bivia_DNF00650	CBM
WP_036861930.1	CBM	CBM34	14.2	Prevotella_bivia_DNF00650	CBM
WP_080728540.1	CBM	CBM35	14.6	Prevotella_bivia_DNF00650	CBM
WP_036864304.1	CBM	CBM37	17.1	Prevotella_bivia_DNF00650	CBM
WP_036864659.1	CBM	CBM43	13.4	Prevotella_bivia_DNF00650	CBM
WP_036861824.1	CBM	CBM48	35.6	Prevotella_bivia_DNF00650	CBM
WP_036864304.1	CBM	CBM4	18.3	Prevotella_bivia_DNF00650	CBM
WP_036862066.1	CBM	CBM50	57.7	Prevotella_bivia_DNF00650	CBM
WP_036862700.1	CBM	CBM50	31.2	Prevotella_bivia_DNF00650	CBM
WP_036862383.1	CBM	CBM50	22.2	Prevotella_bivia_DNF00650	CBM
WP_036864304.1	CBM	CBM61	20.0	Prevotella_bivia_DNF00650	CBM
WP_036864555.1	CBM	CBM9	138.3	Prevotella_bivia_DNF00650	CBM
WP_036862018.1	CBM	CBM9	30.0	Prevotella_bivia_DNF00650	CBM
WP_036861114.1	CBM	CBM66	23.1	Prevotella_bivia_DNF00650	CBM
WP_061450536.1	CBM	CBM13	21.0	Prevotella_bivia_GED7760C	CBM
WP_061315200.1	CBM	CBM16	19.5	Prevotella_bivia_GED7760C	CBM
WP_061450341.1	CBM	CBM20	65.6	Prevotella_bivia_GED7760C	CBM
WP_061450434.1	CBM	CBM26	56.3	Prevotella_bivia_GED7760C	CBM
WP_061450120.1	CBM	CBM32	37.6	Prevotella_bivia_GED7760C	CBM
WP_061450059.1	CBM	CBM32	37.5	Prevotella_bivia_GED7760C	CBM
WP_080728706.1	CBM	CBM35	14.8	Prevotella_bivia_GED7760C	CBM
WP_061315200.1	CBM	CBM37	17.1	Prevotella_bivia_GED7760C	CBM
WP_061450193.1	CBM	CBM48	35.6	Prevotella_bivia_GED7760C	CBM
WP_061450351.1	CBM	CBM4	21.0	Prevotella_bivia_GED7760C	CBM
WP_061315200.1	CBM	CBM4	19.0	Prevotella_bivia_GED7760C	CBM
WP_061450040.1	CBM	CBM50	57.6	Prevotella_bivia_GED7760C	CBM
WP_061450339.1	CBM	CBM50	31.2	Prevotella_bivia_GED7760C	CBM
WP_061315376.1	CBM	CBM50	27.4	Prevotella_bivia_GED7760C	CBM
WP_061450336.1	CBM	CBM50	22.8	Prevotella_bivia_GED7760C	CBM
WP_061315200.1	CBM	CBM61	19.2	Prevotella_bivia_GED7760C	CBM
WP_061450623.1	CBM	CBM9	138.0	Prevotella_bivia_GED7760C	CBM
WP_036886567.1	CBM	CBM9	31.1	Prevotella_bivia_GED7760C	CBM
WP_061450225.1	CBM	CBM66	23.0	Prevotella_bivia_GED7760C	CBM
WP_061450352.1	CBM	CBM66	18.0	Prevotella_bivia_GED7760C	CBM
WP_060940599.1	CBM	CBM20	55.7	Prevotella_corporis_MJR7716	CBM
WP_060939937.1	CBM	CBM22	17.5	Prevotella_corporis_MJR7716	CBM
WP_060940140.1	CBM	CBM32	15.8	Prevotella_corporis_MJR7716	CBM
WP_082745758.1	CBM	CBM32	15.3	Prevotella_corporis_MJR7716	CBM
WP_156439272.1	CBM	CBM35	23.3	Prevotella_corporis_MJR7716	CBM
WP_060940318.1	CBM	CBM48	43.1	Prevotella_corporis_MJR7716	CBM
WP_060940127.1	CBM	CBM49	13.5	Prevotella_corporis_MJR7716	CBM
WP_060941039.1	CBM	CBM50	44.1	Prevotella_corporis_MJR7716	CBM
WP_025875133.1	CBM	CBM50	33.9	Prevotella_corporis_MJR7716	CBM
WP_060941011.1	CBM	CBM50	28.6	Prevotella_corporis_MJR7716	CBM
WP_082745880.1	CBM	CBM52	14.6	Prevotella_corporis_MJR7716	CBM
WP_156439272.1	CBM	CBM8	19.4	Prevotella_corporis_MJR7716	CBM
WP_060941472.1	CBM	CBM66	15.3	Prevotella_corporis_MJR7716	CBM
WP_028901488.1	CBM	CBM76	13.6	Prevotella_corporis_MJR7716	CBM
WP_036855259.1	CBM	CBM20	78.4	Prevotella_denticola_DNF00960	CBM
WP_036853772.1	CBM	CBM32	67.3	Prevotella_denticola_DNF00960	CBM
WP_025067264.1	CBM	CBM32	62.8	Prevotella_denticola_DNF00960	CBM
WP_036854991.1	CBM	CBM32	46.0	Prevotella_denticola_DNF00960	CBM
WP_036853405.1	CBM	CBM32	37.4	Prevotella_denticola_DNF00960	CBM
WP_036856610.1	CBM	CBM32	37.3	Prevotella_denticola_DNF00960	CBM
WP_036854043.1	CBM	CBM32	25.3	Prevotella_denticola_DNF00960	CBM
WP_036853876.1	CBM	CBM32	21.6	Prevotella_denticola_DNF00960	CBM
WP_036856413.1	CBM	CBM35	25.2	Prevotella_denticola_DNF00960	CBM
WP_036854358.1	CBM	CBM35	15.9	Prevotella_denticola_DNF00960	CBM
WP_036856532.1	CBM	CBM39	13.7	Prevotella_denticola_DNF00960	CBM
WP_081940829.1	CBM	CBM40	21.6	Prevotella_denticola_DNF00960	CBM
WP_036856504.1	CBM	CBM40	17.1	Prevotella_denticola_DNF00960	CBM
WP_036853772.1	CBM	CBM47	23.3	Prevotella_denticola_DNF00960	CBM
WP_036854991.1	CBM	CBM47	13.5	Prevotella_denticola_DNF00960	CBM
WP_036855906.1	CBM	CBM48	49.1	Prevotella_denticola_DNF00960	CBM
WP_036856957.1	CBM	CBM48	27.8	Prevotella_denticola_DNF00960	CBM
WP_036857123.1	CBM	CBM50	50.1	Prevotella_denticola_DNF00960	CBM
WP_036856647.1	CBM	CBM50	30.1	Prevotella_denticola_DNF00960	CBM
WP_025067148.1	CBM	CBM50	28.4	Prevotella_denticola_DNF00960	CBM
WP_036853964.1	CBM	CBM58	14.5	Prevotella_denticola_DNF00960	CBM
WP_036853772.1	CBM	CBM62	18.9	Prevotella_denticola_DNF00960	CBM
WP_036853719.1	CBM	CBM8	13.4	Prevotella_denticola_DNF00960	CBM
WP_036854995.1	CBM	CBM9	25.4	Prevotella_denticola_DNF00960	CBM
WP_036853335.1	CBM	CBM66	23.3	Prevotella_denticola_DNF00960	CBM
WP_036855109.1	CBM	CBM67	34.5	Prevotella_denticola_DNF00960	CBM
WP_036855409.1	CBM	CBM67	30.7	Prevotella_denticola_DNF00960	CBM
WP_036853432.1	CBM	CBM67	26.1	Prevotella_denticola_DNF00960	CBM
WP_036883072.1	CBM	CBM13	18.3	Prevotella_disiens_DNF00882	CBM
WP_036884779.1	CBM	CBM20	81.6	Prevotella_disiens_DNF00882	CBM
WP_036884749.1	CBM	CBM27	16.0	Prevotella_disiens_DNF00882	CBM
WP_004358040.1	CBM	CBM32	15.5	Prevotella_disiens_DNF00882	CBM
WP_004358056.1	CBM	CBM32	15.3	Prevotella_disiens_DNF00882	CBM
WP_156097416.1	CBM	CBM48	40.0	Prevotella_disiens_DNF00882	CBM
WP_081938296.1	CBM	CBM4	15.6	Prevotella_disiens_DNF00882	CBM
WP_036881838.1	CBM	CBM50	52.3	Prevotella_disiens_DNF00882	CBM
WP_036882786.1	CBM	CBM50	32.7	Prevotella_disiens_DNF00882	CBM
WP_004356496.1	CBM	CBM50	26.9	Prevotella_disiens_DNF00882	CBM

WP_036884205.1	CBM	CBM50	13.4	Prevotella_disiens_DNF00882	CBM
WP_036883072.1	CBM	CBM56	25.8	Prevotella_disiens_DNF00882	CBM
WP_036882294.1	CBM	CBM69	13.7	Prevotella_disiens_DNF00882	CBM
WP_021669087.1	CBM	CBM72	16.1	Prevotella_disiens_DNF00882	CBM
WP_004355351.1	CBM	CBM84	14.5	Prevotella_disiens_DNF00882	CBM
WP_004355351.1	CBM	CBM85	14.5	Prevotella_disiens_DNF00882	CBM
WP_036928886.1	CBM	CBM16	26.9	Prevotella_timonensis_S9-PR14	CBM
WP_036928905.1	CBM	CBM16	17.5	Prevotella_timonensis_S9-PR14	CBM
WP_036925898.1	CBM	CBM20	56.2	Prevotella_timonensis_S9-PR14	CBM
WP_036926583.1	CBM	CBM22	22.8	Prevotella_timonensis_S9-PR14	CBM
WP_036928886.1	CBM	CBM22	15.7	Prevotella_timonensis_S9-PR14	CBM
WP_036928905.1	CBM	CBM22	13.8	Prevotella_timonensis_S9-PR14	CBM
WP_036925930.1	CBM	CBM26	22.2	Prevotella_timonensis_S9-PR14	CBM
WP_036929191.1	CBM	CBM32	88.9	Prevotella_timonensis_S9-PR14	CBM
WP_036929839.1	CBM	CBM32	84.4	Prevotella_timonensis_S9-PR14	CBM
WP_156104831.1	CBM	CBM32	59.1	Prevotella_timonensis_S9-PR14	CBM
WP_036925589.1	CBM	CBM32	51.1	Prevotella_timonensis_S9-PR14	CBM
WP_036926994.1	CBM	CBM32	48.7	Prevotella_timonensis_S9-PR14	CBM
WP_036928639.1	CBM	CBM32	46.7	Prevotella_timonensis_S9-PR14	CBM
WP_036928585.1	CBM	CBM32	43.8	Prevotella_timonensis_S9-PR14	CBM
WP_036930062.1	CBM	CBM32	42.0	Prevotella_timonensis_S9-PR14	CBM
WP_036926340.1	CBM	CBM32	30.5	Prevotella_timonensis_S9-PR14	CBM
WP_036926172.1	CBM	CBM36	16.3	Prevotella_timonensis_S9-PR14	CBM
WP_008446599.1	CBM	CBM3	14.0	Prevotella_timonensis_S9-PR14	CBM
WP_036926583.1	CBM	CBM40	21.0	Prevotella_timonensis_S9-PR14	CBM
WP_036925862.1	CBM	CBM40	13.1	Prevotella_timonensis_S9-PR14	CBM
WP_036929191.1	CBM	CBM47	33.4	Prevotella_timonensis_S9-PR14	CBM
WP_036926994.1	CBM	CBM47	24.3	Prevotella_timonensis_S9-PR14	CBM
WP_036929839.1	CBM	CBM47	22.7	Prevotella_timonensis_S9-PR14	CBM
WP_156104831.1	CBM	CBM47	20.9	Prevotella_timonensis_S9-PR14	CBM
WP_036928639.1	CBM	CBM47	17.5	Prevotella_timonensis_S9-PR14	CBM
WP_036926340.1	CBM	CBM47	17.4	Prevotella_timonensis_S9-PR14	CBM
WP_036928585.1	CBM	CBM47	15.9	Prevotella_timonensis_S9-PR14	CBM
WP_036926959.1	CBM	CBM48	45.6	Prevotella_timonensis_S9-PR14	CBM
WP_036925926.1	CBM	CBM48	22.6	Prevotella_timonensis_S9-PR14	CBM
WP_052045936.1	CBM	CBM48	15.5	Prevotella_timonensis_S9-PR14	CBM
WP_036925750.1	CBM	CBM50	54.2	Prevotella_timonensis_S9-PR14	CBM
WP_036929830.1	CBM	CBM50	41.2	Prevotella_timonensis_S9-PR14	CBM
WP_156104758.1	CBM	CBM50	35.8	Prevotella_timonensis_S9-PR14	CBM
WP_008123503.1	CBM	CBM50	33.5	Prevotella_timonensis_S9-PR14	CBM
WP_036926195.1	CBM	CBM50	24.6	Prevotella_timonensis_S9-PR14	CBM
WP_036929223.1	CBM	CBM61	18.6	Prevotella_timonensis_S9-PR14	CBM
WP_036927320.1	CBM	CBM61	16.3	Prevotella_timonensis_S9-PR14	CBM
WP_036929191.1	CBM	CBM62	33.7	Prevotella_timonensis_S9-PR14	CBM
WP_036928639.1	CBM	CBM62	15.7	Prevotella_timonensis_S9-PR14	CBM
WP_036926340.1	CBM	CBM62	13.5	Prevotella_timonensis_S9-PR14	CBM
WP_036926788.1	CBM	CBM9	41.1	Prevotella_timonensis_S9-PR14	CBM
WP_036929841.1	CBM	CBM67	69.6	Prevotella_timonensis_S9-PR14	CBM
WP_036926994.1	CBM	CBM67	28.5	Prevotella_timonensis_S9-PR14	CBM
WP_036926398.1	CBM	CBM67	27.8	Prevotella_timonensis_S9-PR14	CBM
WP_036929191.1	CBM	CBM67	26.6	Prevotella_timonensis_S9-PR14	CBM
WP_036928742.1	CBM	CBM67	21.4	Prevotella_timonensis_S9-PR14	CBM
WP_156104831.1	CBM	CBM67	19.9	Prevotella_timonensis_S9-PR14	CBM
WP_036927014.1	CBM	CBM82	13.6	Prevotella_timonensis_S9-PR14	CBM
WP_000462143.1	CBM	CBM34	14.0	Streptococcus_agalactiae_UMB0049	CBM
WP_000370747.1	CBM	CBM41	65.5	Streptococcus_agalactiae_UMB0049	CBM
WP_000066082.1	CBM	CBM48	61.1	Streptococcus_agalactiae_UMB0049	CBM
WP_000370747.1	CBM	CBM48	49.4	Streptococcus_agalactiae_UMB0049	CBM
WP_000143481.1	CBM	CBM50	49.6	Streptococcus_agalactiae_UMB0049	CBM
WP_000029069.1	CBM	CBM50	48.4	Streptococcus_agalactiae_UMB0049	CBM
WP_000783419.1	CBM	CBM50	27.7	Streptococcus_agalactiae_UMB0049	CBM
WP_001085534.1	CBM	CBM50	19.5	Streptococcus_agalactiae_UMB0049	CBM
WP_000403414.1	CBM	CBM70	206.3	Streptococcus_agalactiae_UMB0049	CBM
WP_000462143.1	CBM	CBM34	14.0	Streptococcus_agalactiae_UMB0767	CBM
WP_000823025.1	CBM	CBM41	142.1	Streptococcus_agalactiae_UMB0767	CBM
WP_000370747.1	CBM	CBM41	65.5	Streptococcus_agalactiae_UMB0767	CBM
WP_001032313.1	CBM	CBM42	13.1	Streptococcus_agalactiae_UMB0767	CBM
WP_000066081.1	CBM	CBM48	61.1	Streptococcus_agalactiae_UMB0767	CBM
WP_000370747.1	CBM	CBM48	49.4	Streptococcus_agalactiae_UMB0767	CBM
WP_000823025.1	CBM	CBM48	40.9	Streptococcus_agalactiae_UMB0767	CBM
WP_000029067.1	CBM	CBM50	48.4	Streptococcus_agalactiae_UMB0767	CBM
WP_000783424.1	CBM	CBM50	27.7	Streptococcus_agalactiae_UMB0767	CBM
WP_001085536.1	CBM	CBM50	19.6	Streptococcus_agalactiae_UMB0767	CBM
WP_000403395.1	CBM	CBM70	193.5	Streptococcus_agalactiae_UMB0767	CBM
WP_000462143.1	CBM	CBM34	14.0	Streptococcus_agalactiae_UMB0776	CBM
WP_000823025.1	CBM	CBM41	142.1	Streptococcus_agalactiae_UMB0776	CBM
WP_000370747.1	CBM	CBM41	65.5	Streptococcus_agalactiae_UMB0776	CBM
WP_001032313.1	CBM	CBM42	13.1	Streptococcus_agalactiae_UMB0776	CBM
WP_000066081.1	CBM	CBM48	61.1	Streptococcus_agalactiae_UMB0776	CBM
WP_000370747.1	CBM	CBM48	49.4	Streptococcus_agalactiae_UMB0776	CBM
WP_000823025.1	CBM	CBM48	40.9	Streptococcus_agalactiae_UMB0776	CBM
WP_000029067.1	CBM	CBM50	48.4	Streptococcus_agalactiae_UMB0776	CBM
WP_000783424.1	CBM	CBM50	27.7	Streptococcus_agalactiae_UMB0776	CBM
WP_001085536.1	CBM	CBM50	19.6	Streptococcus_agalactiae_UMB0776	CBM
WP_000403395.1	CBM	CBM70	193.5	Streptococcus_agalactiae_UMB0776	CBM
WP_101749815.1	CBM	CBM20	91.1	Streptococcus_anginosus_UMB0252	CBM
WP_024051906.1	CBM	CBM20	16.6	Streptococcus_anginosus_UMB0252	CBM

WP_024052606.1	CBM	CBM34	77.6	Streptococcus_anginosus_UMB0252	CBM
WP_024052406.1	CBM	CBM41	152.5	Streptococcus_anginosus_UMB0252	CBM
WP_024051580.1	CBM	CBM41	59.6	Streptococcus_anginosus_UMB0252	CBM
WP_024051906.1	CBM	CBM48	65.9	Streptococcus_anginosus_UMB0252	CBM
WP_024052406.1	CBM	CBM48	47.0	Streptococcus_anginosus_UMB0252	CBM
WP_070242135.1	CBM	CBM48	46.0	Streptococcus_anginosus_UMB0252	CBM
WP_024051580.1	CBM	CBM48	45.1	Streptococcus_anginosus_UMB0252	CBM
WP_070242161.1	CBM	CBM50	49.0	Streptococcus_anginosus_UMB0252	CBM
WP_003031567.1	CBM	CBM50	42.5	Streptococcus_anginosus_UMB0252	CBM
WP_070812124.1	CBM	CBM50	13.7	Streptococcus_anginosus_UMB0252	CBM
WP_070241832.1	CBM	CBM61	77.5	Streptococcus_anginosus_UMB0252	CBM
WP_024051879.1	CBM	CBM64	14.4	Streptococcus_anginosus_UMB0252	CBM
WP_070242135.1	CBM	CBM68	92.4	Streptococcus_anginosus_UMB0252	CBM
WP_083307006.1	CBM	CBM20	91.5	Streptococcus_anginosus_UMB0820	CBM
WP_070815151.1	CBM	CBM20	16.6	Streptococcus_anginosus_UMB0820	CBM
WP_024052406.1	CBM	CBM41	152.5	Streptococcus_anginosus_UMB0820	CBM
WP_070811359.1	CBM	CBM41	59.6	Streptococcus_anginosus_UMB0820	CBM
WP_070815151.1	CBM	CBM48	65.9	Streptococcus_anginosus_UMB0820	CBM
WP_024052406.1	CBM	CBM48	47.0	Streptococcus_anginosus_UMB0820	CBM
WP_070815171.1	CBM	CBM48	46.0	Streptococcus_anginosus_UMB0820	CBM
WP_070811359.1	CBM	CBM48	45.1	Streptococcus_anginosus_UMB0820	CBM
WP_101800532.1	CBM	CBM50	85.6	Streptococcus_anginosus_UMB0820	CBM
WP_003031567.1	CBM	CBM50	42.5	Streptococcus_anginosus_UMB0820	CBM
WP_024051814.1	CBM	CBM50	16.6	Streptococcus_anginosus_UMB0820	CBM
WP_070584292.1	CBM	CBM61	77.5	Streptococcus_anginosus_UMB0820	CBM
WP_070815140.1	CBM	CBM64	14.1	Streptococcus_anginosus_UMB0820	CBM
WP_070815171.1	CBM	CBM68	93.5	Streptococcus_anginosus_UMB0820	CBM
WP_024052610.1	CBM	CBM20	91.1	Streptococcus_anginosus_UMB0839	CBM
WP_024051906.1	CBM	CBM20	16.6	Streptococcus_anginosus_UMB0839	CBM
WP_024052606.1	CBM	CBM34	77.6	Streptococcus_anginosus_UMB0839	CBM
WP_024052406.1	CBM	CBM41	152.5	Streptococcus_anginosus_UMB0839	CBM
WP_024051580.1	CBM	CBM41	59.6	Streptococcus_anginosus_UMB0839	CBM
WP_024051906.1	CBM	CBM48	65.9	Streptococcus_anginosus_UMB0839	CBM
WP_024052406.1	CBM	CBM48	47.0	Streptococcus_anginosus_UMB0839	CBM
WP_024053239.1	CBM	CBM48	46.0	Streptococcus_anginosus_UMB0839	CBM
WP_024051580.1	CBM	CBM48	45.1	Streptococcus_anginosus_UMB0839	CBM
WP_101800944.1	CBM	CBM50	84.3	Streptococcus_anginosus_UMB0839	CBM
WP_003031567.1	CBM	CBM50	42.5	Streptococcus_anginosus_UMB0839	CBM
WP_017646700.1	CBM	CBM50	38.5	Streptococcus_anginosus_UMB0839	CBM
WP_024051814.1	CBM	CBM50	16.6	Streptococcus_anginosus_UMB0839	CBM
WP_070241832.1	CBM	CBM61	77.5	Streptococcus_anginosus_UMB0839	CBM
WP_024051879.1	CBM	CBM64	14.4	Streptococcus_anginosus_UMB0839	CBM
WP_024053239.1	CBM	CBM68	92.4	Streptococcus_anginosus_UMB0839	CBM
WP_023942436.1	CBM	CBM20	18.6	Streptococcus_mitis_CMW7705B	CBM
WP_081094117.1	CBM	CBM32	56.7	Streptococcus_mitis_CMW7705B	CBM
WP_081094120.1	CBM	CBM32	40.9	Streptococcus_mitis_CMW7705B	CBM
WP_060805527.1	CBM	CBM34	109.1	Streptococcus_mitis_CMW7705B	CBM
WP_081094089.1	CBM	CBM37	13.4	Streptococcus_mitis_CMW7705B	CBM
WP_060805197.1	CBM	CBM41	162.1	Streptococcus_mitis_CMW7705B	CBM
WP_060805491.1	CBM	CBM41	31.1	Streptococcus_mitis_CMW7705B	CBM
WP_081094117.1	CBM	CBM47	217.7	Streptococcus_mitis_CMW7705B	CBM
WP_081094120.1	CBM	CBM47	118.0	Streptococcus_mitis_CMW7705B	CBM
WP_023942436.1	CBM	CBM48	68.3	Streptococcus_mitis_CMW7705B	CBM
WP_060805491.1	CBM	CBM48	50.7	Streptococcus_mitis_CMW7705B	CBM
WP_060805197.1	CBM	CBM48	42.3	Streptococcus_mitis_CMW7705B	CBM
WP_060805907.1	CBM	CBM50	46.8	Streptococcus_mitis_CMW7705B	CBM
WP_060806161.1	CBM	CBM50	32.9	Streptococcus_mitis_CMW7705B	CBM
WP_155640150.1	CBM	CBM50	21.9	Streptococcus_mitis_CMW7705B	CBM
WP_081094117.1	CBM	CBM62	17.1	Streptococcus_mitis_CMW7705B	CBM
WP_060805890.1	CBM	CBM66	14.2	Streptococcus_mitis_CMW7705B	CBM
WP_060805979.1	CBM	CBM71	147.8	Streptococcus_mitis_CMW7705B	CBM
WP_060805445.1	CBM	CBM71	17.5	Streptococcus_mitis_CMW7705B	CBM
WP_060627358.1	CBM	CBM32	34.8	Streptococcus_mitis_KCOM1350	CBM
WP_060628324.1	CBM	CBM34	112.8	Streptococcus_mitis_KCOM1350	CBM
WP_080998459.1	CBM	CBM37	14.3	Streptococcus_mitis_KCOM1350	CBM
WP_080998536.1	CBM	CBM40	207.9	Streptococcus_mitis_KCOM1350	CBM
WP_060627448.1	CBM	CBM41	163.5	Streptococcus_mitis_KCOM1350	CBM
WP_060628240.1	CBM	CBM41	18.6	Streptococcus_mitis_KCOM1350	CBM
WP_060628242.1	CBM	CBM48	64.9	Streptococcus_mitis_KCOM1350	CBM
WP_060628240.1	CBM	CBM48	49.5	Streptococcus_mitis_KCOM1350	CBM
WP_060627448.1	CBM	CBM48	42.3	Streptococcus_mitis_KCOM1350	CBM
WP_060627375.1	CBM	CBM50	47.7	Streptococcus_mitis_KCOM1350	CBM
WP_060627224.1	CBM	CBM50	33.9	Streptococcus_mitis_KCOM1350	CBM
WP_060628606.1	CBM	CBM50	16.9	Streptococcus_mitis_KCOM1350	CBM
WP_060627357.1	CBM	CBM71	158.1	Streptococcus_mitis_KCOM1350	CBM
WP_060628368.1	CBM	CBM78	13.0	Streptococcus_mitis_KCOM1350	CBM
WP_101782605.1	CBM	CBM32	43.8	Streptococcus_mitis_UMB0079	CBM
WP_101781950.1	CBM	CBM34	114.0	Streptococcus_mitis_UMB0079	CBM
WP_101782322.1	CBM	CBM40	212.4	Streptococcus_mitis_UMB0079	CBM
WP_101782140.1	CBM	CBM41	164.3	Streptococcus_mitis_UMB0079	CBM
WP_101781905.1	CBM	CBM41	31.8	Streptococcus_mitis_UMB0079	CBM
WP_101782605.1	CBM	CBM47	117.3	Streptococcus_mitis_UMB0079	CBM
WP_101781909.1	CBM	CBM48	67.4	Streptococcus_mitis_UMB0079	CBM
WP_101781905.1	CBM	CBM48	52.8	Streptococcus_mitis_UMB0079	CBM
WP_101782140.1	CBM	CBM48	42.2	Streptococcus_mitis_UMB0079	CBM
WP_101782075.1	CBM	CBM50	46.8	Streptococcus_mitis_UMB0079	CBM
WP_101782637.1	CBM	CBM50	33.9	Streptococcus_mitis_UMB0079	CBM

WP_101782391.1	CBM	CBM71	149.2	Streptococcus_mitis_UMB0079	CBM
WP_101781872.1	CBM	CBM71	18.3	Streptococcus_mitis_UMB0079	CBM
WP_101784853.1	CBM	CBM20	18.6	Streptococcus_mitis_UMB1341	CBM
WP_101784689.1	CBM	CBM32	58.1	Streptococcus_mitis_UMB1341	CBM
WP_101784730.1	CBM	CBM32	43.1	Streptococcus_mitis_UMB1341	CBM
WP_101781950.1	CBM	CBM34	114.0	Streptococcus_mitis_UMB1341	CBM
WP_101786053.1	CBM	CBM40	210.8	Streptococcus_mitis_UMB1341	CBM
WP_101785495.1	CBM	CBM41	165.3	Streptococcus_mitis_UMB1341	CBM
WP_101784851.1	CBM	CBM41	28.8	Streptococcus_mitis_UMB1341	CBM
WP_101784689.1	CBM	CBM47	218.3	Streptococcus_mitis_UMB1341	CBM
WP_101784730.1	CBM	CBM47	115.1	Streptococcus_mitis_UMB1341	CBM
WP_101784853.1	CBM	CBM48	67.2	Streptococcus_mitis_UMB1341	CBM
WP_101784851.1	CBM	CBM48	49.7	Streptococcus_mitis_UMB1341	CBM
WP_101785495.1	CBM	CBM48	42.3	Streptococcus_mitis_UMB1341	CBM
WP_101782075.1	CBM	CBM50	46.8	Streptococcus_mitis_UMB1341	CBM
WP_061759600.1	CBM	CBM50	34.0	Streptococcus_mitis_UMB1341	CBM
WP_101785908.1	CBM	CBM50	20.5	Streptococcus_mitis_UMB1341	CBM
WP_101784689.1	CBM	CBM62	17.0	Streptococcus_mitis_UMB1341	CBM
WP_101784425.1	CBM	CBM66	16.0	Streptococcus_mitis_UMB1341	CBM
WP_101785698.1	CBM	CBM67	18.3	Streptococcus_mitis_UMB1341	CBM
WP_101786293.1	CBM	CBM71	149.1	Streptococcus_mitis_UMB1341	CBM
WP_101784819.1	CBM	CBM71	18.2	Streptococcus_mitis_UMB1341	CBM
WP_049498797.1	CBM	CBM32	48.5	Streptococcus_parasanguinis_UMB0216	CBM
WP_101770529.1	CBM	CBM34	110.6	Streptococcus_parasanguinis_UMB0216	CBM
WP_049498797.1	CBM	CBM35	19.0	Streptococcus_parasanguinis_UMB0216	CBM
WP_101770501.1	CBM	CBM41	48.8	Streptococcus_parasanguinis_UMB0216	CBM
WP_014713083.1	CBM	CBM48	67.8	Streptococcus_parasanguinis_UMB0216	CBM
WP_101770501.1	CBM	CBM48	42.9	Streptococcus_parasanguinis_UMB0216	CBM
WP_101770853.1	CBM	CBM48	42.4	Streptococcus_parasanguinis_UMB0216	CBM
WP_049498743.1	CBM	CBM50	45.0	Streptococcus_parasanguinis_UMB0216	CBM
WP_049498329.1	CBM	CBM50	41.1	Streptococcus_parasanguinis_UMB0216	CBM
WP_049499136.1	CBM	CBM50	37.9	Streptococcus_parasanguinis_UMB0216	CBM
WP_003015783.1	CBM	CBM50	17.1	Streptococcus_parasanguinis_UMB0216	CBM
WP_049498797.1	CBM	CBM6	21.1	Streptococcus_parasanguinis_UMB0216	CBM
WP_101770853.1	CBM	CBM68	43.6	Streptococcus_parasanguinis_UMB0216	CBM
WP_101771071.1	CBM	CBM71	502.8	Streptococcus_parasanguinis_UMB0216	CBM
WP_049499812.1	CBM	CBM71	16.5	Streptococcus_parasanguinis_UMB0216	CBM
WP_049497549.1	CBM	CBM75	13.7	Streptococcus_parasanguinis_UMB0216	CBM
WP_002886869.1	CBM	CBM20	15.5	Streptococcus_salivarius_UMB0051	CBM
WP_064520524.1	CBM	CBM35	34.1	Streptococcus_salivarius_UMB0051	CBM
WP_064520797.1	CBM	CBM41	155.1	Streptococcus_salivarius_UMB0051	CBM
WP_101799704.1	CBM	CBM41	55.3	Streptococcus_salivarius_UMB0051	CBM
WP_002886869.1	CBM	CBM48	59.2	Streptococcus_salivarius_UMB0051	CBM
WP_101799704.1	CBM	CBM48	50.5	Streptococcus_salivarius_UMB0051	CBM
WP_064520797.1	CBM	CBM48	44.6	Streptococcus_salivarius_UMB0051	CBM
WP_002892168.1	CBM	CBM50	49.8	Streptococcus_salivarius_UMB0051	CBM
WP_064520593.1	CBM	CBM50	36.2	Streptococcus_salivarius_UMB0051	CBM
WP_014635064.1	CBM	CBM50	31.4	Streptococcus_salivarius_UMB0051	CBM
WP_048790727.1	CBM	CBM50	30.8	Streptococcus_salivarius_UMB0051	CBM
WP_021144241.1	CBM	CBM50	21.9	Streptococcus_salivarius_UMB0051	CBM
WP_064520668.1	CBM	CBM66	262.2	Streptococcus_salivarius_UMB0051	CBM
WP_014635039.1	CBM	CBM66	24.5	Streptococcus_salivarius_UMB0051	CBM
WP_014634730.1	CBM	CBM67	20.5	Streptococcus_salivarius_UMB0051	CBM
WP_038675252.1	CBM	CBM67	16.3	Streptococcus_salivarius_UMB0051	CBM
WP_064521293.1	CBM	CBM72	16.7	Streptococcus_salivarius_UMB0051	CBM
WP_011836900.1	CBM	CBM12	18.5	Streptococcus_sanguinis_SK36	CBM
WP_002897469.1	CBM	CBM12	12.7	Streptococcus_sanguinis_SK36	CBM
WP_011836715.1	CBM	CBM20	16.4	Streptococcus_sanguinis_SK36	CBM
WP_033179178.1	CBM	CBM34	112.3	Streptococcus_sanguinis_SK36	CBM
WP_011836521.1	CBM	CBM41	157.1	Streptococcus_sanguinis_SK36	CBM
WP_011837170.1	CBM	CBM41	55.4	Streptococcus_sanguinis_SK36	CBM
WP_011837260.1	CBM	CBM46	14.3	Streptococcus_sanguinis_SK36	CBM
WP_011836715.1	CBM	CBM48	62.5	Streptococcus_sanguinis_SK36	CBM
WP_011836521.1	CBM	CBM48	46.7	Streptococcus_sanguinis_SK36	CBM
WP_011837170.1	CBM	CBM48	44.9	Streptococcus_sanguinis_SK36	CBM
WP_011837658.1	CBM	CBM48	40.7	Streptococcus_sanguinis_SK36	CBM
WP_011836324.1	CBM	CBM50	40.3	Streptococcus_sanguinis_SK36	CBM
WP_002894257.1	CBM	CBM50	38.2	Streptococcus_sanguinis_SK36	CBM
WP_004188880.1	CBM	CBM50	33.0	Streptococcus_sanguinis_SK36	CBM
WP_002897255.1	CBM	CBM50	17.4	Streptococcus_sanguinis_SK36	CBM
WP_011837695.1	CBM	CBM56	15.5	Streptococcus_sanguinis_SK36	CBM
WP_011836900.1	CBM	CBM5	15.4	Streptococcus_sanguinis_SK36	CBM
WP_011837502.1	CBM	CBM66	226.2	Streptococcus_sanguinis_SK36	CBM
WP_011837658.1	CBM	CBM68	89.6	Streptococcus_sanguinis_SK36	CBM
WP_011837479.1	CBM	CBM71	18.5	Streptococcus_sanguinis_SK36	CBM
